# Itaconimides as Novel Quorum Sensing Inhibitors of *Pseudomonas aeruginosa*

**DOI:** 10.3389/fcimb.2018.00443

**Published:** 2019-01-07

**Authors:** July Fong, Kim T. Mortensen, Amalie Nørskov, Katrine Qvortrup, Liang Yang, Choon Hong Tan, Thomas E. Nielsen, Michael Givskov

**Affiliations:** ^1^Singapore Centre for Environmental Life Sciences Engineering, Nanyang Technological University, Singapore, Singapore; ^2^Department of Chemistry, Technical University of Denmark, Lyngby, Denmark; ^3^Southern University of Science and Technology, Shenzhen, China; ^4^Division of Chemistry and Biological Chemistry, School of Physical and Mathematical Sciences, Nanyang Technological University, Singapore, Singapore; ^5^Department of Immunology and Microbiology, Costerton Biofilm Center, University of Copenhagen, Copenhagen, Denmark

**Keywords:** quorum sensing, biofilm, itaconimides, antivirulence, chemical biology

## Abstract

*Pseudomonas aeruginosa* is known as an opportunistic pathogen that often causes persistent infections associated with high level of antibiotic-resistance and biofilms formation. Chemical interference with bacterial cell-to-cell communication, termed quorum sensing (QS), has been recognized as an attractive approach to control infections and address the drug resistance problems currently observed worldwide. Instead of imposing direct selective pressure on bacterial growth, the right bioactive compounds can preferentially block QS-based communication and attenuate cascades of bacterial gene expression and production of virulence factors, thus leading to reduced pathogenicity. Herein, we report on the potential of itaconimides as quorum sensing inhibitors (QSI) of *P. aeruginosa*. An initial hit was discovered in a screening program of an in-house compound collection, and subsequent structure-activity relationship (SAR) studies provided analogs that could reduce expression of central QS-regulated virulence factors (elastase, rhamnolipid, and pyocyanin), and also successfully lead to the eradication of *P. aeruginosa* biofilms in combination with tobramycin. Further studies on the cytotoxicity of compounds using murine macrophages indicated no toxicity at common working concentrations, thereby pointing to the potential of these small molecules as promising entities for antimicrobial drug development.

## Introduction

*Pseudomonas aeruginosa* is an opportunistic pathogen and a major cause of nosocomical infections in patients with pneumonia, chronic wounds, urinary tract infections, and intensive care units (ICUs) (Vincent et al., 1995). As an opportunistic pathogen, this organism is highly adaptive, versatile and exhibits remarkable resistance toward many antimicrobial agents. Resistance is a distinctive characteristic of *P. aeruginosa*, due to its ability to express multiple resistance mechanisms, including enzymes and efflux pumps (Poole, 2001; Lister et al., 2009). U.S. Centers for Disease Control and Prevention (CDC) (2013) estimated that more than 20,000 deaths per year are attributed to antibiotic resistance cases. It has become a global issue, and we are threatened with the slow progress of new antibiotics development and lack of preventive measure for the spread of resistance.

In addition, *P. aeruginosa* is well-known to form biofilms, which have been identified as a major underlying cause of persistent infections in immunocompromised patients, chronic wounds as well as on medical devices like implants, catheters, tubes, artificial hip, and many more (Costerton et al., 1999). Biofilms infections are often characterized by their broad range resistance toward host defense mechanisms and antibiotic therapy. This results in prolonged treatment, complications in clinical outcomes, and additional socio-economic burdens. In cystic fibrosis patients, chronic biofilm infections of *P. aeruginosa* can cause premature death despite intensive antibiotic therapy care (Bjarnsholt et al., 2009).

The bacteria in biofilms often exhibit different phenotypic and genetic variants as compared to their planktonic counterparts. In the biofilm mode of life, bacterial cells are enclosed within a matrix of extracellular polymeric substances (EPS) comprises of exopolysaccharides, proteins, deoxyribonucleic acid (DNA), lipids or surfactants, and macromolecules that are self-produced by the cells (Flemming and Wingender, 2010). All of these could render antibiotics impenetrable, chelated or sequestered, and diminish the efficacy of the treatment. The presence of persister cells in biofilms also contributes to multidrug resistance property of biofilms (Lewis, 2007). Overall, the complex biology of biofilms represents a tremendous challenge to develop therapeutic agents that could successfully prevent or eradicate biofilms-associated infections.

The cell-to-cell communication system called quorum sensing (QS) has been reported to play major roles for establishing persistent, biofilm based infections (Hentzer et al., 2003; Alhede et al., 2009; Van Gennip et al., 2009; Chiang et al., 2013). The QS system in *P. aeruginosa* utilizes acyl homoserine lactones (AHLs) as signal molecules and comprises the Lux homologs LasRI and RhlRI. LasI synthase is responsible for the synthesis of *N*-(3-oxododecanoyl) homoserine lactone (3-oxo-C12-HSL), which will bind to its receptor LasR and activate transcription of genes responsible for virulence such as *lasB, apr*, and *toxA* (Gambello et al., 1993; Passador et al., 1993). The *las* system also positively regulates *rhl* system, where RhlI directs the synthesis of *N-*butanoylhomoserine lactone (C4-HSL) that would bind to its receptor RhlR and subsequently activate gene expression of QS target genes (Brint and Ohman, 1995; Pearson et al., 1995; Pesci et al., 1997). In addition, there is also a third signaling molecule “pseudomonas quinolone signal” (PQS) which is intertwined between the *las* and *rhl* systems (Schertzer et al., 2009). Recently, a fourth signal molecules called Integrative Quorum Sensing Signal (IQS) has been reported, which could overtake the central *las* system under phosphate depletion condition (Lee et al., 2013). QS defective *P. aeruginosa* mutants are attenuated as compared to the wild-type strain, and their biofilms are more susceptible toward antibiotics treatment and host immune system as compared to the wild-type (Pearson et al., 2000; Hentzer et al., 2002, 2003).

As QS governs various patterns of genes expression to control virulence and biofilm formation, it has been proposed that interfering with the communication system could be a promising strategy for the control and prevention of bacterial infections (Hentzer et al., 2003). Quorum sensing inhibitors (QSI) are compounds that interfere with QS pathways, reduce expression of QS-controlled genes and attenuate infecting bacteria. As such compounds do not affect the growth of bacteria, these molecular entities pose lower selective pressure on bacteria and lower the risk of resistance development. Recent exploration of new classes of QSI comprises natural products, synthetic molecules, and enzymes that may quench or inactivate QS signals (Dong et al., 2001; Hentzer et al., 2002; Jakobsen et al., 2012; Fong et al., 2017). Unfortunately, no clinical candidates have yet been developed for therapy.

In the present study, we report a new class of small molecules that disrupt QS pathways in *P. aeruginosa*. The structurally related itaconimides and citraconimides have previously been reported to inhibit growth of mycobacteria (Balganesh et al., 1999). We synthesized a range of small molecules by an iterative structure-activity relationship (SAR) study and found two promising candidates for further biological investigation. The efficacies of these compounds were tested on *P. aeruginosa* QS bioreporter strains (*lasB-gfp; rhlA-gfp; pqsA-gfp*) and also on QS-controlled virulence phenotypes, such as elastase, pyocyanin, and rhamnolipid production.

## Materials and Methods

### General Information

All chemicals were purchased from Sigma Aldrich and used without further purification. For biological studies, synthesized compounds were prepared in DMSO as 10 mM stock solution and stored at −20°C until further usage. Overnight culture of bacteria was grown in Lysogeny broth (LB) which consisted of 1% tryptone, 0.5% yeast extract, 0.5% NaCl. For bioreporter assay, strains were grown in ABTGC (AB minimal medium supplemented with 0.2% glucose and 0.2% casamino acids) (Clark and Maaloe, 1967) to minimize fluorescence interference. ABTG (with no casamino acid) medium was used for biofilms study in flow chambers. Strains used in this study can be found in Table 1. Summary of chemical synthesis and spectroscopy data can be found in Supplementary Material.

**Table 1:** Bacterial strains used in this study.

**Strains or plasmids**	**Relevant genotype and/or characteristics^a^**
**Strains**	
PAO1	ATCC *Pseudomonas aeruginosa* (Hentzer et al., 2002)
PAO1-*gfp*	GFP-tagged wild-type *Pseudomonas aeruginosa* (Yang et al., 2007)
PAO1-*lasB-gfp*	PAO1 containing *lasB-gfp*(ASV) reporter fusion (Hentzer et al., 2002)
PAO1-*rhlA-gfp*	PAO1 containing *rhlA-gfp*(ASV) reporter fusion (Yang et al., 2007)
PAO1-*pqsA-gfp*	PAO1 containing *pqsA-gfp*(ASV) reporter fusion (Yang et al., 2009)
PAO1 Δ*lasI*Δ*rhlI*	Gm^a^; PAO1 *lasI and rhlI* mutant (Hentzer et al., 2003)
PAO1 Δ*lasR*	PAO1 *lasR* mutant (Hentzer et al., 2003)
Δ*lasR-rhlA-gfp*	PAO1 *lasR* mutant containing *rhlA-gfp*(ASV) reporter fusion (Tan et al., 2013)
Δ*lasR-pqsA-gfp*	PAO1 *lasR* mutant containing *pqsA-gfp*(ASV) reporter fusion (Tan et al., 2013)

a *Description of the strains' antibiotic resistance. Gm, gentamicin*.

### QS Inhibition Assay

From its frozen stock, compounds were diluted appropriately to their working concentration in ABTGC medium. Experiments were done as previously reported (Fong et al., 2017). Briefly, 200 μL of compounds was pipetted into the first rows of 96-well plates (Nunc, Denmark), followed by two-fold serial dilution to the rest of the rows. The last two rows were allocated to solvent control (DMSO 0.1%) and blank (media control). Overnight cultures of *P. aeruginosa* bioreporter strains were diluted to optical density at 600 nm (OD_600_) of 0.02 (~2.5 × 10^8^ CFU/mL). Next, 100 μL of the bacteria culture was added into each well to make final OD_600_ of 0.01. The plate was incubated at 37°C for 16 h, with time-point measurement of GFP fluorescence (excitation 485 nm, emission 535 nm) and OD_600_ recorded at every 15 min using Tecan Infinite 200 Pro plate reader (Tecan Group Ltd, Männedorf, Switzerland). The data were exported into excel files, and IC_50_ value calculation was determined using GraphPad Prism 6 software. For IC_50_ values determination, the GFP/OD_600_ values were taken at the time point between 4 and 6 h, where inhibition started to occur. All experiments were done in triplicate manner and repeated at least twice to confirm the results.

### QS-Regulated Virulence Factor Assays

Elastase activity was measured using EnzChekElastase kit (Invitrogen, USA), following the manufacturer's instruction. Rhamnolipid was extracted and quantified using method reported by Koch et al. with modifications (Koch et al., 1991). Briefly, overnight cultures of *P. aeruginosa* were diluted into ABTGC medium (OD_600_ = 0.01), with and without the presence of compounds (DMSO as control). Cultures were grown overnight at 37°C, 200 rpm. Rhamnolipid was extracted from the supernatant with diethyl ether (twice), and organic fractions were concentrated to yield yellowish-white solids. The solids were re-suspended in deionized water and added with 0.19% (w/v) orcinol in 50% H_2_SO_4_. It was then heated at 80°C for 20–30 min to give dark orange color. Absorbance was measured at 421 nm and the values were normalized with cell density at OD_600_. Pyocyanin was extracted from overnight culture of *P. aeruginosa* grown in Kings Medium A Base [MilliQ water supplemented with proteose peptone (20 g/L), potassium sulfate (10 g/L), magnesium chloride, anhydrous (1.640 g/L), and glycerol (10% v/v)]. Supernatants were collected and extracted with chloroform and 0.2 M HCl. The presence of pyocyanin would turn the HCl solution into pinkish color. Absorbance was measured at 520 nm and normalized with cell density OD_600_ values. Experiments were done in triplicate manner and repeated at least twice to confirm the results.

### Biofilm Experiments

GFP-tagged *P. aeruginosa* were grown in ABTG medium and flowed through flow chambers as previously described (Sternberg and Tolker-Nielsen, 2006). Each flow chamber is consisted of three-channel flow cells that were supplied with a flow of medium and oxygen, while waste medium would be directed into a waste flask. Briefly, overnight cultures were diluted 1,000 times in ABTG medium and injected into each channel for 1 h incubation time without flow. Next, the medium was allowed to flow into the flow cells and the velocity was maintained at 0.2 mm/s using Cole-Palmer peristaltic pump. Biofilms were grown for 72 h before treatment with compounds for further 48 h. To visualize dead cells, 300 μL of propidium iodide (PI) stain was injected into each flow cells. Biofilm images were taken with LSM confocal laser scanning microscope (Carl Zeiss, Germany) at 20x objective lens. Microscopy images were processed with IMARIS software (Bitplane AG, Zurich, Switzerland). Experiments were done in triplicate manner and repeated at least twice to confirm the results.

### Cytotoxicity Assay

Toxicity assay was done as previously reported (Fong et al., 2017). Murine macrophage RAW264.7 cell lines were grown in Dulbecco's Modified Eagle's Medium (DMEM, Life Technologies), supplemented with 10% fetal bovine serum (Gibco) at 37°C and 5% CO_2_. The cells were passaged into 96-well microplates, with each well containing 1 × 10^4^ macrophages. After 16 h, the cells were washed with phosphate-buffered saline (PBS) and treated with compounds at varying concentration in 100 μL DMEM. The plate was incubated at 37°C and 5% CO_2_ for further 16 h. Resazurin was then added into each well to reach final concentration of 10 μM for cell viability measurement. The live cells would be able to convert the dye to red color, whereby dead cells would remain as blue color. Absorbance was taken at 595 nm using Tecan Infinite 200 Pro plate reader (Tecan Group Ltd, Männedorf, Switzerland). Experiments were done in triplicate manner and repeated at least twice to confirm the results.

## Results

### Synthesis and SAR Study

**Scheme 1:**
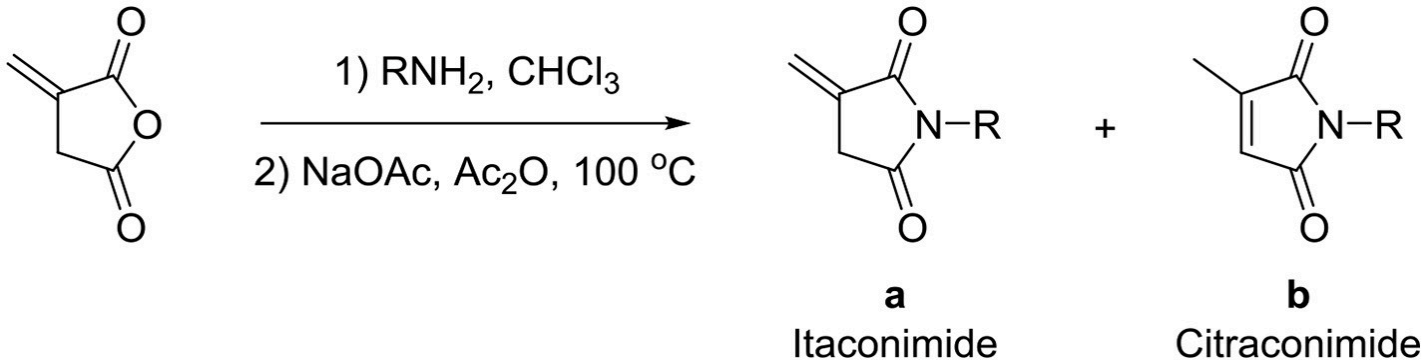
Synthesis of itaconimides and citraconimides.

Recently, we discovered that 3-methylene-1-tetradecylpyrrolidine-2,5-dione (Table 2, **1a**) displayed QSI activity against *P. aeruginosa* QS reporter strain (carrying a *lasB-gfp* fusion). Different variation on the left-hand side of **1a** proved that the exo-cyclic double bond was essential for its biological activity. This led us to focus on the synthesis and biological evaluation of *N*-substituted itaconimide analogs against *P. aeruginosa* (Scheme 1). The procedure for the synthesis of the itaconimides have been described by Cava et al. (1961) and Leow et al. (2008). The commercially available itaconic anhydride was treated with anilines (1.0 equiv.) in CHCl_3_ to afford the corresponding α-itaconamic acids (Kyung et al., 1974). The resulting acids were subsequently treated with Ac_2_O (3.5 equiv.) and NaOAc (0.5 equiv.) at elevated temperature to afford a mixture of itaconimide and the isomerized product citraconimide. Generally, the yield for the aniline derivatives was higher than that of the aliphatic amines. An overview of the synthesized compounds is provided below (Table 2).

**Table 2:** Summary of the synthesized compounds and the corresponding yields.

**Compound**	**R**	**a (%)^a^**	**b (%)^a^**	**Compound**	**R**	**a (%)^a^**	**b (%)^a^**
**1**	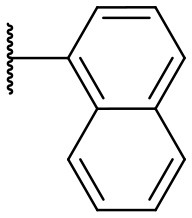	50	14	**10**	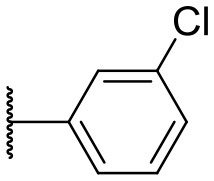	35	15
**2**	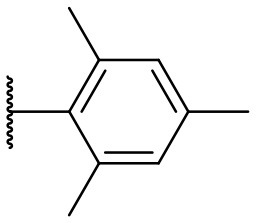	37	–^b^	**11**	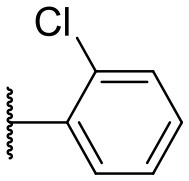	61	9
**3**	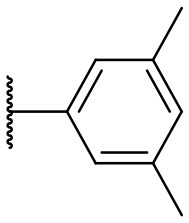	17	–^b^	**12**	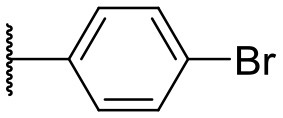	37	20
**4**	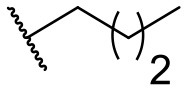	10	–^b^	**13**	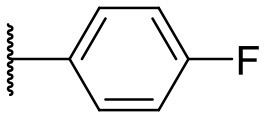	40	19
**5**	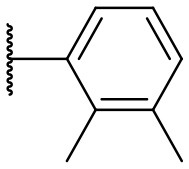	47	–^b^	**14**	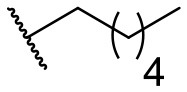	8	14
**6**	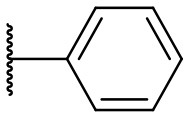	49	29	**15**	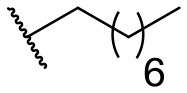	16	–^b^
**7**	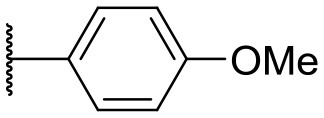	–^c^		**16**	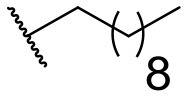	13	10
**8**	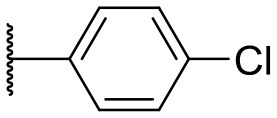	31	40	**17**	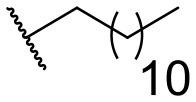	4	12
**9**	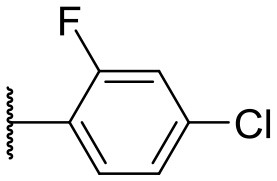	24	14	**18**	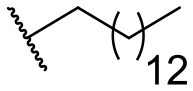	2	11

a *Yields based on starting itatonic anhydride and purified after cyclization by flash column chromatography*.

b *Not isolated*.

c *Commercial sample*.

### QSI Activities of Itaconimides

**Figure 1:**
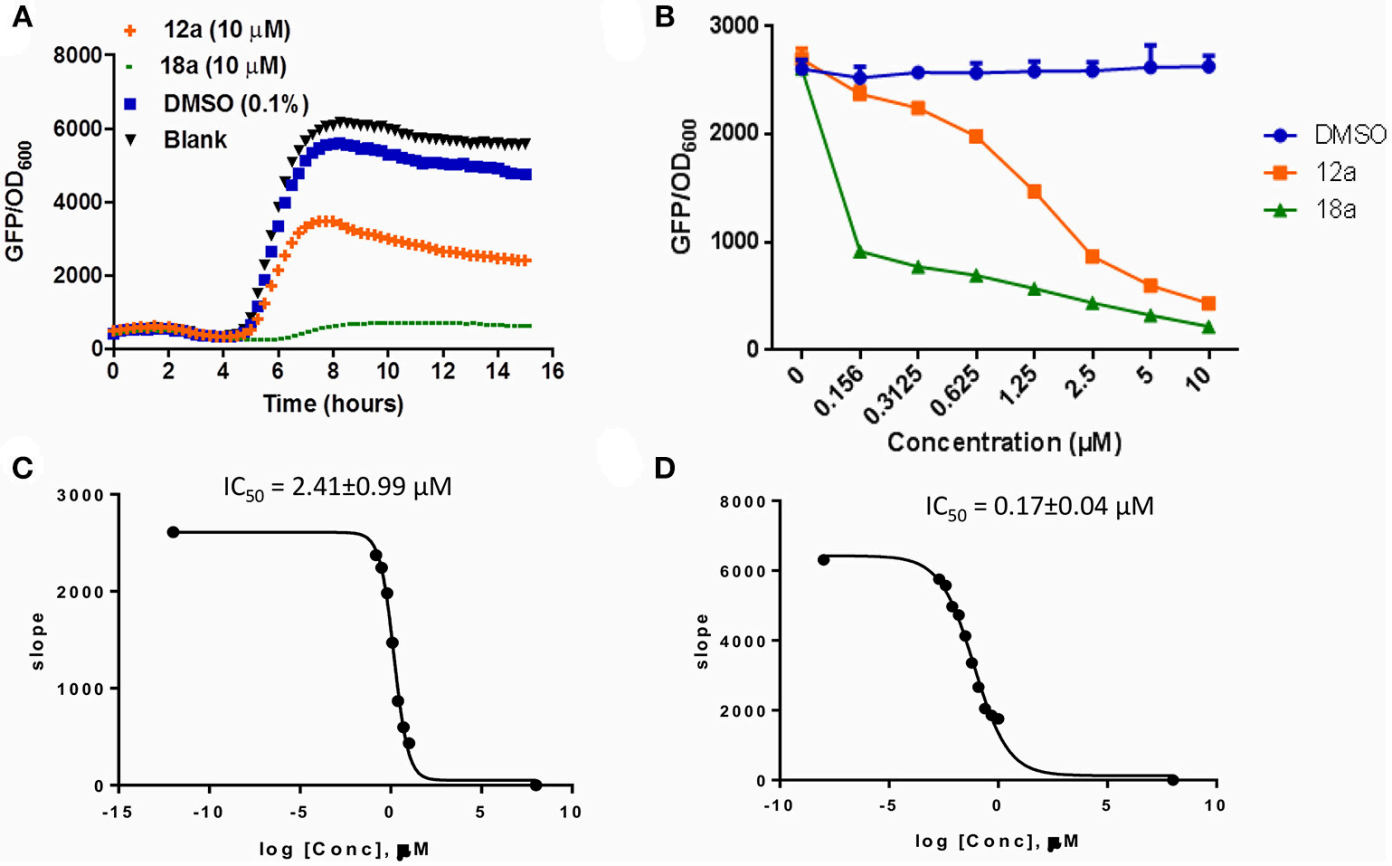
**(A)** Effects of compounds **12a** and **18a** on PAO1-*lasB-gfp* reporter strain. DMSO 0.1% was used as solvent control. **(B)** Dose-response effects of compounds on *P. aeruginosa lasB-gfp*. **(C)** IC_50_ values of **12a** and **18a (D)** on *lasB-gfp*. Calculation was done using GraphPad Prism 6 software, taken at time point between 4 and 6 h when QS inhibition started to occur. All experiments were done in triplicate manner (technical and biological replicates), only representative data are shown.

The synthesized compounds were tested for their QS inhibitory activity against the *P. aeruginosa lasB*-*gfp* reporter strain. None of our synthesized compounds showed antibiotic properties (Supplementary Figures 1, 2). We found that both the *p*-bromophenyl and tetradecyl-substituted itaconimide (**12a** and **18a**) showed strong QS inhibition activity against the *lasB-gfp* reporter strain (Figure 1A). Both compounds inhibit the expression of *lasB-gfp* in a dose-dependent manner (Figure 1B). **18a** was proved to be the most active compound synthesized in this series, with almost 25-fold higher activity (IC_50_) compared to our hit compound (Figures 1C,D).

To address the specificity of our compounds, we also tested our compounds against PAO1 wild-type (WT) and Δ*lasR* harboring either *rhlA-gfp* or *pqsA-gfp* fusions. In this case, we would be able to determine if the compounds could affect other QS pathways in *las-*dependent or independent manner. Both *rhlA* and *pqsA* are the first genes of *rhl* operon and *pqs* operon that code for the production of the rhamnolipid and PQS precursor molecules (Ochsner et al., 1994; Gallagher et al., 2002). To eliminate false positive, we also tested both compounds with a *gfp*-tagged *P. aeruginosa* (expresses GFP constitutively) as control and did not observe any reduction in the fluorescence signals (Supplementary Figure 3).

**Figure 2:**
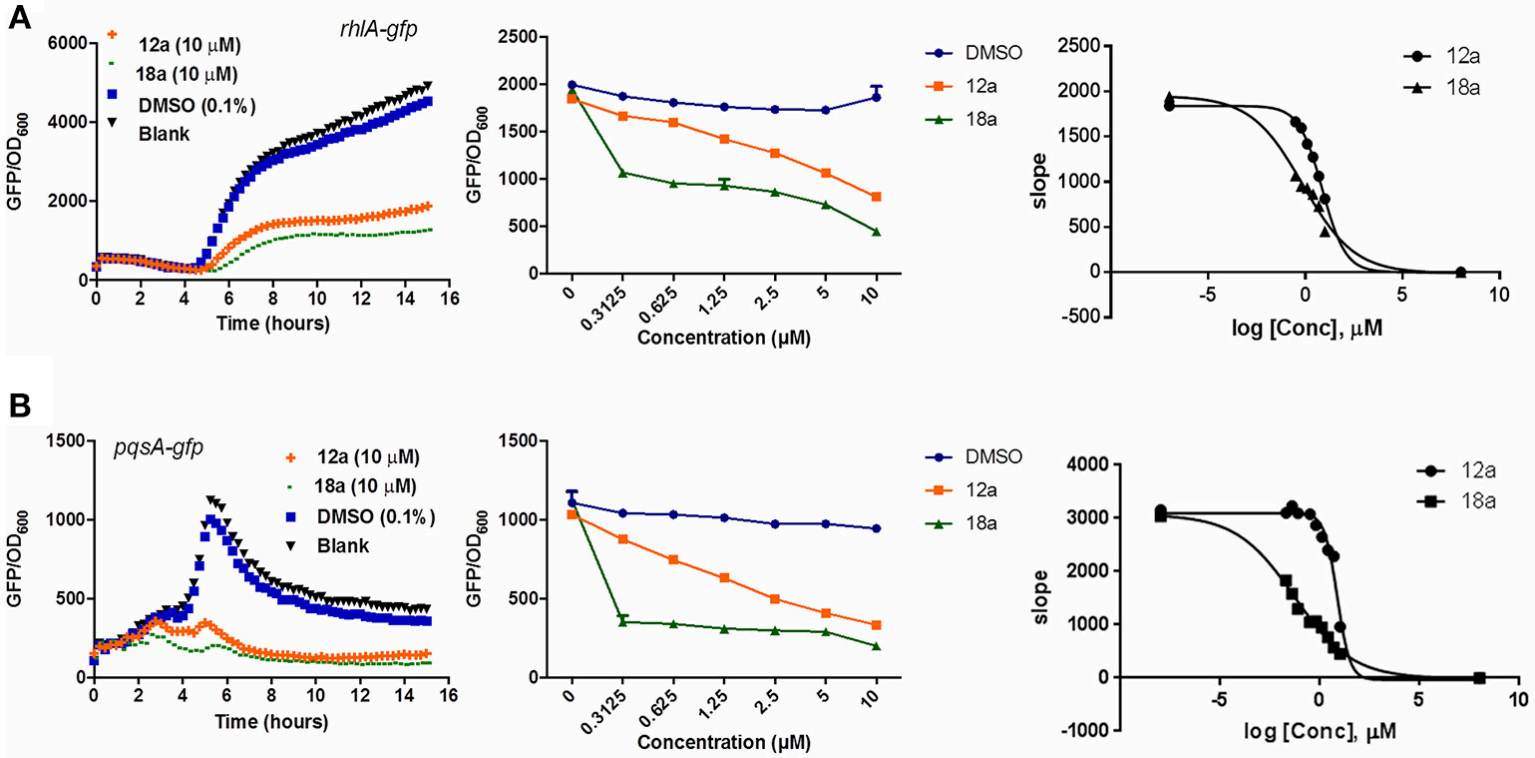
QSI inhibition, dose-response and IC_50_ values of compounds **12a** and **18a** on *P. aeruginosa rhlA-gfp* **(A)** and *pqsA-gfp* **(B)**. DMSO 0.1% was used as solvent control. For the IC_50_ values, calculation was done using GraphPad Prism 6 software, taken at time point between 4 and 6 h when QS inhibition started to occur. All experiments were done in triplicate manner (technical and biological replicates), only representative data are shown.

**Figure 3:**
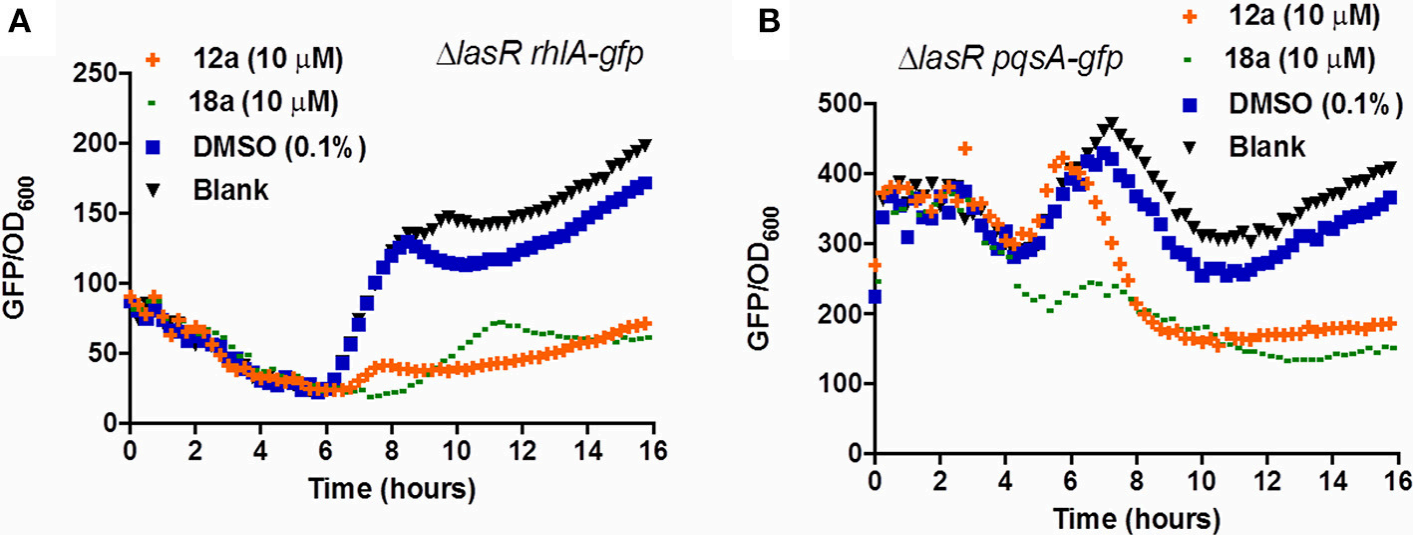
QSI inhibition of compounds **12a** and **18a** on PAO1 Δ*lasR rhlA-gfp* **(A)** and *pqsA-gfp* **(B)**. DMSO 0.1% was used as solvent control. All experiments were done in triplicate manner (technical and biological replicates), only representative data are shown.

Compounds **12a** and **18a** were found to inhibit expression of *rhlA-gfp* and *pqsA-gfp* in PAO1 WT. IC_50_ values calculated for **12a** are 6.67 ± 0.27 μM for *rhlA-gfp* and 2.51 ± 0.19 μM for *pqsA-gfp*, whereby compound **18a** provides IC_50_ values of 0.61 ± 0.04 μM for *rhlA-gfp* and 0.143 ± 0.13 for *pqsA-gfp* (Figure 2). At 10 μM, compound **12a** was observed to inhibit *rhlA-gfp* more strongly in Δ*lasR* (78% inhibition) as compared to the wild-type (58% inhibition). In Δ*lasR pqsA-gfp* reporter strain, the inhibition only happened in the later stage. Meanwhile, compound **18a** was found to inhibit both *rhlA-gfp* and *pqsA-gfp* efficiently in Δ*lasR* (Figure 3). Therefore, it is likely that two compounds have different mechanisms, and compound **18a** affect multiple QS pathways in *las-*independent pathway.

### Effects of Itaconimides on Virulence Production

**Figure 4:**
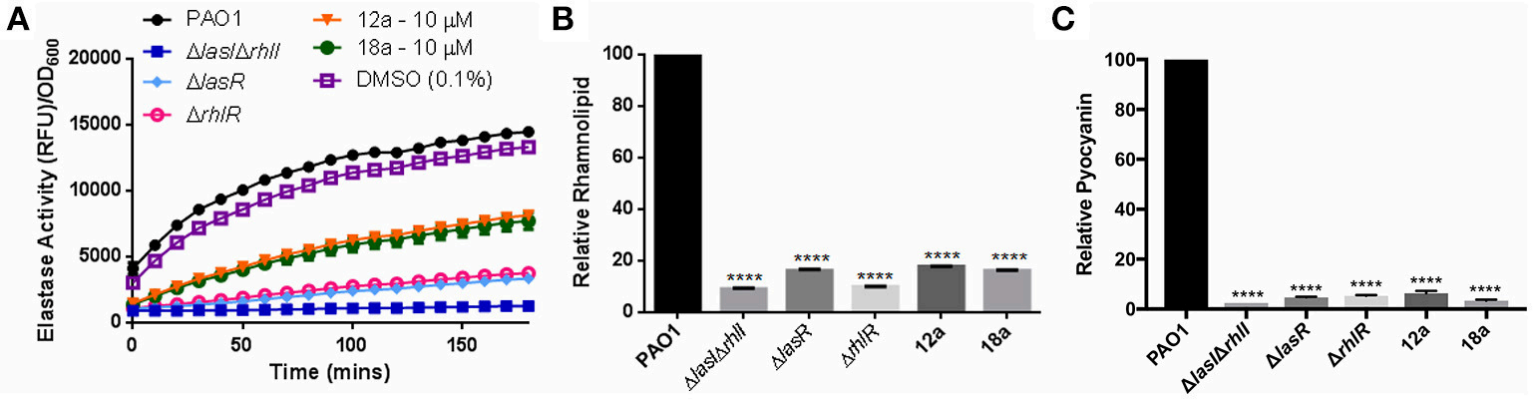
Effects of compounds **12a** and **18a** on elastase **(A)**, rhamnolipid **(B)**, and pyocyanin production **(C)**. Compounds were tested at final concentration of 10 μM. All experiments were done in triplicate manner (technical and biological replicates). Error bars are means ± SDs. ^****^p < 0.0001, Student's *t*-test.

Next, we investigated the effects of synthesized compounds on virulence factors produced by *P. aeruginosa*, notably elastase, rhamnolipid, and pyocyanin. The three virulence factors are under QS-regulation, therefore they could be a good indicator for evaluating antivirulence activities of our compounds. We also included QS mutants as control strains, where they are defective in producing quorum sensing signals hence lower level of virulence production. At 10 μM, both compounds were able to reduce all three virulence factors production. Elastase level was reduced almost half, and rhamnolipid and pyocyanin productions were abolished almost to the same level as mutant strains (Figure 4). The results showed that both compounds could indeed lower the production of virulence factors.

### Effects of Itaconimides on *P. aeruginosa* Biofilms

QS has been shown to play important roles in biofilms matrix formation (Davey et al., 2003; Sakuragi and Kolter, 2007). Mutants lacking QS often form flat and undifferentiated biofilms that could be easily cleared by antimicrobial agents and host immune system (Wu et al., 2001; Christensen et al., 2007). Here, we investigated if the itaconimides could eradicate biofilms formation when used in combination with tobramycin antibiotic.

**Figure 5:**
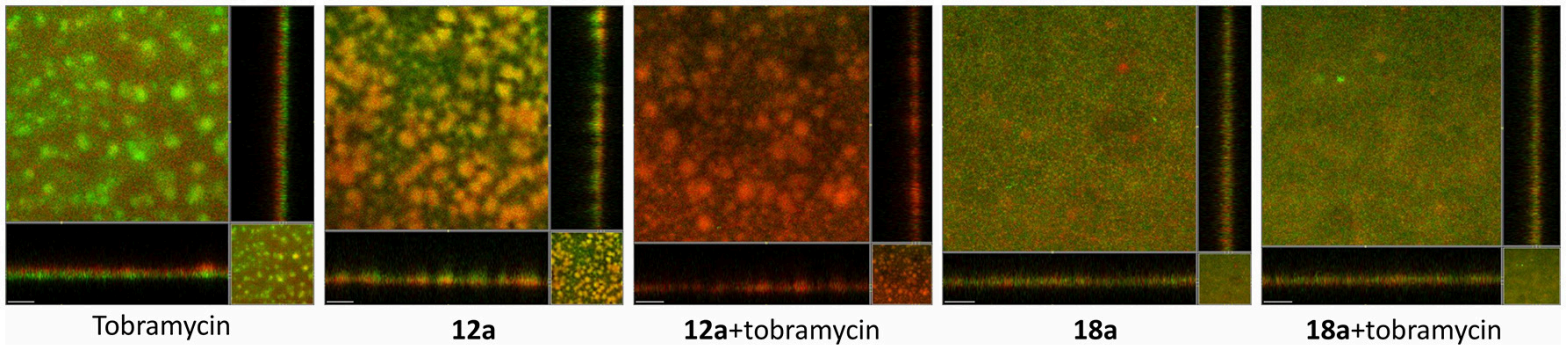
*P. aeruginosa* biofilms formed on flow cells for 72 h, followed by treatment with medium containing antibiotic (tobramycin 10 μg/ml) and compounds (10 μM) for further 48 h. Live cells are *P. aeruginosa* tagged with GFP which appeared as green, and dead cells appeared as red. Scale bars, 50 μm. Experiments were done in triplicate manner (technical and biological replicates), only representative images were shown.

From Figure 5, it can be seen that treatment with tobramycin alone (10 μg/ml) couldn't clear the whole population of *P. aeruginosa* biofilms. The dead cells only appeared on the surface of the biofilms, which indicated that the antibiotic could not penetrate into the biofilms. In addition, biofilms often contain extracellular DNA (eDNA) which could chelate aminoglycoside antibiotics and render it inactive (Chiang et al., 2013). At 10 μM, itaconimide **12a** could remarkably kill the base population of the biofilms, but not the surface. When added together with tobramycin, the combination treatment successfully eradicated the whole population of biofilms, which appeared red. Compound **18a** did not show any synergistic effect when used together with tobramycin, which could be due to poor solubility in the aqueous media.

### Cytotoxic Effects of Itaconimides on Macrophages

**Figure 6:**
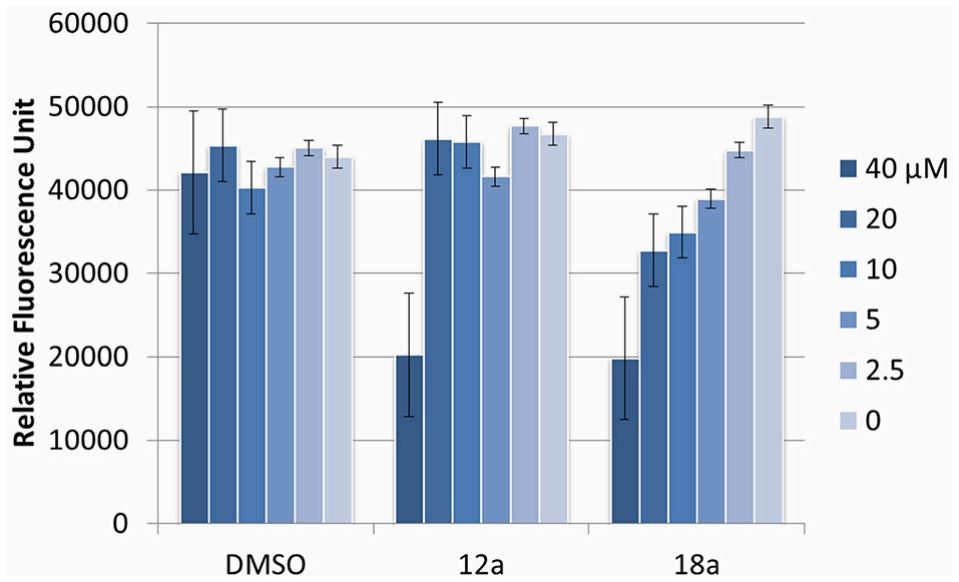
Cytotoxic effect of compounds **12a** and **18a** on murine macrophages. DMSO was used as vehicle control. Compounds were tested at various concentrations (40 μM and subsequent 2x dilution). Experiments were done in triplicate manner (technical and biological replicates). Error bars are means ± SDs.

We next evaluated the potential application of compounds **12a** and **18a** for therapeutic application. To test the cytotoxic effects, both compounds were added into murine macrophage RAW2647 cell lines, and the cell viability was measured after 16 h incubation time. Both compounds were observed to be toxic at 40 μM, but not cytotoxic at their working concentration (10 μM and lower, Figure 6). This indicates that they could be further used for subsequent *in vivo* experiments at their relevant concentration.

## Discussion

In this study, we present the investigation of itaconimides as a novel QSI against *P. aeruginosa*. The approach of using QSI molecules to attenuate the virulence of pathogenic bacteria has advantages over conventional antibiotics therapy. Instead of targeting the growth of bacteria (bactericidal or bacteriostatic strategies), QSI compounds pose lesser selective pressure for the development of resistant mutants. In several studies, it has been shown that mice treated with QSI afford better survival profile and lower bacteria count loads when compared to the control group (Hentzer et al., 2002, 2003; Jakobsen et al., 2013; Fong et al., 2017). With limited options of treatment available, antivirulence therapy could be a viable approach to mitigate the future crisis of antibiotic resistance.

In this study, a structure-activity relationship (SAR) study was performed to investigate the QSI activities of itaconimides and citraconimide analogs. We screened the compounds against QS reporter strain *lasB-gfp*, where gene expression is under control of the QS *las* system. We first investigated the inhibitory properties of the two isomers with respect to *lasB-gfp* expression. Interestingly, the itaconimide scaffold was found to be more active than the citaconimide. Next, we investigated the influence of electron donating groups (EDGs) and electron withdrawing groups (EWGs) on the phenyl group. Whereas, *p*-anisidine moiety failed to improve activity, the more electron-withdrawing 4-chloro derivative induced a 1.4-fold inhibition of PAO1-*lasB*-*gfp*. This guided us to explore anilines with different EWGs. The most potent compound in this series was **12a** from 4-bromoaniline that results in a 4.4-fold increase over the parent aniline analog. To our surprise, the 4-fluoroaniline analog **13a** did not afford a more potent inhibitor. Alongside the findings that 4-bromoaniline provided a more potent analog, we also found that the octylamine analog **14a** was more active than our hit compound **1a**. Lastly, we also included aliphatic chains to mimic the long alkyl chain of C4-HSL and 3-oxo-C12-HSL. On this note, a second series was synthesized from commercially available aliphatic amines with variating carbon lengths (*n* = 4, 6, 8, 10, 12, 14). A clear trend was observed, where longer alkyl chain resulted in lower IC_50_ values. The outcome was quite interesting, as IC_50_ values of the citraconimide substituents of these analogs (**16b**, **17b**, and **18b**) were significantly lowered. The most potent compound was found to be the tetradecylamine analog, **18a**, being the most active of both series. By substituting the naphthalene group with either 4-bromoaniline or long chain alkyl amines, IC_50_ values against PAO1-*lasB-gfp* were significantly reduced to the low micromolar range (2.41 ± 0.99 μM for **12a** and 0.17 ± 0.04 μM for **18a**). In summary, our SAR studies revealed two structurally important variations for the itaconimides that are highly important for QSI activity (Table 3).

**Table 3:** Summary of IC_50_ values of itaconimide (**a**) and citraconimide (**b**) analogs against PAO1-*lasB-gfp*.

**Compound**	**a**	**b**
**1**	7.37 ± 0.71	–
**2**	23.52 ± 0.81	–
**3**	10.27 ± 0.05	–
**4**	–	–
**5**	17.95 ± 0.49	–
**6**	10.67 ± 0.49	–
**7**	–	NA
**8**	7.45 ± 0.65	–
**9**	7.65 ± 0.37	–
**10**	6.53 ± 0.61	–
**11**	17.68 ± 0.16	–
**12**	2.41 ± 0.99	–
**13**	11.89 ± 0.34	–
**14**	26.66 ± 0.98	–
**15**	1.71 ± 0.34	–
**16**	0.30 ± 0.05	2.01 ± 0.39
**17**	0.30 ± 0.17	0.91 ± 0.20
**18**	0.17 ± 0.04	0.53 ± 0.12

*All IC_50_ values were reported as μM. Experiments were done in triplicate manner and repeated at least twice to confirm the results. NA, Not available*.

Previous studies have emphasized the advantage of therapeutics that suppress multiple QS pathways (Fong et al., 2018). Indeed, *lasR* mutants are commonly found in patients suffering from cystic fibrosis and other clinical setting (Hamood et al., 1996; Cabrol et al., 2003; Marvig et al., 2014). Nevertheless, the loss-of-function *lasR* mutants continuously express virulence traits so *P. aeruginosa* could use other pathways to bypass LasR in controlling pathogenicity (Dekimpe and Déziel, 2009; Lee et al., 2013). Using different QS reporter strains in both PAO1 WT and Δ*lasR*, it can be deduced that our compounds do not specifically inhibit one QS pathway. Both compounds could inhibit expression of *rhlA-gfp* and *pqsA-gfp* in *lasR* mutant. The inhibition of *pqsA-gfp* in Δ*lasR* was less apparent for compound **12a**, which could indicate that the compound inhibits PQS system through *las-*dependent manner. The itaconimide analogs presented here hold unique potential as broad target QSIs to control infections via an anti-virulence strategy. Future work will aim to elucidate the mechanism on how both compounds inhibit QS in *P. aeruginosa*.

Next, we also tested the effects of our compounds on the production of various virulence factors, such as elastase, rhamnolipid, and pyocyanin production. Elastase is one of the major proteases produced by *P. aeruginosa*, involved in host tissue damage and host immune responses (Kamath et al., 2002). Rhamnolipid is also an essential virulence factor and plays several key roles in biofilms formation, swarming, and in particular host immune evasion. It promotes rapid necrotic killing of polymorphonuclear (PMNs) leukocytes and also infiltration of respiratory epithelial cells (Zulianello et al., 2006; Jensen et al., 2007). *P. aeruginosa* also secretes pyocyanin, blue redox-active secondary metabolite that has several deleterious effects on mammalian cells (Lau et al., 2004), which is also regulated by QS under *pqs* system. When tested at 10 μM, the compounds could reduce production of various virulence factors controlled by QS. This shows potential of our compounds as antivirulence agent of *P. aeruginosa*.

*P. aeruginosa* biofilms are highly resistant to most antibiotics, including the last-resort polymyxin antibiotic available, colistin (Chua et al., 2016). As QS has definite role in biofilms development, it has been proposed that QSI compounds could be used as prophylactic treatment for biofilms infections. One such case is using azithromycin, which inhibits QS and biofilm formation at sub-MIC concentration. Promising results were observed in pulmonary infections and CF patients in many clinical trials data upon treatment of low-dose of AZM (2 μg/ml) (Hansen et al., 2005; Fleet et al., 2013). In this study, we utilized a combination of our compounds with the aminoglycoside tobramycin to treat *P. aeruginosa* biofilms grown on flow chambers for 3 days. We chose to study tobramycin because of its clinical relevance to cystic fibrosis (CF) patients. The clinical isolates from lung patients often confer resistance to aminoglycoside antibiotics (Hurley et al., 1995; Saiman et al., 1996). Our data also shows that treatment with tobramycin alone only kills the upper layer of biofilms. Combined with **12a**, we observed that the whole population of biofilms was eradicated. The results offer promising application of QSI in combination with antibiotics as a control for biofilm-associated infections.

It has been reported that stringent response, which provides rapid adaptation to environmental stresses, regulates QS network and also bacteria's survival in biofilms of *P. aeruginosa* (van Delden et al., 2001; Nguyen et al., 2011; Schafhauser et al., 2014). QS-deficient mutants also have lower catalase and superoxide dismutase activities, and therefore more sensitive to oxidative stress (Hassett et al., 1999). Through our biofilms experiment, we could observe some cells death upon treatment with compound **12a** in the base of the biofilms. This raises the possibility that our compound could also target stringent response and other stress-response genes, which resulted in the observed killing effect. For compound **18a**, it contains long alkyl chain, which may render its solubility and penetration into the biofilms matrix. Future study will investigate how our compounds could synergize with other antibiotics to treat biofilms from other clinical isolates.

Lastly, we investigated the cytotoxic profile of our compounds on macrophages. The cytotoxicity of the compounds is important if they are to be used in animal studies for subsequent drug development. Results indicated that compound **12a** and **18a** are not toxic up to 40 μM concentration. Still, further studies are needed to qualify the efficacy of the compounds in mice models for their potential as anti-biofilm agents, as well as their pharmacodynamic and pharmacokinetic profiles.

In conclusion, we report the novel use of itaconimides as antivirulence compounds for *P. aeruginosa*. These compounds suppress the *las, rhl*, and *pqs* QS systems of *P. aeruginosa*, and effectively abolish virulence expression activities. Compounds **12a** and **18a** showed low micromolar IC_50_ values against all three QS reporter strains with only little toxicity against macrophages at the administrated concentration. Moreover, a synergistic effect with tobramycin was observed for the killing of *P. aeruginosa* biofilms, including otherwise the tolerant and hard to target sub-population cells. Overall, our findings point to a new class of hit compounds of relevance to the development of new drugs against the superbug *P. aeruginosa*.

## Author Contributions

LY, TN, and MG designed methods and experiments. JF and KM performed the experiments and analyzed the data. AN, KQ, CT, and TN were in charge of the chemical synthesis. JF and KM wrote the paper and carefully revised by LY, TN, and MG. All authors have contributed to read and approved the manuscript.

### Conflict of Interest Statement

The authors declare that the research was conducted in the absence of any commercial or financial relationships that could be construed as a potential conflict of interest.
