# Supplementary material for: Itaconimides as Novel Quorum Sensing Inhibitors of Pseudomonas aeruginosa
Source: Front Cell Infect Microbiol. 2019 Jan 7;8:443. doi: 10.3389/fcimb.2018.00443 (PMC6330316; doi:10.3389/fcimb.2018.00443)
Supplement: Supplementary file 1 [file Data_Sheet_1.PDF]

Supplementary Material

Itaconimides as Novel Quorum Sensing Inhibitors of *Pseudomonas aeruginosa*

July Fong[1], Kim T. Mortensen [2], Amalie Nørskov[2], Katrine Qvortrup[2], Liang Yang[1,3*], Choon Hong Tan[1,4], Thomas E. Nielsen[1,5], and Michael Givskov[1,5*]

*** Correspondence:** Yang Liang: yangliang@ntu.edu.sg; Michael Givskov: mgivskov@sund.ku.dk

1 Growth curve of compounds 12a and 18a

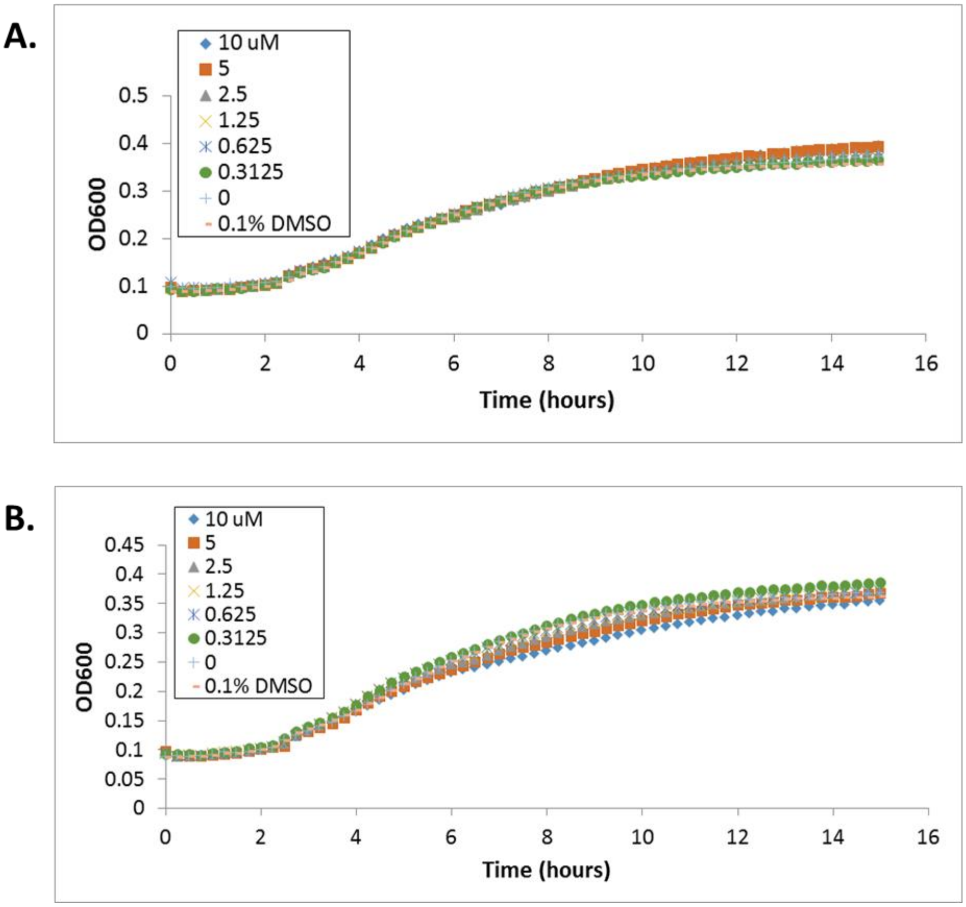

Supplementary Figure 1. Representative growth curves of *P. aeruginosa* incubated with synthesized itaconimides **12a** (A) and **18a** (B) at different concentrations. Both compounds didn't show any growth inhibition effect on *P.aeruginosa*. DMSO 0.1% was used as solvent control. Experiments were done in triplicate manner.

2 Growth curve of synthesized compounds

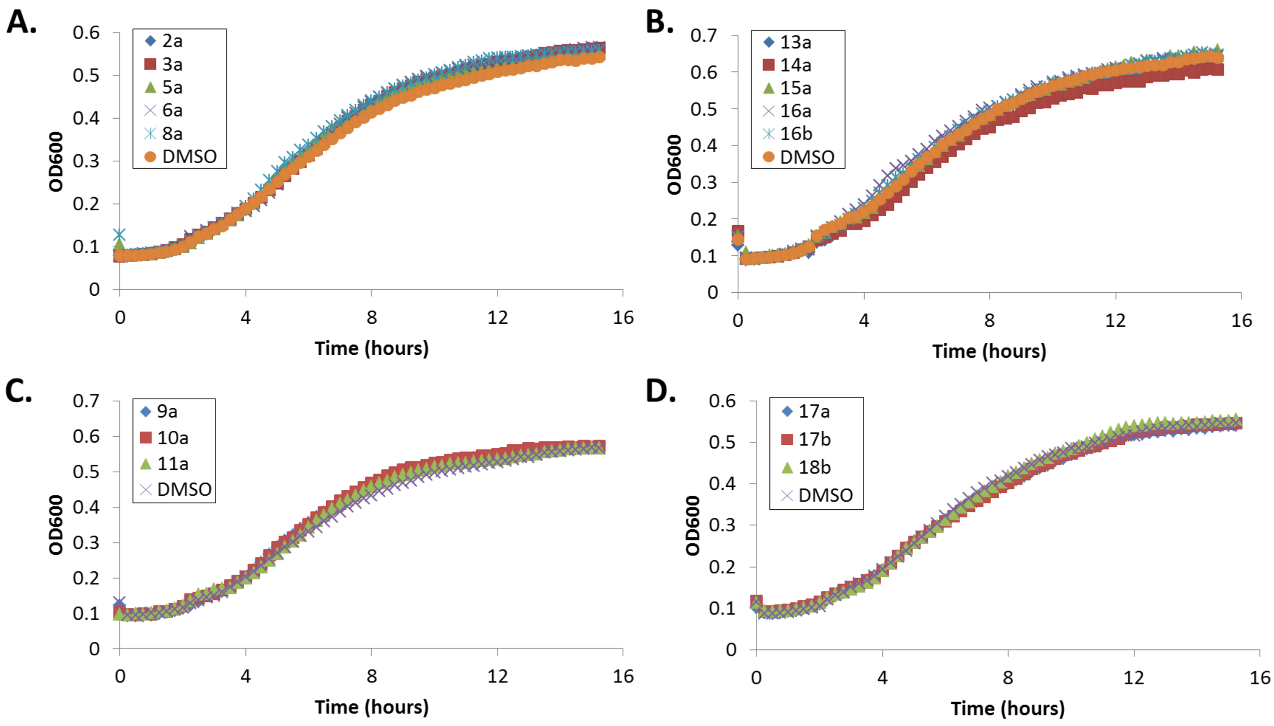

Supplementary Figure 2. Representative growth curves of *P. aeruginosa* incubated with synthesized itaconimides at working concentration (10 µM for **2a** – **14a**, 2 µM for **15a** – **18b**). The compounds didn't show any growth inhibition effect on *P.aeruginosa*. DMSO 0.1% was used as solvent control. Experiments were done in triplicate manner.

3 Fluorescence inhibition test

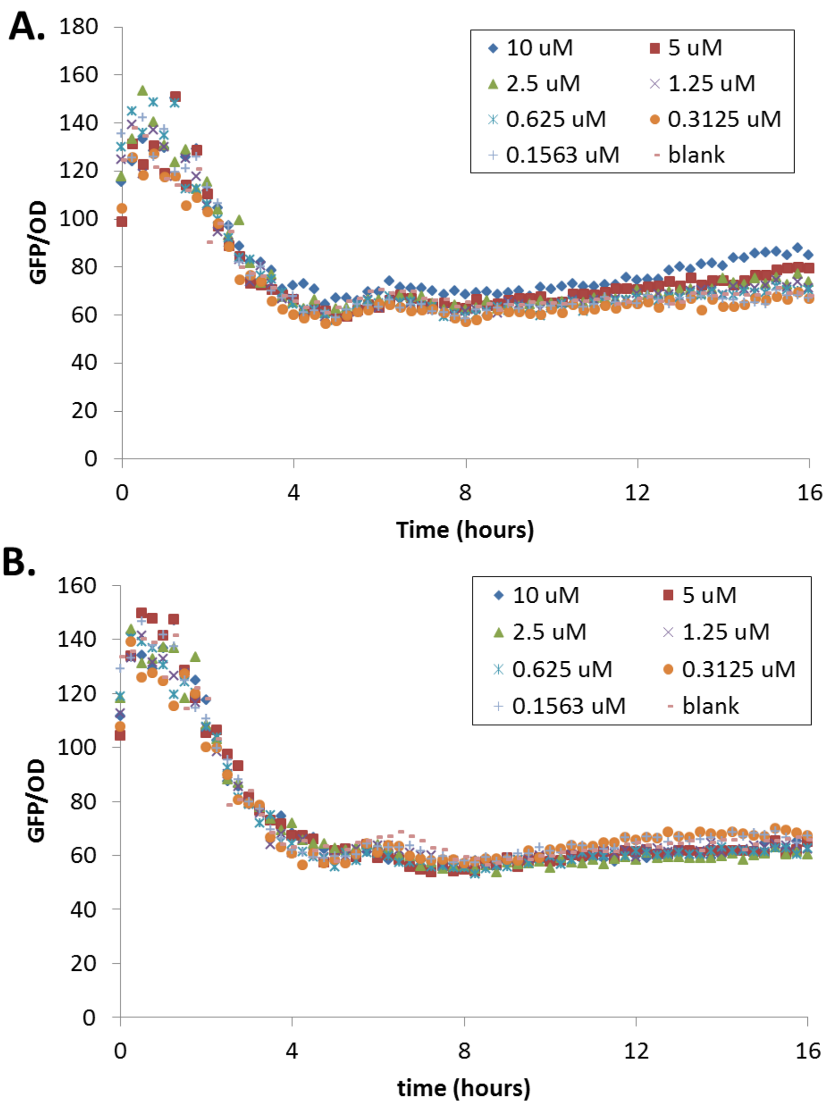

Supplementary Figure 3. Fluorescence inhibition test of itaconimides **12a** (A) and **18a** (B) on PAO1-*gfp*. Both compounds didn't show any GFP inhibition and autofluorescence property. DMSO 0.1% was used as solvent control. Experiments were done in triplicate manner. Fluorescence intensity was measured using Tecan Infinite 200 Pro plate reader (Tecan Group Ltd, Männedorf, Switzerland) with excitation wavelength at 485 nm and emission wavelength at 535 nm.

4 Chemical synthesis, ^{1}H and ^{13}C NMR spectra of synthesized compounds

4.1 General information

NMR spectra were recorded on a BrukerAvance III-400 spectrometer operating at 400 MHz for ^{1}H-NMR and 100 MHz for ^{13}C-NMR. The specific deuterated solvent is stated for each compound. Chemical shifts (δ) are given in ppm and the coupling constants (*J*) in Hz. Mass spectrometric data were recorded on a ThermoFinnigan PolarisQ MS/ThermoFinnigan LCQ Fleet MS. The values were reported in the unit of mass to charge ratio (m/z). For TLC, Merck C-60 F_{254} silica gel plates were used and developed using UV-light or a suitable stain. Flash chromatography was performed using a glass column packed with Merck 60 silica gel (40-63 µm particles) as stationary phase. All liquid phases are specified in experimental procedures. Melting points were measured using a Stuart SMP30 melting point apparatus.

4.2 General procedure for the synthesis of the itaconimides

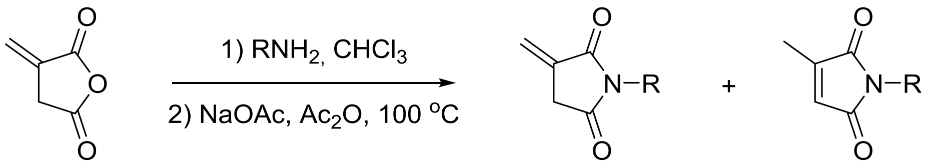

In a round-bottomed flask containing a magnetic stirring bar, the primary amine was slowly added to a vigorously stirring solution of itaconic anhydride (1.0 equiv.) in $CHCl_3$ (0.1-0.2 M) and stirred for 1 h. In the cases precipitation were observed, the carboxylic amide intermediate was collected by filtration and washed with a minimum of cold $CHCl_3$. If precipitation was not observed, all volatiles were removed under reduced pressure, and the residues were used without further purification. In a round-bottomed flask equipped with a plastic stopper, Ac_2O (3.5 equiv.) and NaOAc (0.5 equiv) was added to the intermediate and stirred for 45 min at 100 °C. The dark solution was cooled to rt and poured into ice cold H_2O and extracted with EtOAc (3 x). The combined organic phases were dried over Na_2SO_4, filtered, and concentrated *in vacuo*. The crude residues were purified immediately by flash chromatography. During the imide-formation isomerization occurred, and for some of the reactions it was possible to isolate this compound.

3-Methylene-1-(naphthalen-1-yl)pyrrolidine-2,5-dione (1a) and 3-methyl-1-(naphthalen-1-yl)-1H-pyrrole-2,5-dione (1b). According to the general procedure for synthesis of imides, 2-naphthylamine (639 mg, 4.46 mmol, 1.0 equiv.) and itaconic anhydride (500 mg, 4.46 mmol, 1.0 equiv.) in $CHCl_3$ (42.8 mL) afforded a pale pink solid that was treated with Ac_2O (1.48 mL, 15.6 mmol, 3.5 equiv.) and NaOAc (146 mg, 1.78 mmol, 0.4 equiv.) and heated at 100 °C. The dark reaction mixture was cooled and poured into ice cold H_2O (25 mL) and extracted with EtOAc (3 x 25 mL). Purified by flash chromatography (EtOAc:hexane 1:4).

3-Methylene-1-(naphthalen-1-yl)pyrrolidine-2,5-dione (1a)

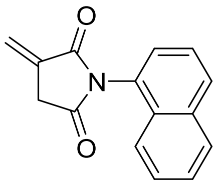

Pale yellow solid, 50% yield; mp. = 121-123 °C; R_f = 0.11 (EtOAc:hexane 1:4; UV); IR (neat) ν (cm^{-1}): 3069, 3051, 2969, 2921, 1710, 1662, 1597, 1468; ^{1}H-NMR (400 MHz, $CDCl_3$): δ 7.99-7.92 (m, 2H, Ar**H**), 7.60-7.50 (m, 4H, Ar**H**), 7.38

(dd, *J* = 7.3, 1.0 Hz, 1H, Ar**H**), 6.53 (t, *J* = 2.3 Hz, 1H, CCH**H**), 5.81 (t, *J* = 2.3 Hz, 1H, CC**H**H), 3.74-3.59 (m, 2H, C(O)C**H₂**C(CH₂C(O)); ^{13}C-NMR (101 MHz, $CDCl_3$): δ 173.3, 169.0, 134.6, 133.3, 130.3, 129.4, 128.8, 128.7, 127.4, 126.7, 126.4, 125.5, 122.2, 122.1, 34.4; MS (ESI, *m/z)* cald. for $C_{16}H_{16}NO_3^+$ $[M + CH_3OH + H]^+$ = 270.1, found $[M + CH_3OH + H]^+$ = 270.0.

3-Methyl-1-(naphthalen-1-yl)-1*H*-pyrrole-2,5-dione (1b)

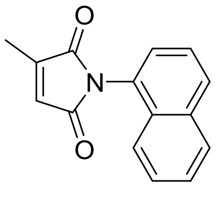

Yellow solid, 14% yield; mp. = 138-139 °C; R_f = 0.16 (EtOAc:hexane 1:4; UV); IR (neat) ν (cm^{-1}): 3091, 3059, 3013, 2960, 1702, 1637, 1597, 1465, 1374; ^{1}H-NMR (400 MHz, $CDCl_3$): δ 7.96-7.91 (m, 2H, Ar**H**), 7.57-7.50 (m, 4H, Ar**H**), 7.36 (dd, *J* = 7.3, 1.3 Hz, 1H, Ar**H**), 6.60 (q, *J* = 1.6 Hz, 1H, C(O)C**H**C(CH_3)C(O)), 2.25 (d, *J* = 1.6 Hz, 3H, C(O)CHC(C**H₃**)C(O)); ^{13}C-NMR (101 MHz, $CDCl_3$): δ 171.3, 170.2, 146.3, 134.6, 130.6, 129.9, 128.7, 128.2, 127.9, 127.2, 127.0, 126.7, 125.5, 122.5, 11.5; MS (ESI, *m/z)* cald. for $C_{15}H_{12}NO_2^+$ $[M + H]^+$ = 238.1, found $[M + H]^+$ = 238.2.

1-Mesityl-3-methylenepyrrolidine-2,5-dione (2a)

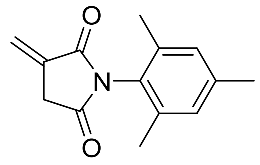

According to the general procedure for synthesis of imides, 2,4,6-trimethylaniline (376 µL, 2.68 mmol, 1.0 equiv.) and itaconic anhydride (300 mg, 2.68 mmol, 1.0 equiv.) in $CHCl_3$ (12.8 mL) afforded a white solid that was treated with Ac_2O (886 µL, 9.37 mmol, 3.5 equiv.) and NaOAc (110 mg, 1.34 mmol, 0.5 equiv.) and heated at 100 °C. The dark reaction mixture was cooled and poured into ice cold H_2O (25 mL) and extracted with EtOAc (3 x 25 mL). The residues were purified by flash chromatography (EtOAc:hexane 1:3) to afford **2a.** Light brown solid, 37% yield, mp. = 128-129 °C. R_f = 0.19 (EtOAc:hexane 1:3; UV); IR (neat) ν (cm^{-1}): 3095, 2984, 2952, 2919, 2858, 1707, 1660, 1485, 1371; ^{1}H-NMR (400 MHz, $CDCl_3$): δ 6.98 (s, 2H, Ar**H**), 6.47 (t, *J* = 2.3 Hz, 1H, CCH**H**), 5.74 (t, *J* = 2.3 Hz, 1H, CC**H**H), 3.55 (t, *J* = 2.3 Hz, 2H, C(O)C**H₂**C(CH_2)C(O)), 2.31 (s, 3H, ArC**H₃**), 2.07 (s, 6H, 2 x ArC**H₃**); ^{13}C-NMR (101 MHz, $CDCl_3$): δ 172.8, 168.5, 139.5, 135.3, 133.2, 129.4, 127.5, 121.6, 34.1, 21.1, 17.8; MS (ESI, *m/z)* cald. for $C_{14}H_{16}NO_2^+$ $[M + H]^+$ = 230.1, found $[M + H]^+$ = 230.2.

1-(3,5-Dimethylphenyl)-3-methylenepyrrolidine-2,5-dione (3a).

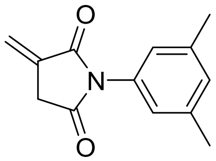

According to the general procedure for synthesis of imides, 3,5-dimethylaniline (334 µL, 2.68 mmol, 1.0 equiv.) and itaconic anhydride (301 mg, 2.68 mmol, 1.0 equiv.) in $CHCl_3$ (25.6 mL) afforded a pale brown solid that was treated with Ac_2O (886 µL, 9.37 mmol, 3.5 equiv.) and NaOAc (110 mg, 1.34 mmol, 0.5 equiv.) and heated at 100 °C. The cooled reaction mixture was poured into ice cold H_2O (25 mL) and extracted with EtOAc (3 x 25 mL). Flash chromatography (EtOAc:hexane 1:4) provided of **3a** (99 mg, 17%) as a pale orange solid; mp. = 138-140 °C; R_f = 0.17 (EtOAc:hexane 1:3; UV); IR (neat) ν (cm^{-1}): 2998, 2958, 2922, 1706, 1664, 1595, 1469, 1382; ^{1}H-NMR (400 MHz, $CDCl_3$): δ 7.04 (s, 1H, Ar**H**), 6.91 (m, 2H, Ar**H**), 6.46 (t, *J* = 2.3 Hz, 1H, CCH**H**), 5.72 (t, *J* = 2.3 Hz, 1H, CC**H**H), 3.49 (t, *J* = 2.3 Hz, 2H, C(O)C**H₂**C(CH_2)C(O)), 2.35 (s, 6H, 2 x ArC**H₃**); ^{13}C-NMR (101

MHz, $CDCl_3$): δ 173.2, 168.8, 139.2, 133.2, 131.7, 130.8, 124.3, 121.7, 34.1, 21.4; MS (ESI, *m/z)* cald. for $C_{13}H_{14}NO_2^+$ $[M + H]^+ = 216.1$, found $[M + H]^+ = 216.2$.

1-Butyl-3-methylenepyrrolidine-2,5-dione (4a)

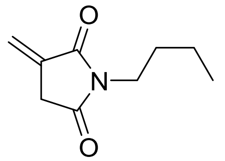

According to the general procedure for synthesis of imides, *n*-butylamine (265 µL, 2.68 mmol, 1.0 equiv.) and itaconic anhydride (301 mg, 2.68 mmol, 1.0 equiv.) in $CHCl_3$ (12.8 mL) afforded after evaporation a white residue that was treated with Ac_2O (886 µL, 9.37 mmol, 3.5 equiv.) and NaOAc (110 mg, 1.34 mmol, 0.5 equiv.) and heated at 100 °C. The dark reaction mixture was cooled and poured into ice cold H_2O (25 mL) and extracted with EtOAc (3 x 25 mL). Flash chromatography (EtOAc:hexane 1:3) provided **4a** (47 mg, 10%) as a red oil. IR (neat) ν (cm^{-1}): 2959, 2935, 2873, 1698, 1663; ^{1}H-NMR (400 MHz, $CDCl_3$): δ 6.33 (t, *J* = 2.4 Hz, 1H, CCH**H**), 5.61 (t, *J* = 2.4 Hz, 1H, CC**H**H), 3.57 (t, *J* = 7.5 Hz, 2H, NC**H₂**CH₂), 3.30 (t, *J* = 2.4 Hz, 2H, C(O)C**H₂**C(CH₂)C(O)), 1.61-1.54 (m, 2H, NCH₂C**H₂**CH₂CH₃), 1.37-1.28 (m, 2H, NCH₂CH₂C**H₂**CH₃), 0.92 (m, 3H, NCH₂CH₂CH₂C**H₃**); ^{13}C-NMR (101 MHz, $CDCl_3$): δ 174.0, 169.7, 133.5, 120.5, 38.8, 33.9, 29.9, 20.2, 13.7.

1-(2,3-Dimethylphenyl)-3-methylenepyrrolidine-2,5-dione (5a)

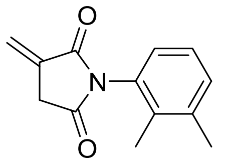

According to the general procedure for synthesis of imides, 2,3-dimethylaniline (327 µL, 2.68 mmol, 1.0 equiv.) and itaconic anhydride (300 mg, 2.68 mmol, 1.0 equiv.) in $CHCl_3$ (25.6 mL) afforded a pink solid that was treated with Ac_2O (886 µL, 9.37 mmol, 3.5 equiv.) and NaOAc (110 mg, 1.34 mmol, 0.5 equiv.) and heated at 100 °C. The dark reaction mixture was cooled and poured into ice cold H_2O (25 mL) and extracted with EtOAc (3 x 25 mL). Purification by flash column chromatography (EtOAc:hexane 1:3) gave **5a** (286 mg, 47%) as a light yellow solid. mp. = 72.5-73 °C. R_f = 0.14 (EtOAc:hexane 1:3; UV); IR (neat) ν (cm^{-1}): 3088, 3005, 2983, 2953, 2923, 1706, 1662, 1601, 1469, 1373; ^{1}H-NMR (400 MHz, $CDCl_3$): δ 7.26-7.19 (m, 2H, Ar**H**), 6.95 (d, *J* = 7.4 Hz, 1H, Ar**H**), 6.47 (t, *J* = 2.3 Hz, 1H, CCH**H**), 5.74 (t, *J* = 2.3 Hz, 1H, CC**H**H), 3.54 (m, 2H, C(O)C**H₂**C(CH₂)C(O)), 2.33 (s, 3H, ArC**H₃**), 2.03 (s, 3H, ArC**H₃**); ^{13}C-NMR (101 MHz, $CDCl_3$): δ 173.1, 168.8, 138.6, 134.3, 133.3, 131.3, 131.0, 126.5, 125.5, 121.8, 34.2, 20.5,14.5; MS (ESI, *m/z)* cald. for $C_{13}H_{14}NO_2^+$ $[M + H]^+ = 216.1$, found $[M + H]^+ = 216.2$.

3-Methylene-1-phenylpyrrolidine-2,5-dione (6a) and 3-methyl-1-phenyl-1*H*-pyrrole-2,5-dione (6b). According to the general procedure for synthesis of imides, aniline (244 µL, 2.68 mmol, 1.0 equiv.) and itaconic anhydride (301 mg, 2.68 mmol, 1.0 equiv.) in $CHCl_3$ (12.8 mL) afforded a white solid that was treated with Ac_2O (886 µL, 9.37 mmol, 3.5 equiv.) and NaOAc (110 mg, 1.34 mmol, 0.5 equiv.) and heated at 100 °C. The dark reaction mixture was cooled and poured into ice cold H_2O (25 mL) and extracted with EtOAc (3 x 25 mL). Purified by flash chromatography (EtOAc:hexane 1:3).

3-Methylene-1-phenylpyrrolidine-2,5-dione (6a)

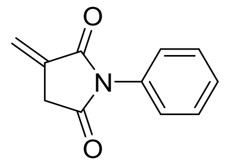

Off-white solid, 49% yield; mp. = 112°; R_f = 0.12 (EtOAc:hexane 1:3; UV); IR (neat) ν (cm^{-1}): 3096, 3069, 2992, 2957, 1701, 1661, 1593, 1496; ^{1}H-NMR (400 MHz, $CDCl_3$): δ 7.52-7.47 (m, 2H, Ar**H**), 7.43-7.39 (m, 1H, Ar**H**), 7.35-7.32 (m, 2H, Ar**H**), 6.48 (t, *J* = 2.3 Hz, 1H, CCH**H**), 5.75 (t, *J* = 2.3 Hz, 1H, CC**H**H), 3.52 (t, *J* = 2.3 Hz, 2H, C(O)C**H₂**C(CH_2)C(O)); ^{13}C-NMR (101 MHz, $CDCl_3$): δ 173.0, 168.6,133.1, 132.0, 129.3, 128.8, 126.5, 121.9, 34.1; MS (ESI, *m/z)* cald. for $C_{12}H_{14}NO_3^+$ $[M + CH_3OH + H]^+$ = 220.1, found $[M + CH_3OH + H]^+$ = 220.2.

3-Methyl-1-phenyl-1H-pyrrole-2,5-dione (6b)

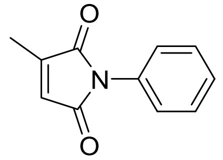

Pale yellow solid, 29% yield; mp. = 92-94; R_f = 0.29 (EtOAc:hexane 1:3; UV); IR (neat) ν (cm^{-1}): 3086, 3072, 2926, 1693, 1639, 1594, 1395; ^{1}H-NMR (400 MHz, $CDCl_3$): δ 7.48-7.44 (m, 2H, Ar**H**), 7.37-7.33 (m, 3H, Ar**H**), 6.48 (q, *J* = 1.7 Hz, 1H, C(O)C**H**C(CH_3)C(O)), 2.18 (d, *J* = 1.7 Hz, 3H, C(O)CHC(C**H₃**)C(O)); ^{13}C-NMR (101 MHz, $CDCl_3$): δ 170.8, 169.7, 145.9, 131.8, 129.2, 127.9, 127.6, 126.1, 11.3; MS (ESI, *m/z)* cald. for $C_{11}H_{10}NO_2^+$ $[M + H]^+$ = 188.1, found $[M + H]^+$ = 188.2.

1-(4-Chlorophenyl)-3-methylenepyrrolidine-2,5-dione (8a) and 1-(4-chlorophenyl)-3-methyl-1*H*-pyrrole-2,5-dione (8b). According to the general procedure for synthesis of imides, 4-chloroaniline (341 mg, 2.68 mmol, 1.0 equiv.) and itaconic anhydride (300 mg, 2.68 mmol, 1.0 equiv.) in $CHCl_3$ (12.8 mL) afforded a white solid that was treated with Ac_2O (886 μL, 9.37 mmol, 3.5 equiv.) and NaOAc (110 mg, 1.34 mmol, 0.5 equiv.) and heated at 100 °C. The dark reaction mixture was cooled and poured into ice cold H_2O (25 mL) and extracted with EtOAc (3 x 25 mL). Purified by flash chromatography (EtOAc:hexane 1:3).

1-(4-Chlorophenyl)-3-methylenepyrrolidine-2,5-dione (8a)

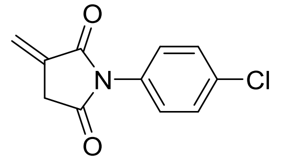

Off-white solid, 31% yield; mp. = 124-125 °C; R_f = 0.1 (EtOAc:hexane 1:3; UV); IR (neat) ν (cm^{-1}): 3095, 3063, 1705, 1665, 1493; ^{1}H-NMR (400 MHz, $CDCl_3$): δ 7.48-7.44 (m, 2H, Ar**H**), 7.33-7.30 (m, 2H, Ar**H**), 6.49 (t, *J* = 2.3 Hz, 1H, CCH**H**), 5.76 (t, *J* = 2.3 Hz, 1H, CC**H**H), 3.51 (t, *J* = 2.3 Hz, 2H, C(O)C**H₂**C(CH_2)C(O)); ^{13}C-NMR (101 MHz, $CDCl_3$): δ 172.6, 168.3, 134.6, 132.8, 130.4, 129.5, 127.7, 122.3, 34.1; MS (ESI, *m/z)* cald. for $C_{11}H_9ClNO_2^+$ $[M + H]^+$ = 222.0, found $[M + H]^+$ = 222.1.

1-(4-Chlorophenyl)-3-methyl-1*H*-pyrrole-2,5-dione (8b)

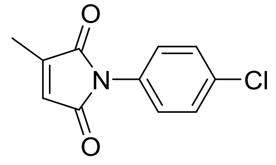

Pale yellow solid, 40% yield; mp. = 109 °C; R_f = 0.24 (EtOAc:hexane 1:3; UV); IR (neat) ν (cm^{-1}): 3083, 2928, 1696, 1644, 1495, 1400; ^{1}H-NMR (400 MHz, $CDCl_3$): δ 7.44-7.41 (m, 2H, Ar**H**), 7.33-7.30 (m, 2H, Ar**H**), 6.49 (q, *J* = 1.8 Hz, 1H, C(O)C**H**C(CH_3)C(O)), 2.17 (t, *J* = 1.8 Hz, 3H, C(O)CHC(C**H₃**)C(O)); ^{13}C-NMR (101 MHz, $CDCl_3$): δ 170.4, 169.4,146.1, 133.5, 130.3, 129.4, 127.7, 127.1, 11.3; MS (ESI, *m/z)* cald. for $C_{12}H_{13}ClNO_3^+$ $[M + CH_3OH + H]^+$ = 254.1, found $[M + CH_3OH + H]^+$ = 253.9.

1-(4-Chloro-2-fluorophenyl)-3-methylenepyrrolidine-2,5-dione (9a) and 1-(4-chloro-2-fluorophenyl)-3-methyl-1*H*-pyrrole-2,5-dione (9b). According to the general procedure for synthesis of imides, 4-chloro-2-fluoroaniline (297 μL, 2.68 mmol, 1.0 equiv.) and itaconic anhydride (300 mg, 2.68 mmol, 1.0 equiv.) in $CHCl_3$ (12.8 mL) afford a white solid that was treated with Ac_2O (886 μL, 9.37 mmol, 3.5 equiv.) and NaOAc (110 mg, 1.34 mmol, 0.5 equiv.) and heated at 100 °C. The dark reaction mixture was cooled and poured into ice cold H_2O (25 mL) and extracted with EtOAc (3 x 25 mL). Purified by flash chromatography (EtOAc:hexane 1:3).

1-(4-Chloro-2-fluorophenyl)-3-methylenepyrrolidine-2,5-dione (9a)

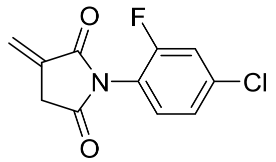

Off-white solid, 24% yield; mp. = 109-110 °C; R_f = 0.13 (EtOAc:hexane 1:3; UV); IR (neat) ν (cm^{-1}): 3119 3065, 2925, 1713, 1663, 1586, 1498; ^{1}H-NMR (400 MHz, DMSO): δ 7.29-7.20 (m, 3H, Ar**H**), 6.49 (t, *J* = 2.3 Hz, 1H, CCH**H**), 5.77 (t, *J* = 2.3 Hz, 1H, CC**H**H), 3.55 (m, 2H, C(O)C**H**$_2$C(CH$_2$)C(O)); ^{13}C-NMR (101 MHz, DMSO): δ 171.9, 167.7, 157.4 (d, J_{CF} = 256.3 Hz), 136.5 (d, J_{CF} = 10.0 Hz), 132.9, 130.15, 130.14, 125.4 (d, J_{CF} = 3.9 Hz), 122.8, 117.9 (d, J_{CF} = 23.1 Hz), 34.32; MS (ESI, *m/z)* cald. for $C_{12}H_{12}ClFNO_3^+$ $[M + CH_3OH + H]^+$ = 272.1, found $[M + CH_3OH + H]^+$ = 271.9.

1-(4-Chloro-2-fluorophenyl)-3-methyl-1*H*-pyrrole-2,5-dione (9b)

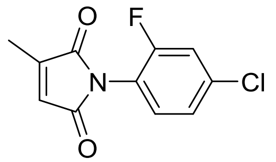

Off-white semi-solid, 14% yield; IR (neat) ν (cm^{-1}): 3102, 2993, 2957, 2923, 1709, 1641, 1585, 1500, 1388; ^{1}H-NMR (400 MHz, $CDCl_3$): δ 7.27-7.18 (m, 3H, Ar**H**), 6.52 (q, *J* = 1.8 Hz, 1H, C(O)C**H**C(CH$_3$)C(O)), 2.18 (d, *J* = 1.8 Hz, 3H, C(O)CHC(C**H$_3$**)C(O)); ^{13}C-NMR (101 MHz, $CDCl_3$): δ 169.7, 168.5, 157.7 (d, J_{CF} = 255.9 Hz), 146.7, 135.7 (d, J_{CF} = 9.3 Hz), 130.49, 130.48, 128.2, 125.2 (d, J_{CF} = 3.9 Hz), 117.7 (d, J_{CF} = 23.1 Hz),11.4.

1-(3-Chlorophenyl)-3-methylenepyrrolidine-2,5-dione (10a) and 1-(3-chlorophenyl)-3-methyl-1*H*-pyrrole-2,5-dione (10b). According to the general procedure for synthesis of imides, 3-chloroaniline (283 μL, 2.68 mmol, 1.0 equiv.) and itaconic anhydride (300 mg, 2.68 mmol, 1.0 equiv.) in $CHCl_3$ (12.8 mL) afforded a off-white solid that was treated with Ac_2O (886 μL, 9.37 mmol, 3.5 equiv.) and NaOAc (110 mg, 1.34 mmol, 0.5 equiv.) and heated at 100 °C. The dark reaction mixture was cooled and poured into ice cold H_2O (25 mL) and extracted with EtOAc (3 x 25 mL). Purified by flash chromatography (EtOAc:hexane 1:3).

1-(3-Chlorophenyl)-3-methylenepyrrolidine-2,5-dione (10a)

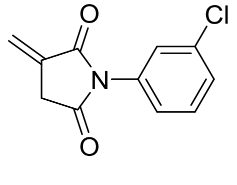

Off-white solid, 35% yield; mp. = 88-90 °C; R_f = 0.1 (EtOAc:hexane 1:3; UV); IR (neat) ν (cm^{-1}): 3119, 3079, 3011, 2922, 1706, 1659, 1593, 1477, 1381; ^{1}H-NMR (400 MHz, DMSO): δ 7.44-7.37 (m, 3H, Ar**H**), 7.28-7.26 (m, 1H, Ar**H**), 6.49 (t, *J* = 2.3 Hz, 1H, CCH**H**), 5.77 (t, J = 2.3 Hz, 1H, CC**H**H), 3.51 (t, *J* = 2.3

Hz, 2H, C(O)C**H₂**C(CH₂)C(O)); ^{13}C-NMR (101 MHz, DMSO): δ 172.5, 168.2, 134.8, 133.0, 132.7, 130.2, 129.0, 126.8, 124.7, 122.4, 34.1; MS (ESI, *m/z)* cald. for $C_{11}H_9ClNO_2^+$ $[M + H]^+$ = 222.0, found $[M + H]^+$ = 222.3.

1-(3-Chlorophenyl)-3-methyl-1*H*-pyrrole-2,5-dione (10b)

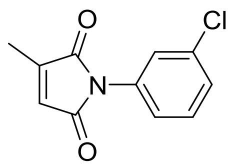

Off-white solid, 15% yield; mp. = 52.6-53.0 °C; R_f = 0.24 (EtOAc:hexane 1:3; UV); IR (neat) ν (cm^{-1}): 3093, 2963, 2925, 1706, 1642, 1594, 1478, 1388; ^{1}H-NMR (400 MHz, $CDCl_3$): δ 7.41-7.27 (m, 4H, Ar**H**), 6.49 (q, *J* = 1.8Hz, 1H, C(O)C**H**C(CH_3)C(O)), 2.18 (d, *J* = 1.8 Hz, 3H, (O)CHC(C**H₃**)C(O)); ^{13}C-NMR (101 MHz, $CDCl_3$): δ 170.3, 169.2, 146.1, 134.7, 132.9, 130.1, 127.9, 127.7, 126.0, 123.9, 11.3; MS (ESI, *m/z)* cald. for $C_{12}H_{13}ClNO_3^+$ $[M + CH_3OH + H]^+$ = 254.1, found $[M + CH_3OH + H]^+$ = 254.0.

1-(2-Chlorophenyl)-3-methylenepyrrolidine-2,5-dione (11a) and 1-(2-chlorophenyl)-3-methyl-1*H*-pyrrole-2,5-dione (11b). According to the general procedure for synthesis of imides, 2-chloroaniline (282 µL, 2.68 mmol, 1.0 equiv.) and itaconic anhydride (300 mg, 2.68 mmol, 1.0 equiv.) in $CHCl_3$ (12.8 mL) afforded a off-white solid that was treated with Ac2O (886 µL, 9.37 mmol, 3.5 equiv.) and NaOAc (110 mg, 1.34 mmol, 0.5 equiv.) and heated at 100 °C. The dark reaction mixture was cooled and poured into ice cold H_2O (25 mL) and extracted with EtOAc (3 x 25 mL). Purified by flash chromatography (EtOAc:hexane 1:3).

1-(2-Chlorophenyl)-3-methylenepyrrolidine-2,5-dione (11a)

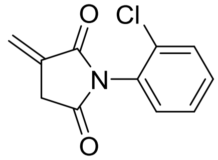

Off-white solid, 61% yield; mp. = 83.2-83.5 °C; R_f = 0.13 (EtOAc:hexane 1:3; UV); IR (neat) ν (cm^{-1}): 3101, 2960, 2932, 1710, 1662, 1483; ^{1}H-NMR (400 MHz, DMSO): δ 7.57-7.53 (m, 1H, Ar**H**), 7.44-7.37 (m, 2H, Ar**H**), 7.28-7.23 (m, 1H, Ar**H**), 6.48 (t, *J* = 2.3 Hz, 1H, CCH**H**), 5.76 (t, *J* = 2.3 Hz, 1H, CC**H**H), 3.63-3.48 (m, 2H, C(O)C**H₂**C(CH₂)C(O)); ^{13}C-NMR (101 MHz, DMSO): δ 172.3, 168.0, 133.2, 132.5, 131.0, 130.7, 130.1, 130.0,128.0, 122.4, 34.4; MS (ESI, *m/z)* cald. for $C_{12}H_{13}ClNO_3^+$ $[M + CH_3OH + H]^+$ = 254.1, found $[M + CH_3OH + H]^+$ = 254.1.

1-(2-Chlorophenyl)-3-methyl-1*H*-pyrrole-2,5-dione (11b)

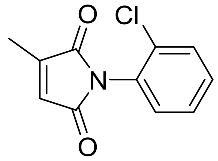

Light yellow solid, 9% yield; mp. = 91-94 °C; R_f = 0.28 (EtOAc:hexane 1:3; UV); IR (neat) ν (cm^{-1}): 3095, 3070, 3042, 1702, 1642, 1488, 1392; ^{1}H-NMR (400 MHz, DMSO): δ 7.54-7.52 (m, 1H, Ar**H**), 7.42-7.35 (m, 2H, Ar**H**), 7.27-7.24 (m, 3H, Ar**H**), 6.51 (q, *J* = 1.8 Hz, 1H, C(O)C**H**C(CH_3)C(O)), 2.19 (d, *J* = 1.8 Hz, 3H, C(O)CHC(C**H₃**)C(O)); ^{13}C-NMR (101 MHz, DMSO): δ 170.1, 169.0, 146.4, 133.3, 130.8, 130.7, 130.5, 129.6, 128.0, 127.8, 11.4; MS (ESI, *m/z)* cald. for $C_{11}H_9ClNO_2^+$ $[M + H]^+$ = 222.0, found $[M + H]^+$ = 222.2.

1-(4-Bromophenyl)-3-methylenepyrrolidine-2,5-dione (12a) and 1-(4-bromophenyl)-3-methyl-1*H*-pyrrole-2,5-dione (12b). According to the general procedure for synthesis of imides, 4-

bromoaniline (460 mg, 2.68 mmol, 1.0 equiv.) and itaconic anhydride (300 mg, 2.68 mmol, 1.0 equiv.) in $CHCl_3$ (12.8 mL) afforded a white solid that was treated with Ac2O (886 µL, 9.37 mmol, 3.5 equiv.) and NaOAc (110 mg, 1.34 mmol, 0.5 equiv.) and heated at 100 bu°C. The dark reaction mixture was cooled and poured into ice cold H2O (25 mL) and extracted with EtOAc (3 x 25 mL). Purified by flash chromatography (EtOAc:hexane 1:3).

1-(4-Bromophenyl)-3-methylenepyrrolidine-2,5-dione (12a)

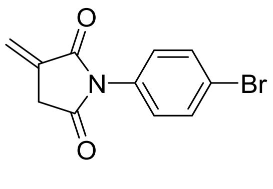

Off-white solid, 37% yield; mp. = 138-139 °C; R_f = 0.09 (EtOAc:hexane 1:3; UV); IR (neat) ν (cm^{-1}): 3093, 3062, 2994, 1705, 1665, 1490; ^{1}H-NMR (400 MHz, DMSO): δ 7.63-7.59 (m, 2H, Ar**H**), 7.27-7.23 (m, 2H, Ar**H**), 6.48 (t, *J* = 2.3 Hz, 1H, CCH**H**), 5.76 (t, *J* = 2.3 Hz, 1H, CC**H**H), 3.50 (t, *J* = 2.3 Hz, 2H, C(O)C**H₂**C(CH₂)C(O)); ^{13}C-NMR (101 MHz, DMSO): δ 172.6, 168.2, 132.8, 132.5, 130.9, 128.0, 122.6, 122.3, 34.1; MS (ESI, *m/z)* cald. for $C_{11}H_9BrNO_2^+$ $[M+H]^+$ = 266.0, found $[M+H]^+$ = 266.2.

1-(4-Bromophenyl)-3-methyl-1*H*-pyrrole-2,5-dione (12b)

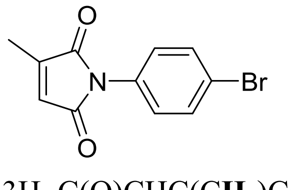

Light yellow solid, 20% yield; mp. = 116-118 °C; R_f = 0.28 (EtOAc:hexane 1:3; UV); IR (neat) ν (cm^{-1}): 3087, 2962, 2923, 2843, 1697, 1646, 1592, 1491, 1375; ^{1}H-NMR (400 MHz, DMSO): δ 7.59-7.56 (m, 2H, Ar**H**), 7.28-7.24 (m, 2H, Ar**H**), 6.48 (q, *J* = 1.8 Hz, 1H, C(O)C**H**C(CH_3)C(O)), 2.17 (d, *J* = 1.8 Hz, 3H, C(O)CHC(C**H_3**)C(O)); ^{13}C-NMR (101 MHz, DMSO): δ 170.4, 169.3, 146.1, 132.4,130.9, 127.7, 127.3, 121.4, 11.3.

1-(4-Fluorophenyl)-3-methylenepyrrolidine-2,5-dione (13a) and 1-(4-fluorophenyl)-3-methyl-1*H*-pyrrole-2,5-dione (13b). According to the general procedure for synthesis of imides, 4-fluoroaniline (254 µL, 2.68 mmol, 1.0 equiv.) and itaconic anhydride (300 mg, 2.68 mmol, 1.0 equiv.) in $CHCl_3$ (12.8 mL) afforded a white solid that was treated with Ac_2O (886 µL, 9.37 mmol, 3.5 equiv.) and NaOAc (110 mg, 1.34 mmol, 0.5 equiv.) and heated at 100 °C. The dark reaction mixture was cooled and poured into ice cold H_2O (25 mL) and extracted with EtOAc (3 x 25 mL). Purified by flash chromatography (EtOAc:hexane 1:3).

1-(4-Fluorophenyl)-3-methylenepyrrolidine-2,5-dione (13a)

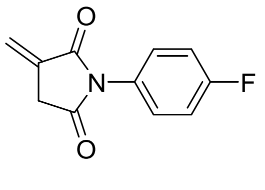

Off-white solid, 40% yield; mp. = 135-135.5 °C; R_f = 0.08 (EtOAc:hexane 1:3; UV); IR (neat) ν (cm^{-1}): 3067, 2962, 2928, 1703, 1664, 1602, 1511, 1386; ^{1}H-NMR (400 MHz, CDCl3): δ 7.36-7.31 (m, 2H, Ar**H**), 7.20-7.14 (m, 2H, Ar**H**), 6.48 (t, *J* = 2.4 Hz, 1H, CCH**H**), 5.75 (t, *J* = 2.4 Hz, 1H, CC**H**H), 3.51 (t, *J* = 2.4 Hz, 2H, C(O)C**H₂**C(CH₂)C(O)); ^{13}C-NMR (101 MHz, $CDCl_3$): δ 172.9, 168.5, 162.3 (J_{CF} = 248.6 Hz), 132.9, 128.4 (J_{CF} = 8.6 Hz), 127.9 (J_{CF} = 3.25 Hz), 122.2, 116.3 (J_{CF} = 23.0 Hz), 34.0; MS (ESI, *m/z)* cald. for $C_{12}H_{13}FNO_3^+$ $[M + CH_3OH + H]^+$ = 238.1, found $[M + CH_3OH + H]^+$ = 238.1.

1-(4-Fluorophenyl)-3-methyl-1*H*-pyrrole-2,5-dione (13b)

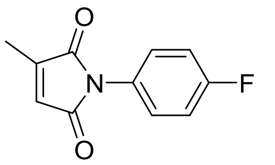

Off-white solid, 19% yield; mp. = 137 °C; R_f = 0.24 (EtOAc:hexane 1:3; UV); IR (neat) ν (cm^{-1}): 3093, 3078, 2997, 2931, 1698, 1640, 1602, 1513, 1405; ^{1}H-NMR (400 MHz, CDCl3): δ 7.34-7.29 (m, 2H, Ar**H**), 7.17-7.11 (m, 2H, Ar**H**), 6.48 (q, *J* = 1.8 Hz, 1H, C(O)C**H**C(CH_3)C(O)), 2.17 (d, *J* = 1.8 Hz, 3H, C(O)CHC(C**H_3**)C(O)), ^{13}C-NMR (101 MHz, $CDCl_3$): δ 170.7, 169.6, 161.8 (d, J_{CF} = 247.6 Hz), 146.0, 127.9 (d, J_{CF} = 8.5 Hz), 127.7 (d, J_{CF} = 3.1 Hz), 127.6, 116.2 (d, J_{CF} = 22.7 Hz), 11.3; MS (ESI, *m/z)* cald. for $C_{11}H_9FNO_2^+$ $[M + H]^+$ = 206.1, found $[M + H]^+$ = 206.1.

1-Hexyl-3-methylenepyrrolidine-2,5-dione (14a) and 1-hexyl-3-methyl-1*H*-pyrrole-2,5-dione (14b). According to the general procedure for synthesis of imides, hexylamine (1.95 mL, 8.92 mmol, 1.0 equiv.) and itaconic anhydride (1.00 g, 8.92 mmol, 1.0 equiv.) in $CHCl_3$ (42.8 mL) afforded after evaporation a white residue that was treated with Ac_2O (3.0 mL, 31.2 mmol, 3.5 equiv.) and NaOAc (366 mg, 4,46 mmol, 0.5 equiv.) and heated at 100 °C. The dark reaction mixture was cooled and poured into ice cold H2O (50 mL) and extracted with EtOAc (3 x 50 mL). Purified by flash chromatography (hexane → 1:7 EtOAc:Hexane).

1-Hexyl-3-methylenepyrrolidine-2,5-dione (14a)

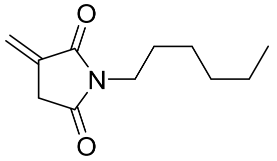

Red oil, 8% yield; R_f = 0.13 (EtOAc:hexane 1:7; UV); IR (neat) ν (cm^{-1}): 2930, 2859, 1701, 1663, 1391; ^{1}H-NMR (400 MHz, $CDCl_3$): δ 6.32 (t, *J* = 2.3 Hz, 1H, CCH**H**), 5.60 (t, J = 2.3 Hz, 1H, CC**H**H), 3.55 (t, *J* = 7.5 Hz, 2H, NC**H_2**CH_2), 3.29 (t, *J* = 2.3 Hz, 2H, C(O)C**H_2**C(CH_2)C(O)), 1.61-1.54 (m, 2H, NCH_2C**H_2**), 1.30-1.27 (m, 6H, C**H_2**C**H_2**C**H_2**), 0.86 (t, *J* = 6.8 Hz, 3H, C**H_3**); ^{13}C-NMR (101 MHz, $CDCl_3$): δ 174.0, 169.7, 133.5, 120.4, 39.0, 33.9, 31.4, 27.8, 26.6, 22.6, 14.1; MS (ESI, *m/z)* cald. for $C_{11}H_{18}NO_2^+$ [M + H]+ = 196.1, found $[M+H]^+$ = 195.8.

1-Hexyl-3-methyl-1*H*-pyrrole-2,5-dione (14b)

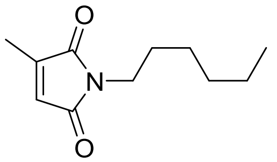

Red oil, 14% yield; R_f = 0.34 (EtOAc:Heptane (1:7); UV); IR (neat) ν (cm^{-1}): 3100, 2956, 2930, 2859, 1702, 1643, 1376; ^{1}H-NMR (400 MHz, CDCl3): δ 6.29 (q, *J* = 1.8 Hz, 1H, C(O)C**H**C(CH_3)C(O)), 3.46 (t, *J* = 7.3 Hz, 2H, NC**H_2**CH_2), 2.06 (d, *J* = 1.8 Hz, 3H, C(O)CHC(C**H_3**)C(O)), 1.58-1.51 (m, 2H, NCH_2C**H_2**), 1.28-1.24 (m, 6H, C**H_2**C**H_2**C**H_2**), 0.86 (t, *J* = 6.7 Hz, 3H, C**H_3**); ^{13}C-NMR (101 MHz, $CDCl_3$): δ 172.1, 171.1, 145.6, 127.3, 38.1, 31.4, 28.7, 26.5, 22.6, 14.1, 11.1; MS (ESI, *m/z)* cald. for $C_{12}H_{22}NO_3^+$ $[M + CH_3OH + H]^+$ = 228.2, found $[M + CH_3OH + H]^+$ = 228.1.

3-Methylene-1-octylpyrrolidine-2,5-dione (15a)

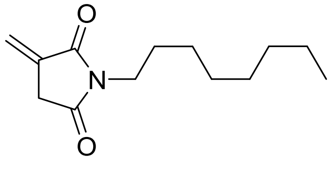

According to the general procedure for synthesis of imides, octylamine (442 mg, 2.68 mmol, 1.0 equiv.) and itaconic anhydride (300 mg, 2.68

mmol, 1.0 equiv.) in $CHCl_3$ (12.8 mL) afforded after evaporation a white residue that was treated with Ac_2O (886 \µL, 9.37 mmol, 3.5 equiv.) and NaOAc (110 mg, 1.34 mmol, 0.5 equiv.) and heated at 100 °C. The dark reaction mixture was cooled and poured into ice cold H_2O (25 mL) and extracted with EtOAc (3 x 25 mL). Purification by flash chromatography (1:3 EtOAc:Hexane) provided **15a** (98 mg, 16%) as an off-white semi-solid. R_f = 0.27 (EtOAc:hexane 1:3; UV); IR (neat) ν (cm^{-1}): 2925, 2855, 1703, 1664. 1367; ^{1}H-NMR (400 MHz, $CDCl_3$): δ 6.33 (t, *J* = 2.3 Hz, 1H, CCH**H**), 5.61 (t, *J* = 2.3 Hz, 1H, CC**H**H), 3.56 (t, *J* = 7.5 Hz, 2H, NC**H₂**CH₂), 3.30 (t, *J* = 2.3 Hz, 2H, C(O)C**H₂**C(CH₂)C(O)), 1.59 (p, *J* = 7.3 Hz, 2H, NCH₂C**H₂**), 1.29-1.25 (m, 10H, C **H₂**C **H₂**C**H₂**C**H₂**C**H₂**), 0.87 (t, *J* = 6.7 Hz, 3H, CH₂C**H₃**); ^{13}C-NMR (101 MHz, $CDCl_3$): δ 174.0, 169.7, 133.5, 120.5, 39.1, 33.9, 31.9, 29.3, 27.9, 27.0, 22.7, 14.2; MS (ESI, *m/z)* cald. for $C_{13}H_{22}NO_2^+$ $[M + H]^+$ = 224.2, found $[M + H]^+$ = 224.2.

1-Decyl-3-methylenepyrrolidine-2,5-dione (16a) and 1-decyl-3-methyl-1*H*-pyrrole-2,5-dione (16b). According to the general procedure for synthesis of imides, decylamine (1.78 mL, 8.92 mmol, 1.0 equiv.) and itaconic anhydride (1.00 g, 8.92 mmol, 1.0 equiv.) in $CHCl_3$ (42.8 mL) afforded after evaporation a white residue that was treated with Ac_2O (2.95 mL, 31.2 mmol, 3.5 equiv.) and NaOAc (366 mg, 4,46 mmol, 0.5 equiv.) and heated at 100 °C. The dark reaction mixture was cooled and poured into ice cold H_2O (50 mL) and extracted with EtOAc (3 x 50 mL). Purified by flash chromatography (hexane → EtOAc:hexane 1:9).

1-Decyl-3-methylenepyrrolidine-2,5-dione (16a)

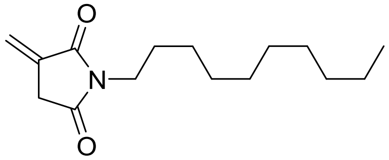

Red oil, 13% yield; R_f = 0.11 (EtOAc:hexane 1:9; UV); IR (neat) ν (cm^{-1}): 3093, 2913, 2847, 1697, 1662, 1390; ^{1}H-NMR (400 MHz, $CDCl_3$): δ 6.33 (t, *J* = 2.5 Hz, 1H, CCH**H**), 5.60 (t, *J* = 2.0 Hz, 1H, CC**H**H), 3.56 (t, *J* = 7.5 Hz, 2H, NC**H₂**CH₂), 3.30 (t, *J* = 2.2 Hz, 2H, C(O)C**H₂**C(CH₂)C(O)), 1.62-1.55 (m, 2H, NCH₂C**H₂**), 1.28-1.24 (m, 14H, C**H₂**C**H₂**C**H₂**C**H₂**C**H₂**C**H₂**C**H₂**), 0.87 (t, *J* = 6.9 Hz, 3H, CH₂C**H₃**); ^{13}C-NMR (101 MHz, $CDCl_3$): δ 174.0, 169.7, 133.5, 120.4, 39.1, 33.9, 32.0, 29.63, 29.59, 29.4, 29.3, 27.9, 27.0, 22.8, 14.2; MS (ESI, *m/z)* cald. for $C_{16}H_{30}NO_3^+$ $[M + CH_3OH + H]^+$ = 284.2, found $[M + CH_3OH + H]^+$ = 284.3.

1-Decyl-3-methyl-1*H*-pyrrole-2,5-dione (16b)

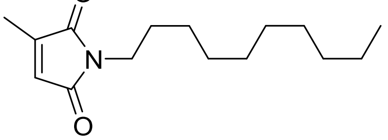

Pink semi-solid, 10% yield; R_f = 0.30 (EtOAc:hexane 1:9; UV); IR (neat) ν (cm^{-1}): 2923, 2854, 1704, 1643, 1375; ^{1}H-NMR (400 MHz, $CDCl_3$): δ 6.29 (q, *J* = 1.8 Hz, 1H, C(O)CHC(CH₃)C(O)), 3.47 (t, *J* = 7.4 Hz, 2H, NCH₂CH₂), 2.06 (d, *J* = 1.8 Hz, 3H, C(O)CHC(CH₃)C(O)), 1.59-1.52 (m, 2H, NCH₂CH₂), 1.27-1.24 (m, 14H, CH₂CH₂CH₂CH₂CH₂CH₂CH₂), 0.87 (t, *J* = 6.9 Hz, 3H, CH₂CH₃); ^{13}C-NMR (101 MHz, $CDCl_3$): δ 172.1, 171.1, 145.6, 127.3, 38.1, 32.0, 29.64, 29.61, 29.4, 29.3, 28.7, 26.9, 22.8,

14.2, 11.1; MS (ESI, *m/z)* cald. for $C_{16}H_{30}NO_3^+$ [M + CH_3OH + H]$^+$ = 284.2, found [M + CH_3OH + H]$^+$ = 284.3.

1-Dodecyl-3-methylenepyrrolidine-2,5-dione (17a) and 1-dodecyl-3-methyl-1*H*-pyrrole-2,5-dione (17b). According to the general procedure for synthesis of imides, dodecylamine (2.05 mL, 8.92 mmol, 1.0 equiv.) and itaconic anhydride (1.00 g, 8.92 mmol, 1.0 equiv.) in $CHCl_3$ (64.2 mL) afforded a white solid that was treated with Ac_2O (2.95 mL, 31.2 mmol, 3.5 equiv.) and NaOAc (366 mg, 4,46 mmol, 0.5 equiv.) and heated at 100 °C. The dark reaction mixture was cooled and poured into ice cold H_2O (50 mL) and extracted with EtOAc (3 x 50 mL). Purified by flash chromatography (EtOAc:hexane 1:9).

1-Dodecyl-3-methylenepyrrolidine-2,5-dione (17a)

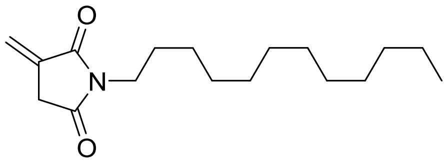

Dark red semi-solid, 4% yield; R_f = 0.28 (EtOAc:hexane 1:9; UV); IR (neat) ν (cm^{-1}): 3093, 2914, 2847, 1695, 1663, 1344; ^{1}H-NMR (400 MHz, $CDCl_3$): δ 6.33 (t, *J* = 2.2 Hz, 1H, CCH**H**), 5.61 (t, *J* = 2.2 Hz, 1H, CC**H**H), 3.56 (t, *J* = 7.5 Hz, 2H, $NC\mathbf{H_2}CH_2$), 3.30 (t, *J* = 2.2 Hz, 2H, $C(O)C\mathbf{H_2}C(CH_2)C(O)$), 1.62-1.55 (m, 2H, $NCH_2C\mathbf{H_2}$), 1.29-1.24 (m, 18H, $C\mathbf{H_2}C\mathbf{H_2}C\mathbf{H_2}C\mathbf{H_2}C\mathbf{H_2}C\mathbf{H_2}C\mathbf{H_2}C\mathbf{H_2}C\mathbf{H_2}$), 0.87 (t, *J* = 6.9 Hz, 3H, $CH_2C\mathbf{H_3}$); ^{13}C-NMR (101 MHz, $CDCl_3$): δ 174.0, 169.7, 133.5, 120.5, 39.1, 33.9, 32.1, 29.76, 29.75, 29.7, 29.6, 29.5, 29.3, 27.9, 27.0, 22.8, 14.2; MS (ESI, *m/z)* cald. for $C_{17}H_{30}NO_2^+$ [M + H]$^+$ = 280.2, found [M + H]$^+$ = 280.1.

1-Dodecyl-3-methyl-1*H*-pyrrole-2,5-dione (17b)

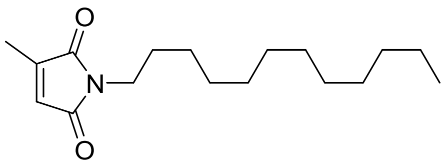

Pink semi-solid, 12% yield; R_f = 0.29 (EtOAc:hexane 1:9; UV); IR (neat) ν (cm^{-1}): 3081, 2915, 2848, 1685, 1638, 1376; ^{1}H-NMR (400 MHz, $CDCl_3$): δ 6.29 (q, *J* = 1.8 Hz, 1H, $C(O)C\mathbf{H}C(CH_3)C(O)$), 3.47 (t, *J* = 7.3 Hz, 2H, $NC\mathbf{H_2}CH_2$), 2.07 (d, *J* = 1.8 Hz, 3H, $C(O)CHC(C\mathbf{H_3})C(O)$), 1.59-1.52 (m, 2H, $NCH_2C\mathbf{H_2}$), 1.27-1.24 (m, 18H, $C\mathbf{H_2}C\mathbf{H_2}C\mathbf{H_2}C\mathbf{H_2}C\mathbf{H_2}C\mathbf{H_2}C\mathbf{H_2}C\mathbf{H_2}C\mathbf{H_2}$), 0.87 (t, *J* = 1.8 Hz, 3H, $CH_2C\mathbf{H_3}$); ^{13}C-NMR (101 MHz, $CDCl_3$): δ 172.0, 171.0, 145.4, 127.2, 38.0, 31.9, 29.61, 29.55, 29.5, 29.3, 29.1, 28.6, 26.8, 22.7, 14.1, 10.9; MS (ESI, *m/z)* cald. for $C_{17}H_{30}NO_2^+$ [M + H]$^+$ = 280.2, found [M + H]$^+$ = 280.0.

3-Methylene-1-tetradecylpyrrolidine-2,5-dione (18a) and 3-methyl-1-tetradecyl-1*H*-pyrrole-2,5-dione (18b). According to the general procedure for synthesis of imides, tetradecylamine (1.90 g, 8.92 mmol, 1.0 equiv.) and itaconic anhydride (1.00 g, 8.92 mmol, 1.0 equiv.) in $CHCl_3$ (68.2 mL) afforded at white white solid that was treated with Ac_2O (2.95 mL, 31.2 mmol, 3.5 equiv.) and NaOAc (366 mg, 4,46 mmol, 0.5 equiv.) and heated at 100 °C. The dark reaction mixture was cooled and poured into ice cold H_2O (50 mL) and extracted with EtOAc (3 x 50 mL). Purified by flash chromatography (hexane → EtOAc:hexane 1:9).

3-Methylene-1-tetradecylpyrrolidine-2,5-dione (18a)

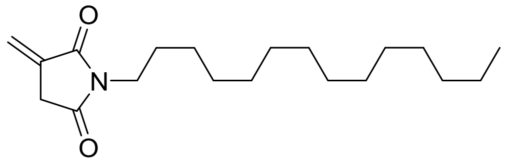

Light brown semi-solid, 2% yield; ^{1}H-NMR (400 MHz, $CDCl_3$): δ 6.33 (t, *J* = 2.5 Hz, 1H, CCH**H**), 5.61 (t, *J* = 2.0 Hz, 1H, CC**H**H), 3.56 (t, *J* = 7.5 Hz, 2H, NC**H₂**CH₂), 3.30 (t, *J* = 2.2 Hz, 2H, C(O)C**H₂**C(CH₂)C(O)), 1.61-1.55 (m, 2H, NCH₂C**H₂**), 1.29-1.24 (m, 22H, C**H₂**C**H₂**C**H₂**C**H₂**C**H₂**C**H₂**C**H₂**C**H₂**C**H₂**C**H₂**C**H₂**), 0.88 (t, *J* = 6.8 Hz, 3H, CH₂C**H₃**); ^{13}C-NMR (101 MHz, $CDCl_3$): δ 174.0, 169.7, 133.5, 120.4, 39.1, 33.9, 32.1, 29.82, 29.79, 29.76, 29.7, 29.6, 29.5, 29.3, 27.9, 27.0, 22.8, 14.2.

3-Methyl-1-tetradecyl-1*H*-pyrrole-2,5-dione (18b)

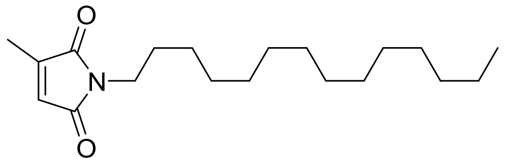

Light pink solid, 11% yield; mp. = 43-50 °C; IR (neat) ν (cm^{-1}): 3081, 2914, 2848, 1683, 1638, 1376; ^{1}H-NMR (400 MHz, $CDCl_3$): δ 6.29 (q, *J* = 1.8 Hz, 1H, C(O)C**H**C(CH₃)C(O)), 3.47 (t, *J* = 7.3 Hz, 2H, NC**H₂**CH₂), 2.07 (d, *J* = 1.8 Hz, 3H, C(O)CHC(C**H₃**)C(O)), 1.57-1.54 (m, 2H, NCH₂C**H₂**), 1.29-1.24 (m, 22H, C**H₂**C**H₂**C**H₂**C**H₂**C**H₂**C**H₂**C**H₂**C**H₂**C**H₂**C**H₂**C**H₂**), 0.87 (t, *J* = 6.8 Hz, 3H, C**H₃**); ^{13}C-NMR (101 MHz, $CDCl_3$): δ 172.0, 171.0, 145.5, 127.2, 38.0, 31.9, 29.7, 29.64, 29.62, 29.55, 29.5, 29.3, 29.2, 28.6, 26.8, 22.7, 14.1, 11.0; MS (ESI, *m/z)* cald. for $C_{20}H_{38}NO_3^+$ $[M + CH_3OH + H]^+$ = 340.3, found $[M + CH_3OH + H]^+$ = 340.2.

4.3 ^{1}H and ^{13}C NMR spectra of synthesized compounds

^{1}H- and ^{13}C-NMR spectra of compound **1a**

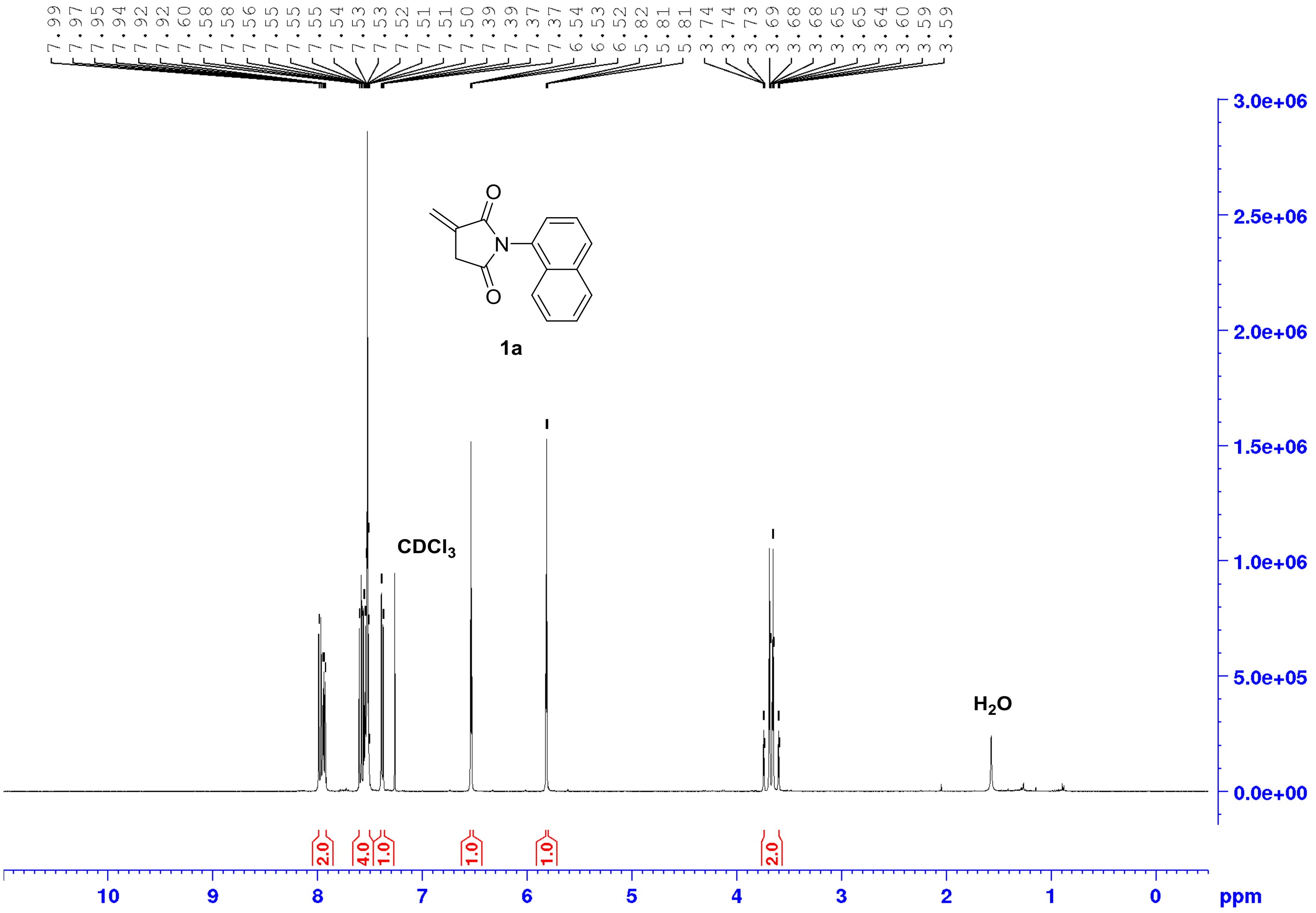

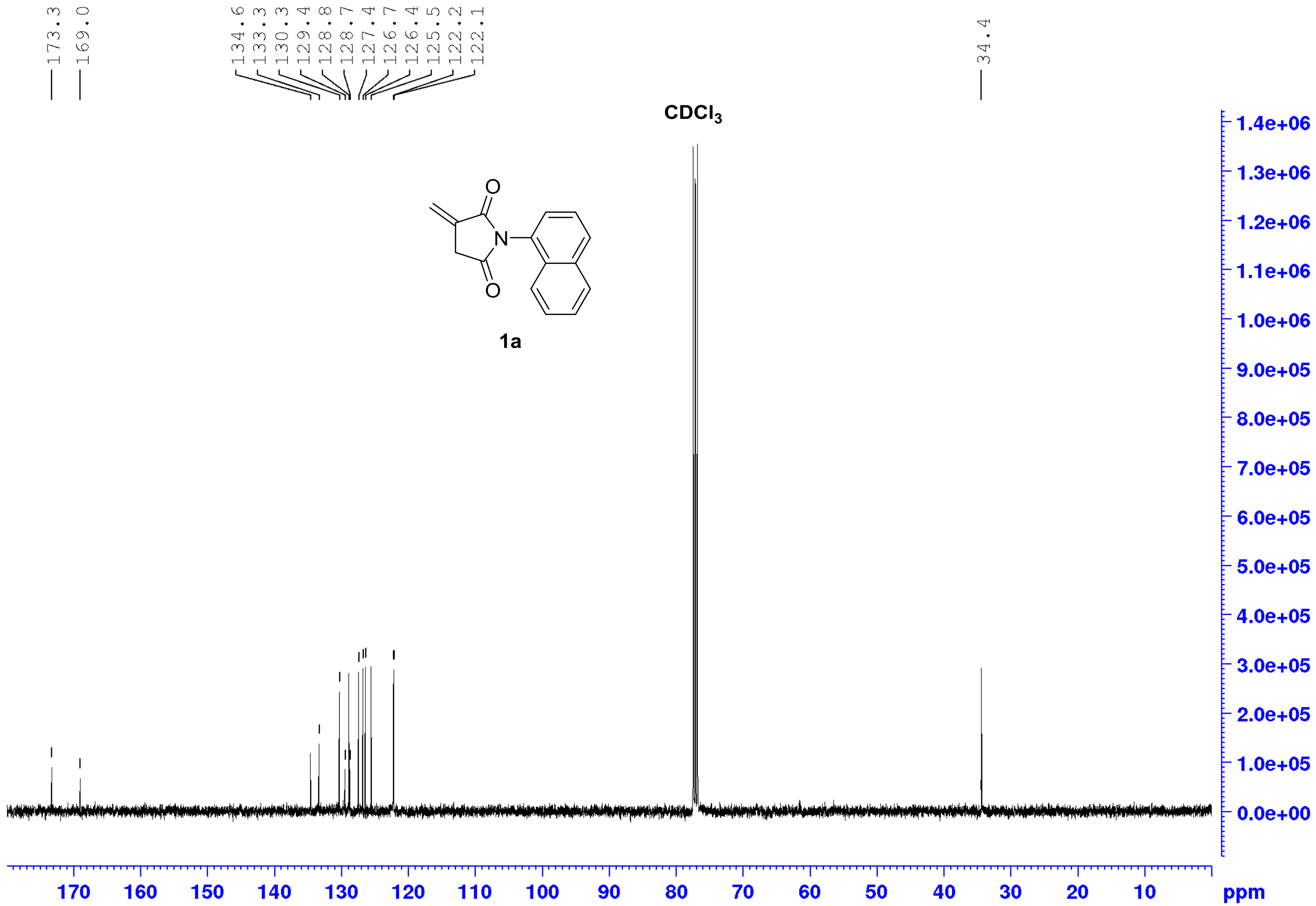

173.3
169.0
134.6
133.3
130.3
129.4
128.8
128.7
127.4
126.7
126.4
125.5
122.2
122.1
34.4
CDCl$_3$
O
N
O
1a
1.4e+06
1.3e+06
1.2e+06
1.1e+06
1.0e+06
9.0e+05
8.0e+05
7.0e+05
6.0e+05
5.0e+05
4.0e+05
3.0e+05
2.0e+05
1.0e+05
0.0e+00
170 160 150 140 130 120 110 100 90 80 70 60 50 40 30 20 10
ppm

^{1}H- and ^{13}C-NMR spectra of compound **1b**

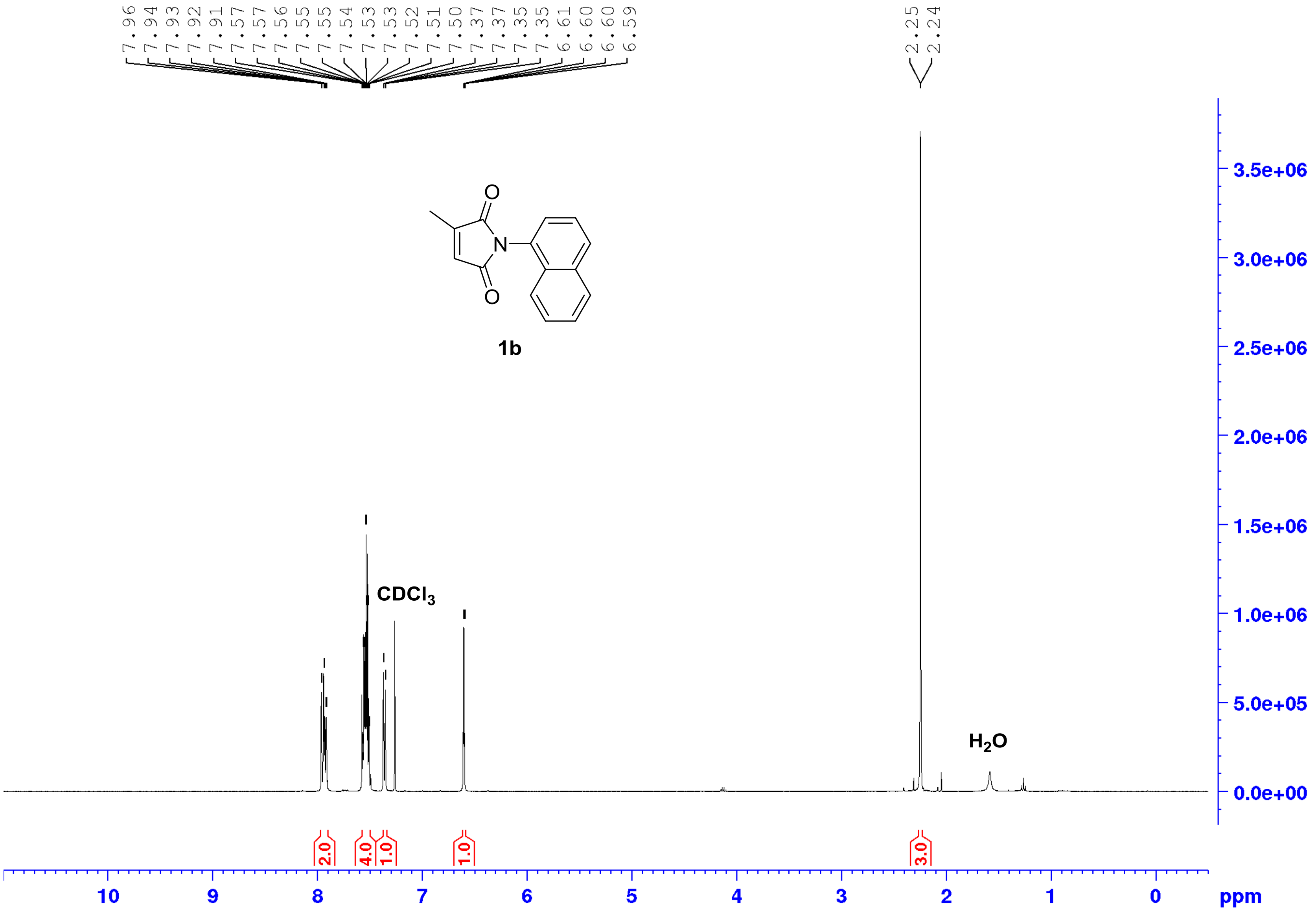

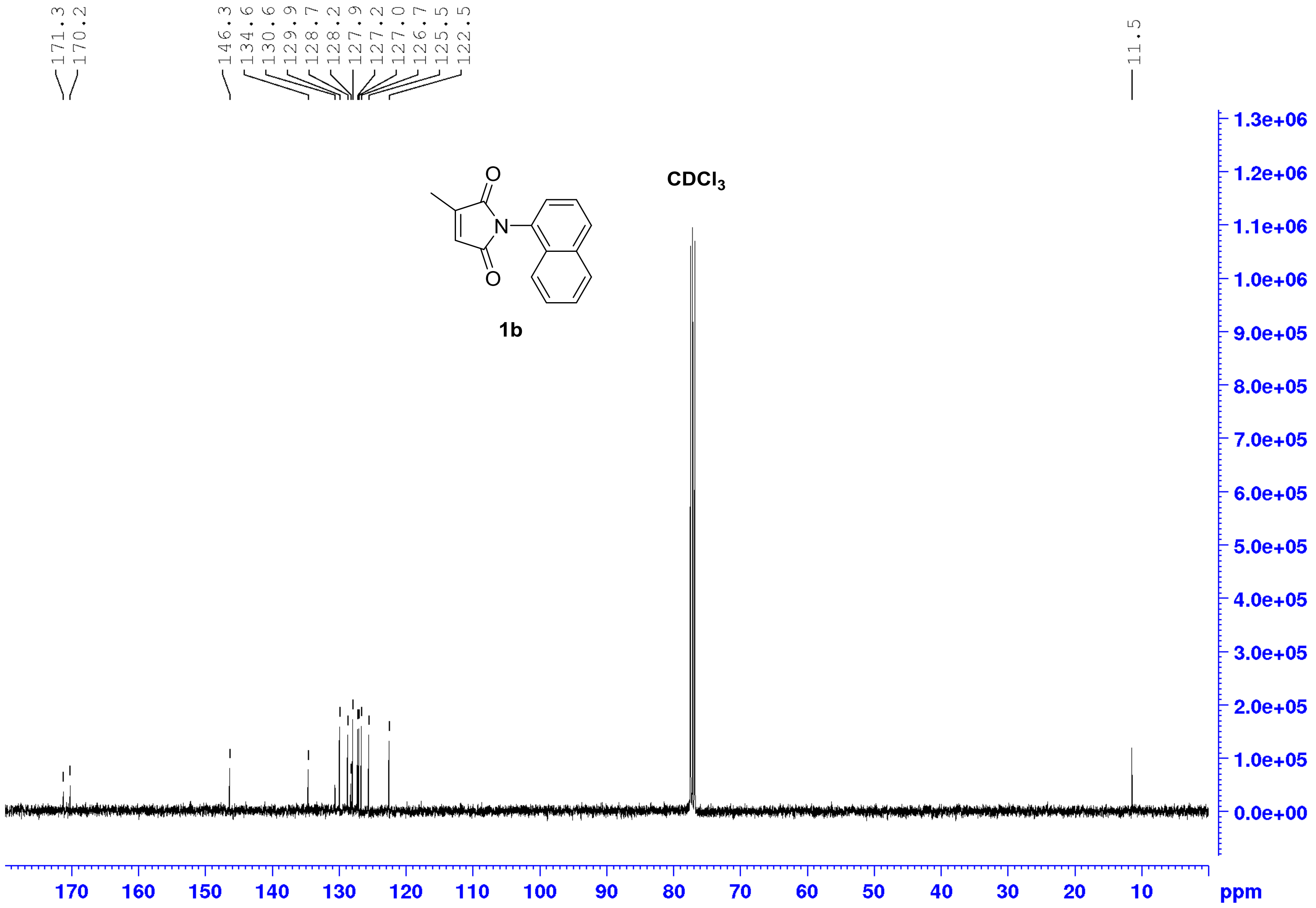
171.3
170.2
146.3
134.6
130.6
129.9
128.7
128.2
127.9
127.2
127.0
126.7
125.5
122.5
11.5
O
N
O
1b
CDCl3
1.3e+06
1.2e+06
1.1e+06
1.0e+06
9.0e+05
8.0e+05
7.0e+05
6.0e+05
5.0e+05
4.0e+05
3.0e+05
2.0e+05
1.0e+05
0.0e+00
170
160
150
140
130
120
110
100
90
80
70
60
50
40
30
20
10
ppm

^{1}H- and ^{13}C-NMR spectra of compound **2a**

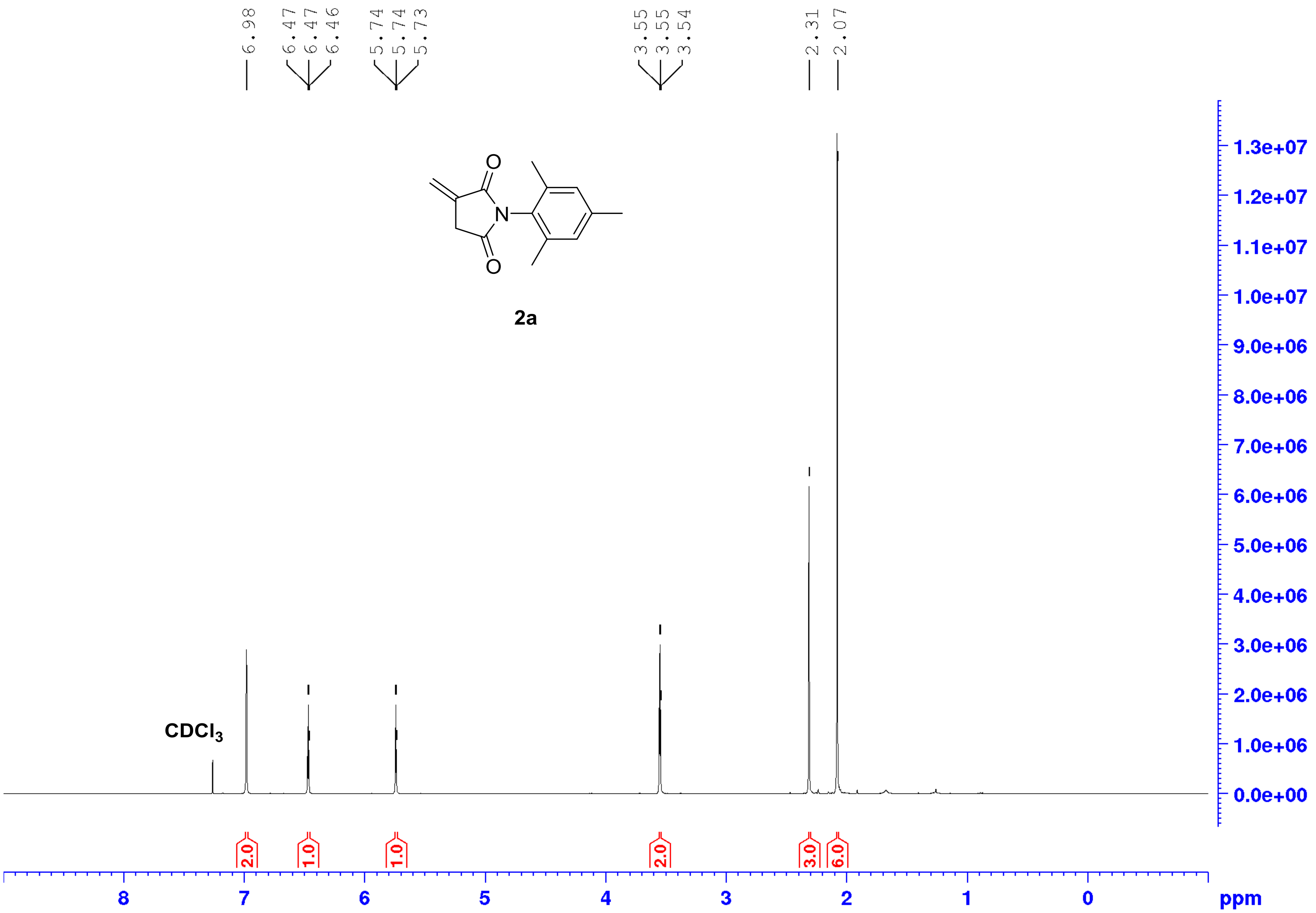

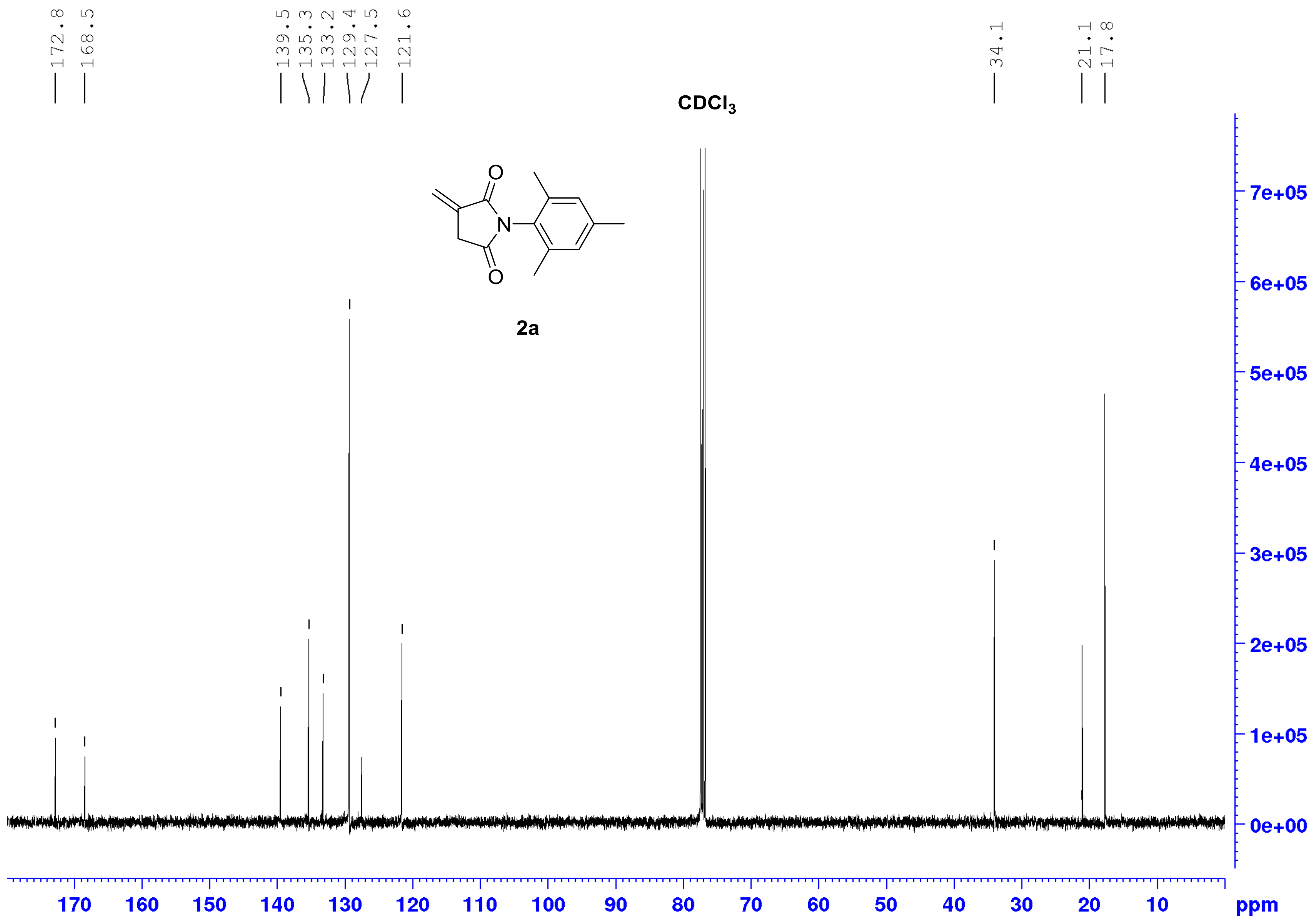

172.8
168.5
139.5
135.3
133.2
129.4
127.5
121.6
34.1
21.1
17.8
CDCl3
O
N
O
2a
7e+05
6e+05
5e+05
4e+05
3e+05
2e+05
1e+05
0e+00
170
160
150
140
130
120
110
100
90
80
70
60
50
40
30
20
10
ppm

^{1}H- and ^{13}C-NMR spectra of compound **3a**

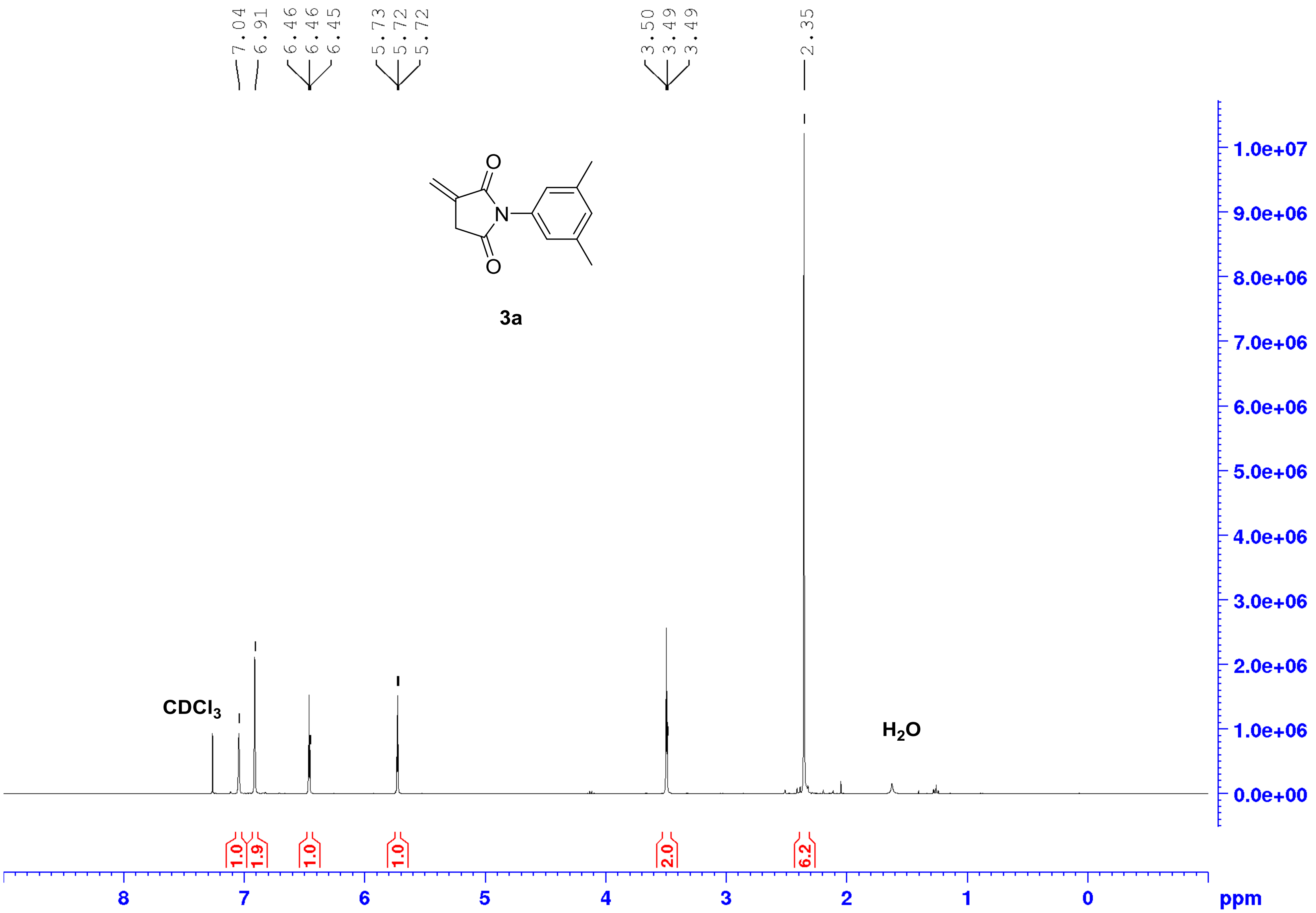

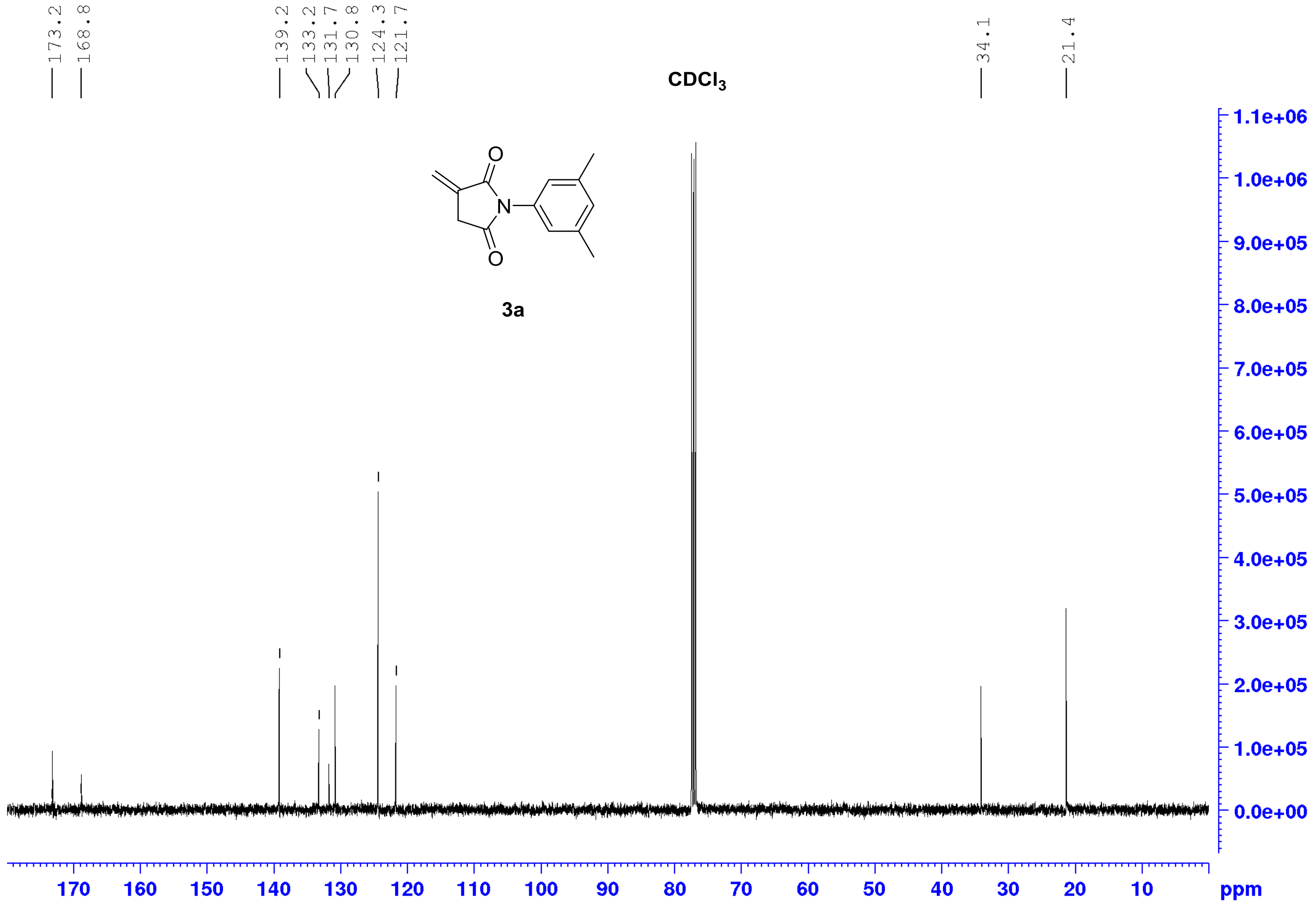

173.2
168.8
139.2
133.2
131.7
130.8
124.3
121.7
34.1
21.4
CDCl3
3a
1.1e+06
1.0e+06
9.0e+05
8.0e+05
7.0e+05
6.0e+05
5.0e+05
4.0e+05
3.0e+05
2.0e+05
1.0e+05
0.0e+00
170
160
150
140
130
120
110
100
90
80
70
60
50
40
30
20
10
ppm

^{1}H- and ^{13}C-NMR spectra of compound **4a**

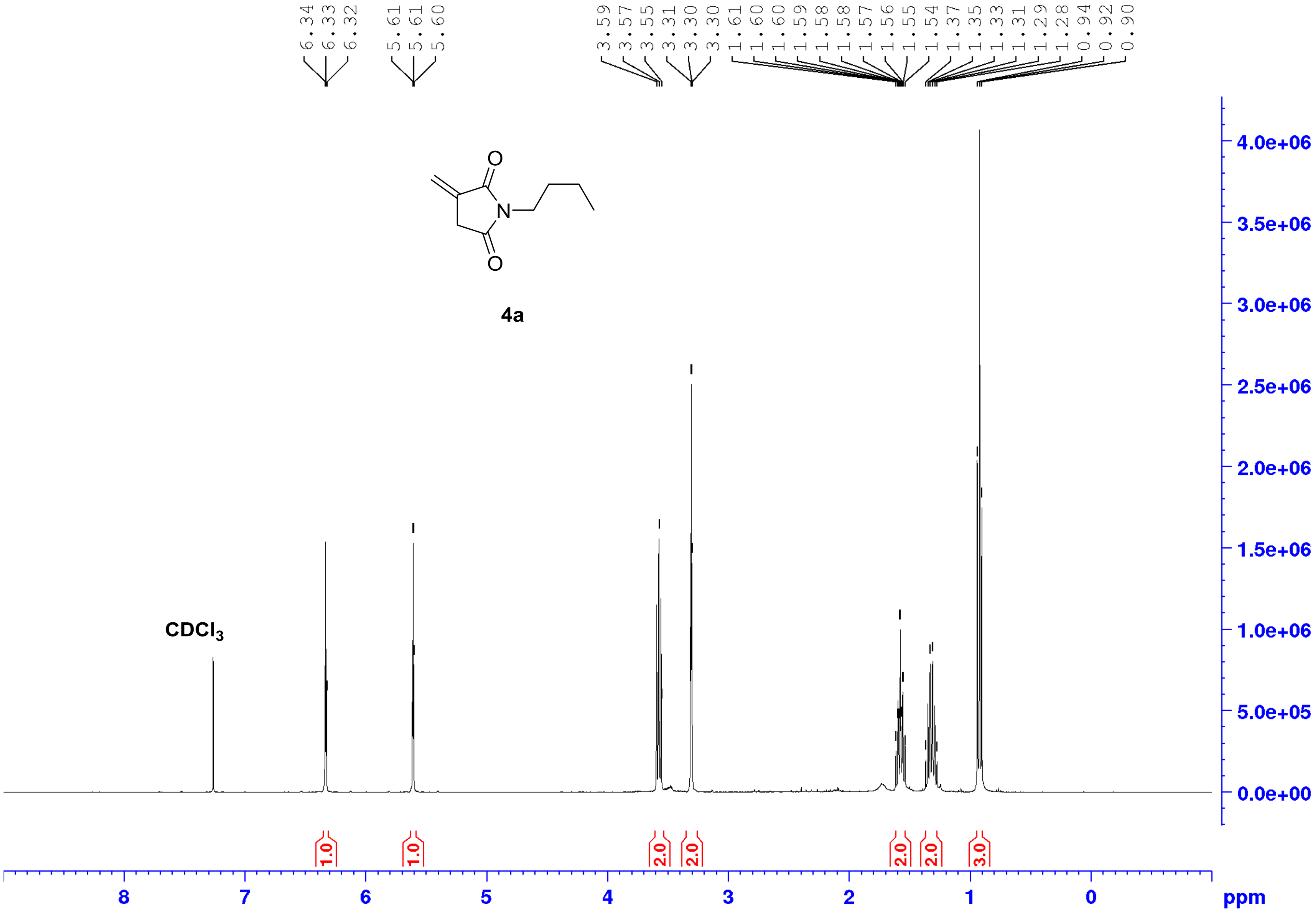

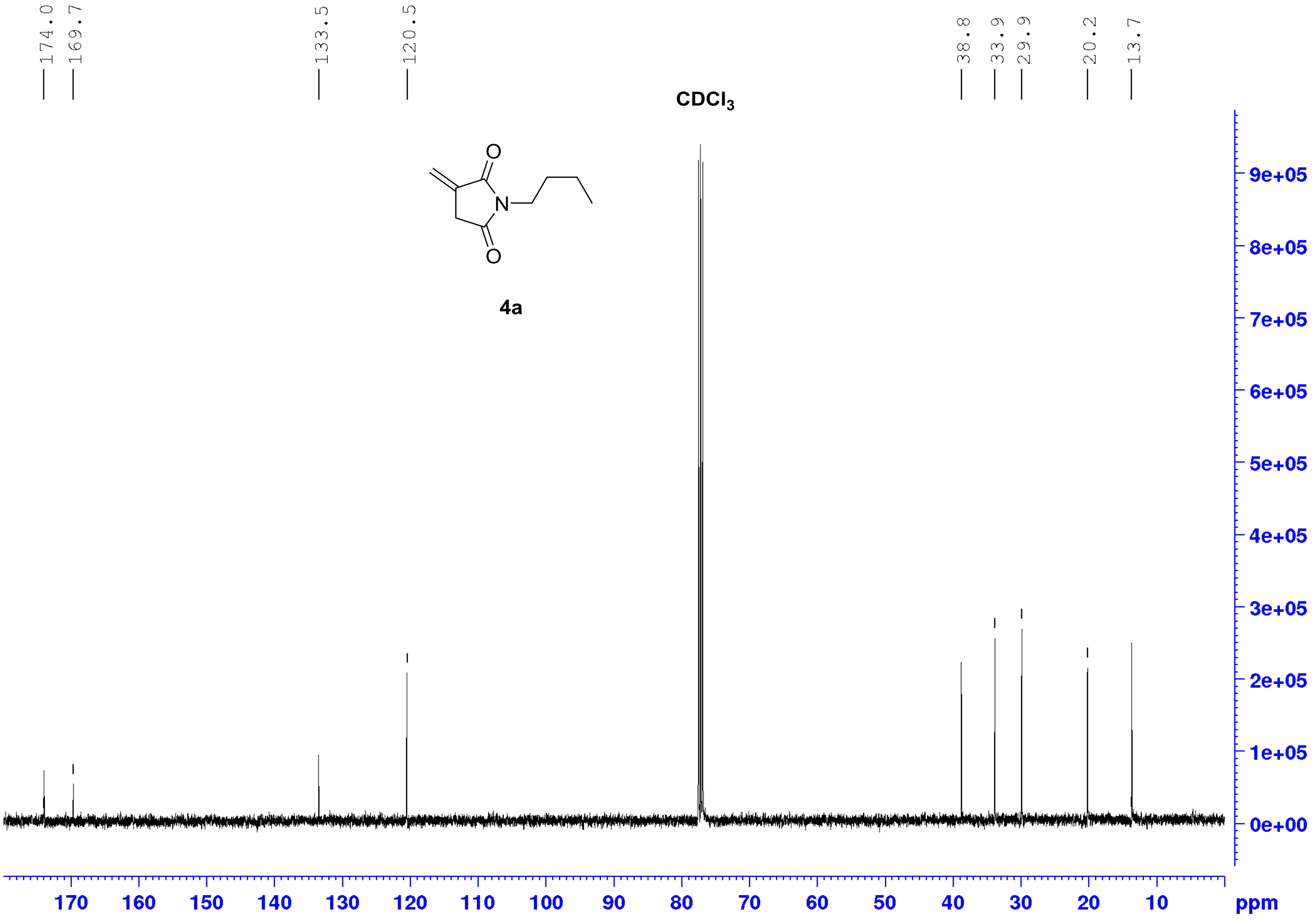

174.0
169.7
133.5
120.5
38.8
33.9
29.9
20.2
13.7
CDCl3
O
N
O
4a
9e+05
8e+05
7e+05
6e+05
5e+05
4e+05
3e+05
2e+05
1e+05
0e+00
170
160
150
140
130
120
110
100
90
80
70
60
50
40
30
20
10
ppm

^{1}H- and ^{13}C-NMR spectra of compound **5a**

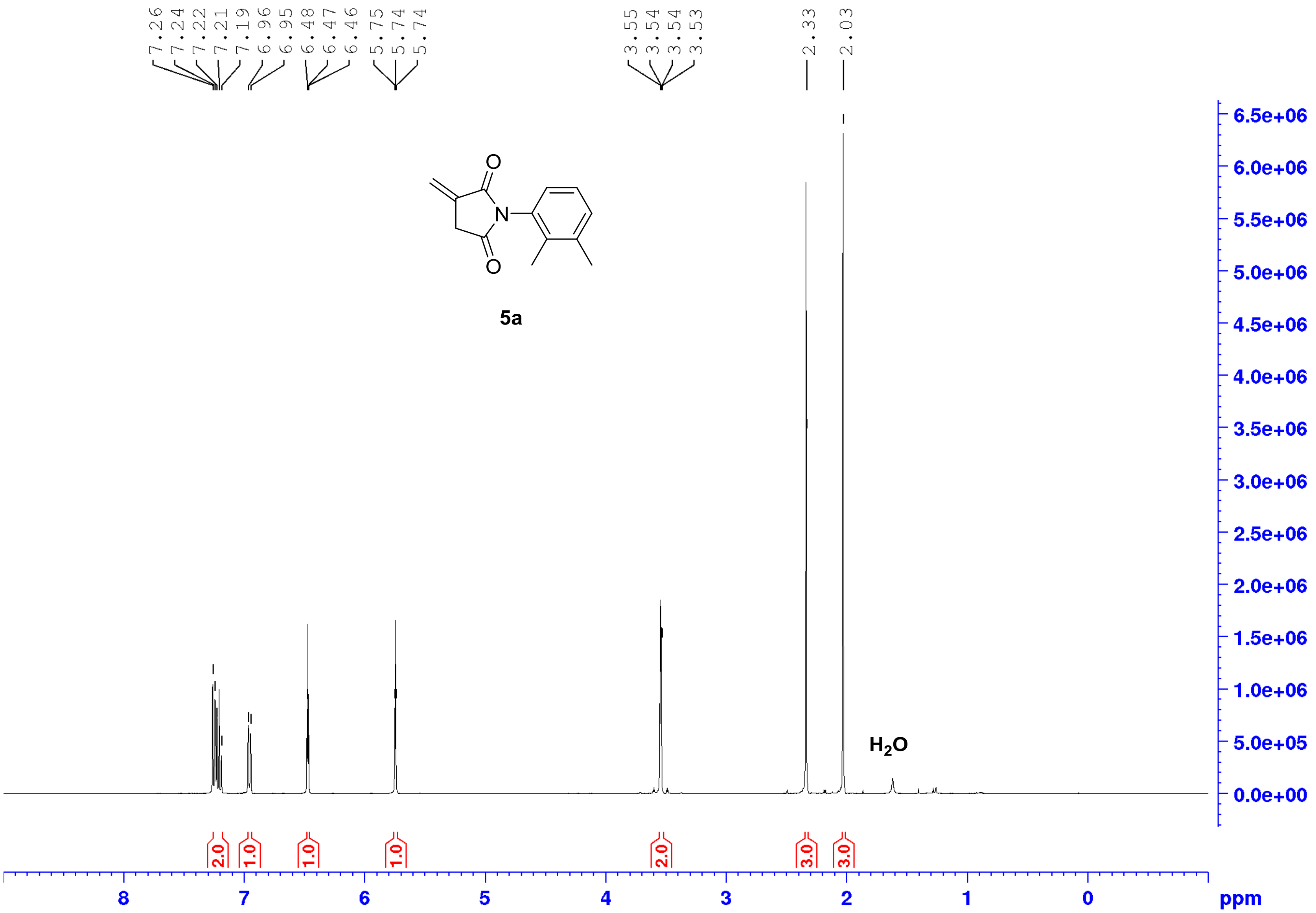

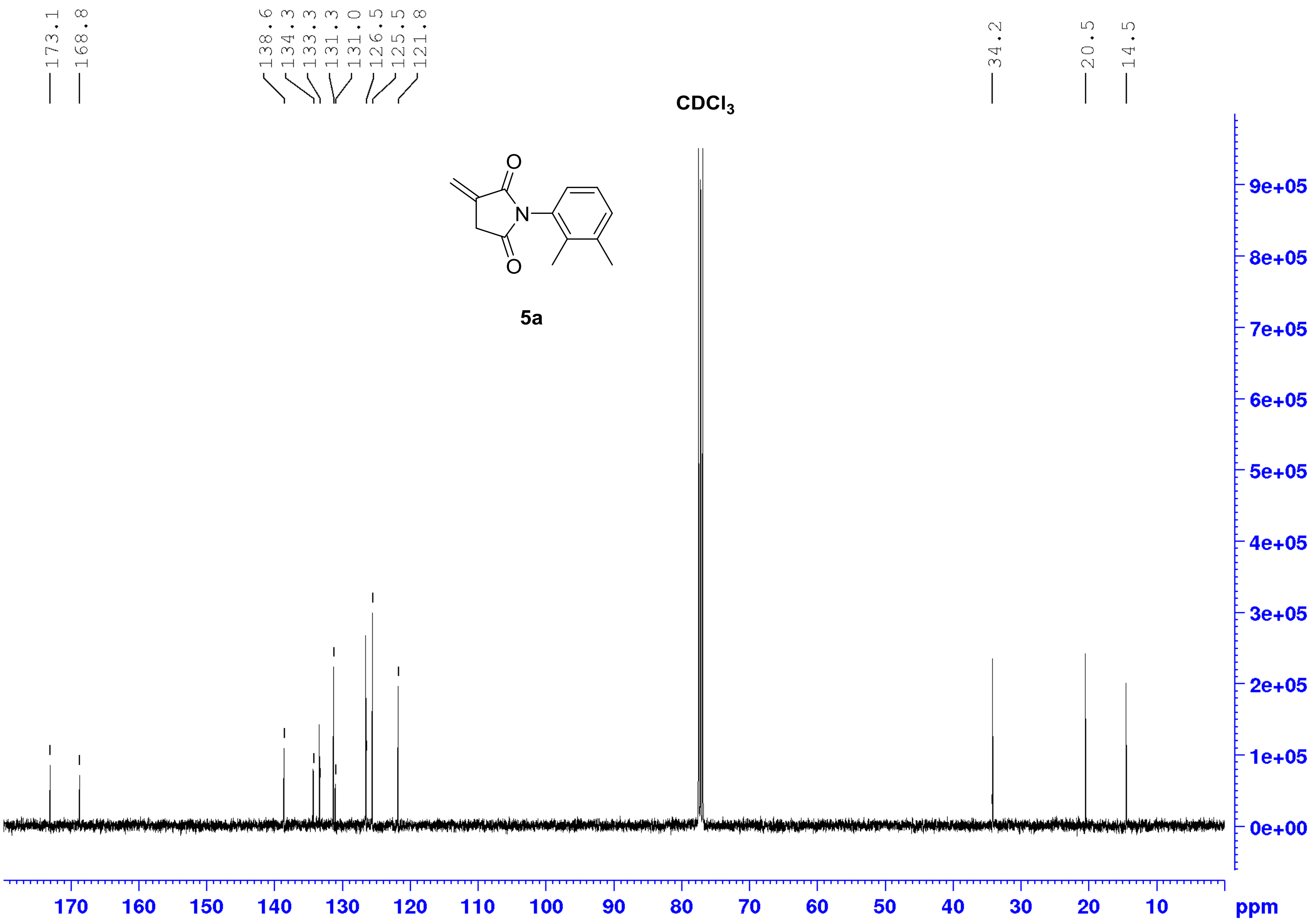

173.1
168.8
138.6
134.3
133.3
131.3
131.0
126.5
125.5
121.8
34.2
20.5
14.5
CDCl3
O
N
O
5a
9e+05
8e+05
7e+05
6e+05
5e+05
4e+05
3e+05
2e+05
1e+05
0e+00
170
160
150
140
130
120
110
100
90
80
70
60
50
40
30
20
10
ppm

^{1}H- and ^{13}C-NMR spectra of compound **6a**

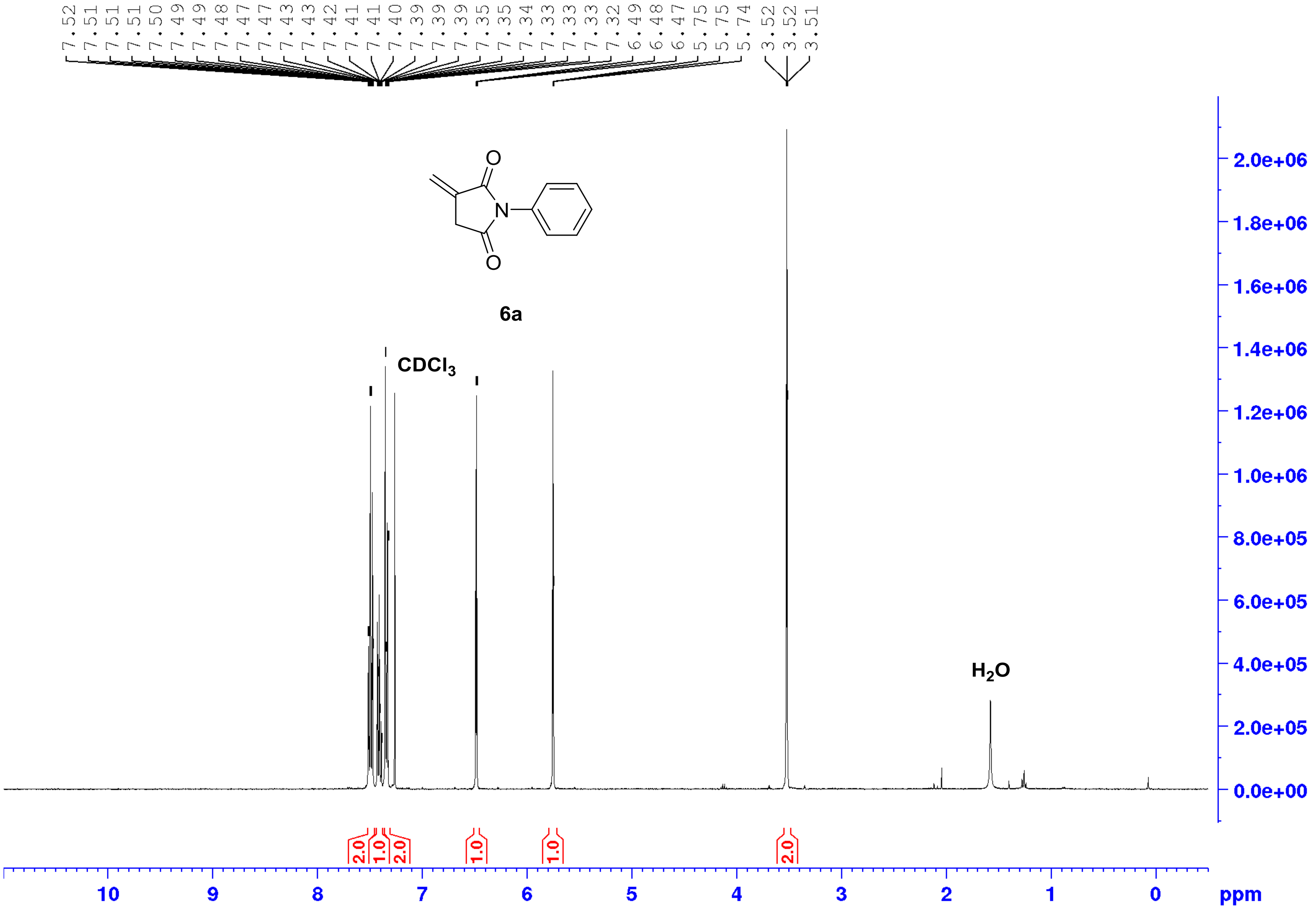

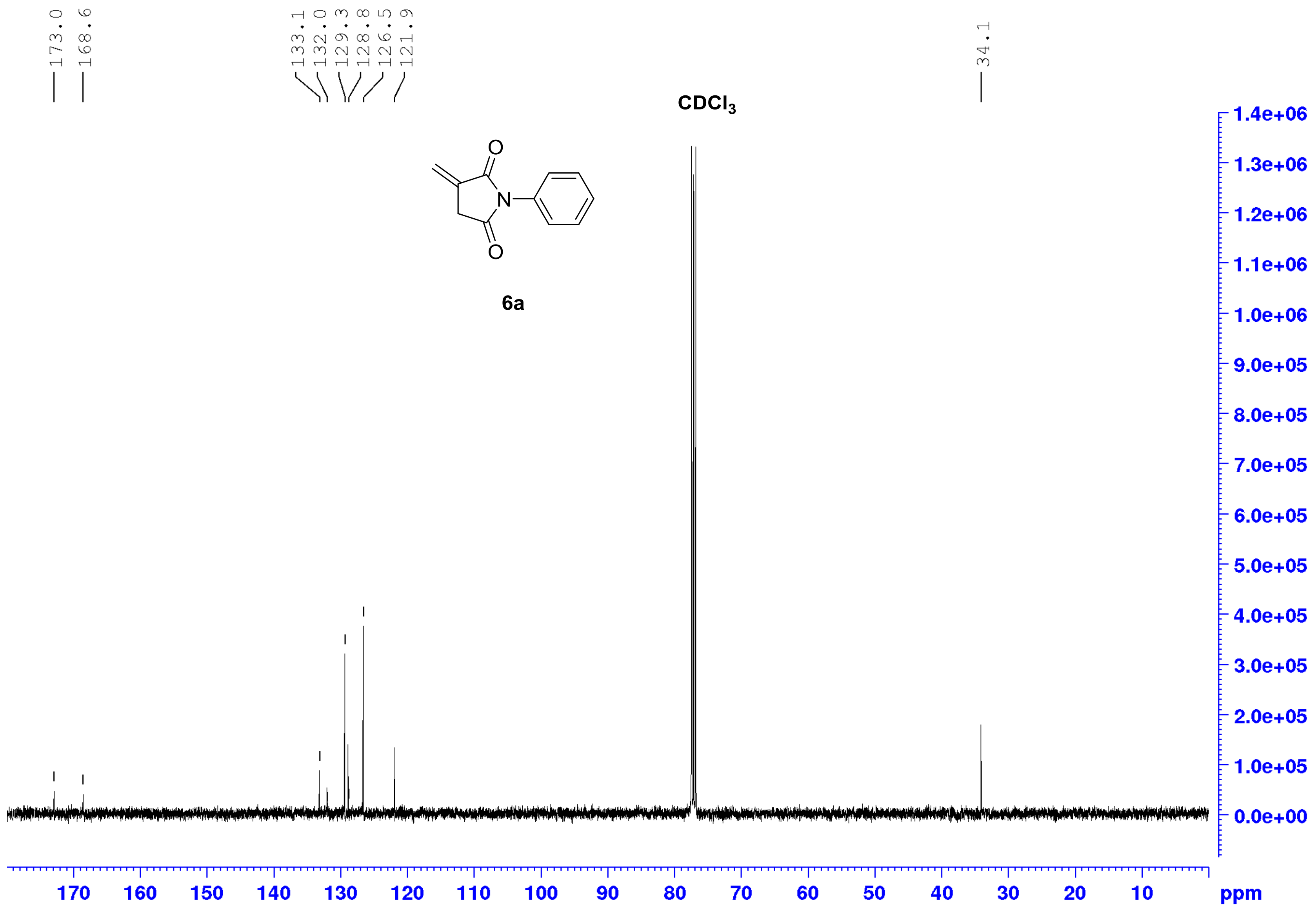

173.0
168.6
133.1
132.0
129.3
128.8
126.5
121.9
34.1
CDCl3
O
N
O
6a
1.4e+06
1.3e+06
1.2e+06
1.1e+06
1.0e+06
9.0e+05
8.0e+05
7.0e+05
6.0e+05
5.0e+05
4.0e+05
3.0e+05
2.0e+05
1.0e+05
0.0e+00
170 160 150 140 130 120 110 100 90 80 70 60 50 40 30 20 10
ppm

^{1}H- and ^{13}C-NMR spectra of compound **6b**

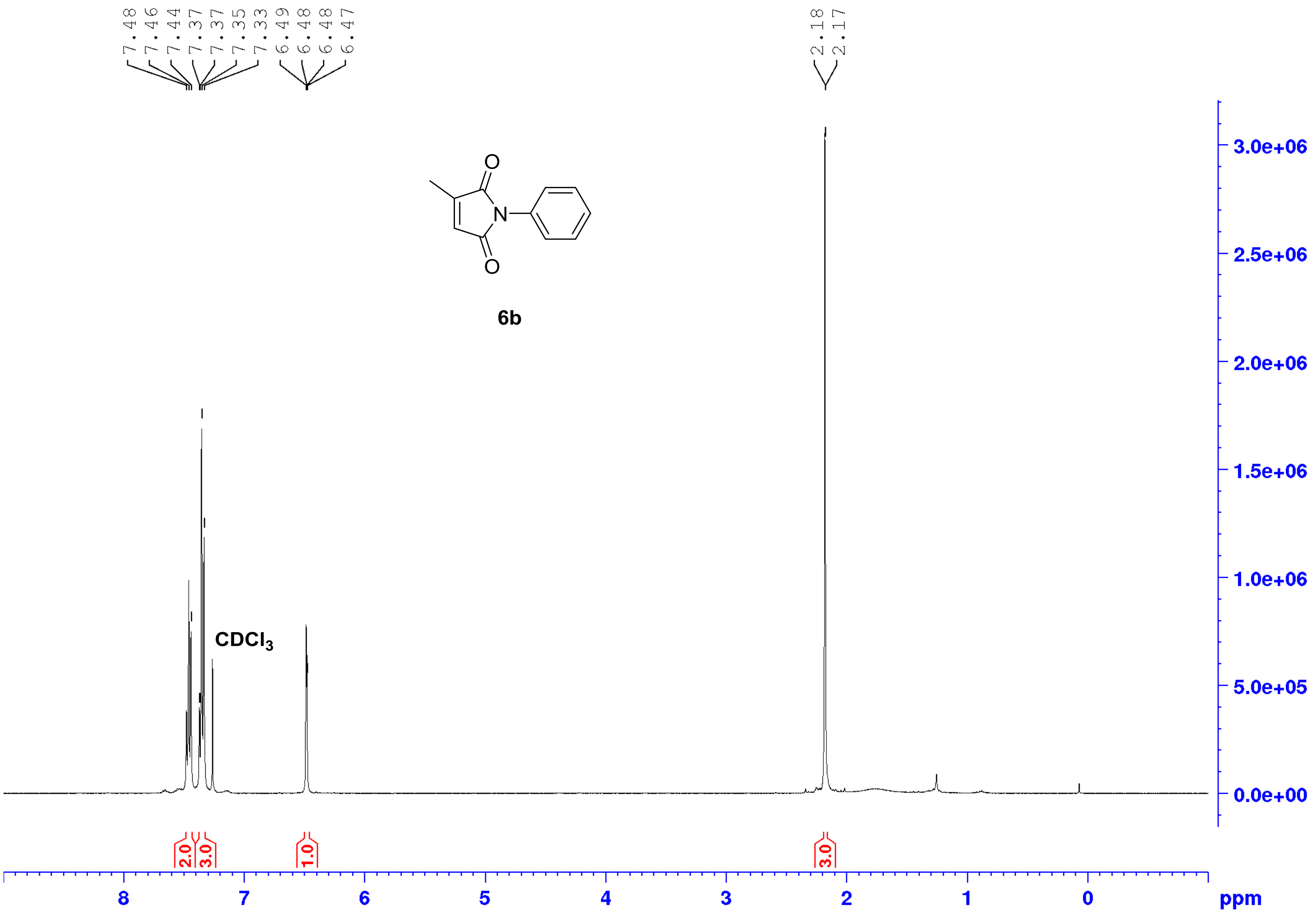

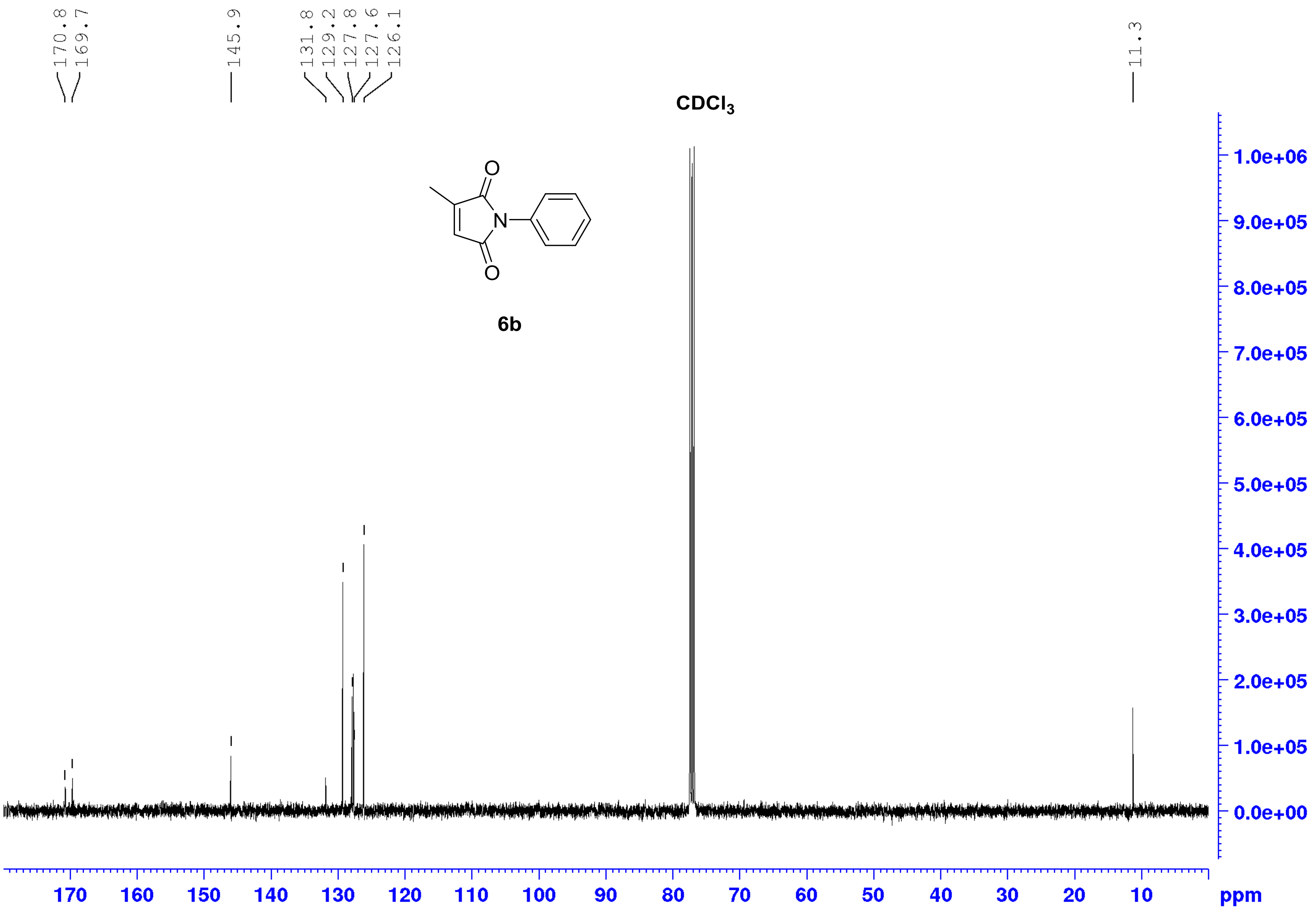

170.8
169.7
145.9
131.8
129.2
127.8
127.6
126.1
11.3
CDCl$_3$
O
N
O
6b
1.0e+06
9.0e+05
8.0e+05
7.0e+05
6.0e+05
5.0e+05
4.0e+05
3.0e+05
2.0e+05
1.0e+05
0.0e+00
170 160 150 140 130 120 110 100 90 80 70 60 50 40 30 20 10 ppm

^{1}H- and ^{13}C-NMR spectra of compound **8a**

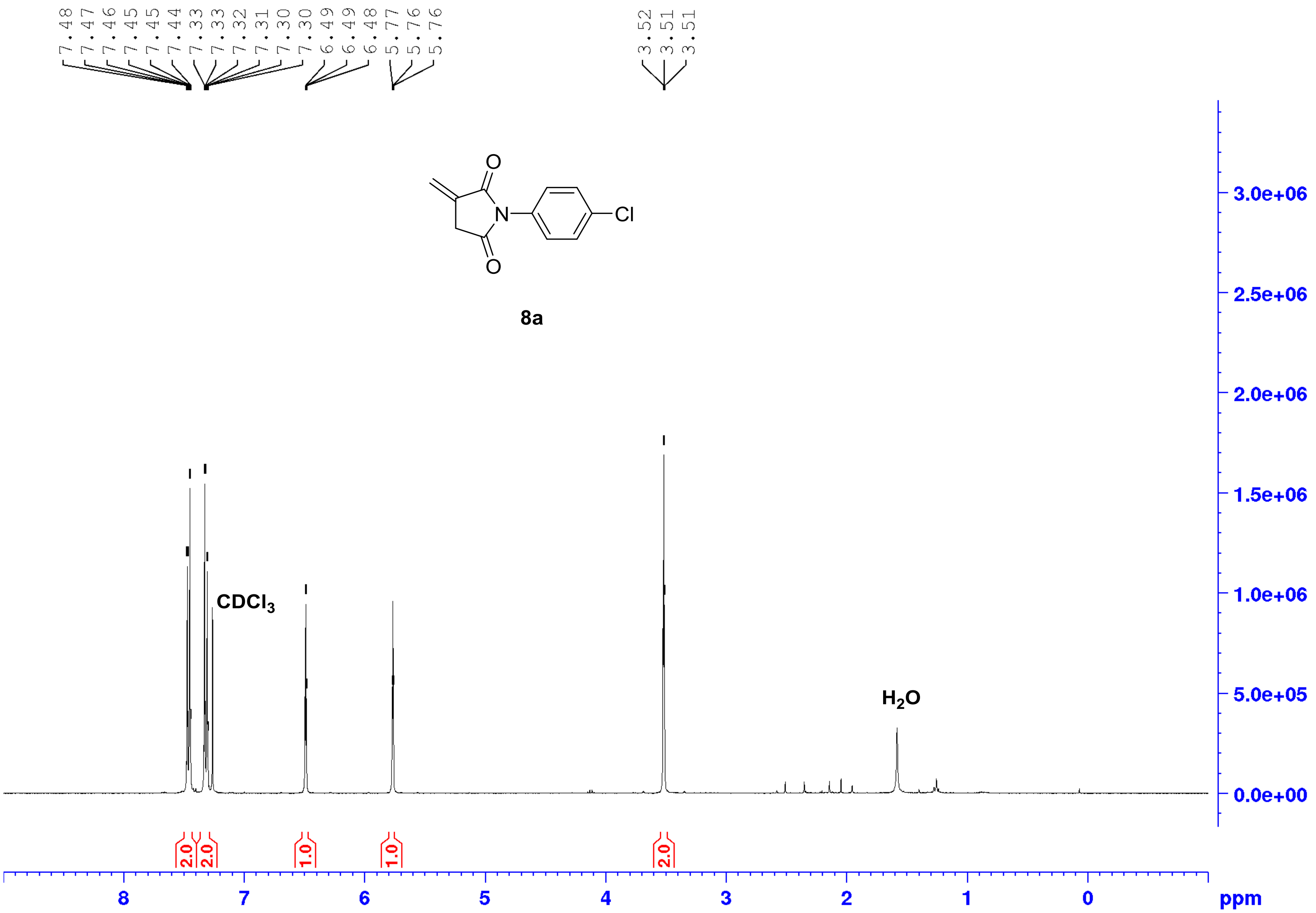

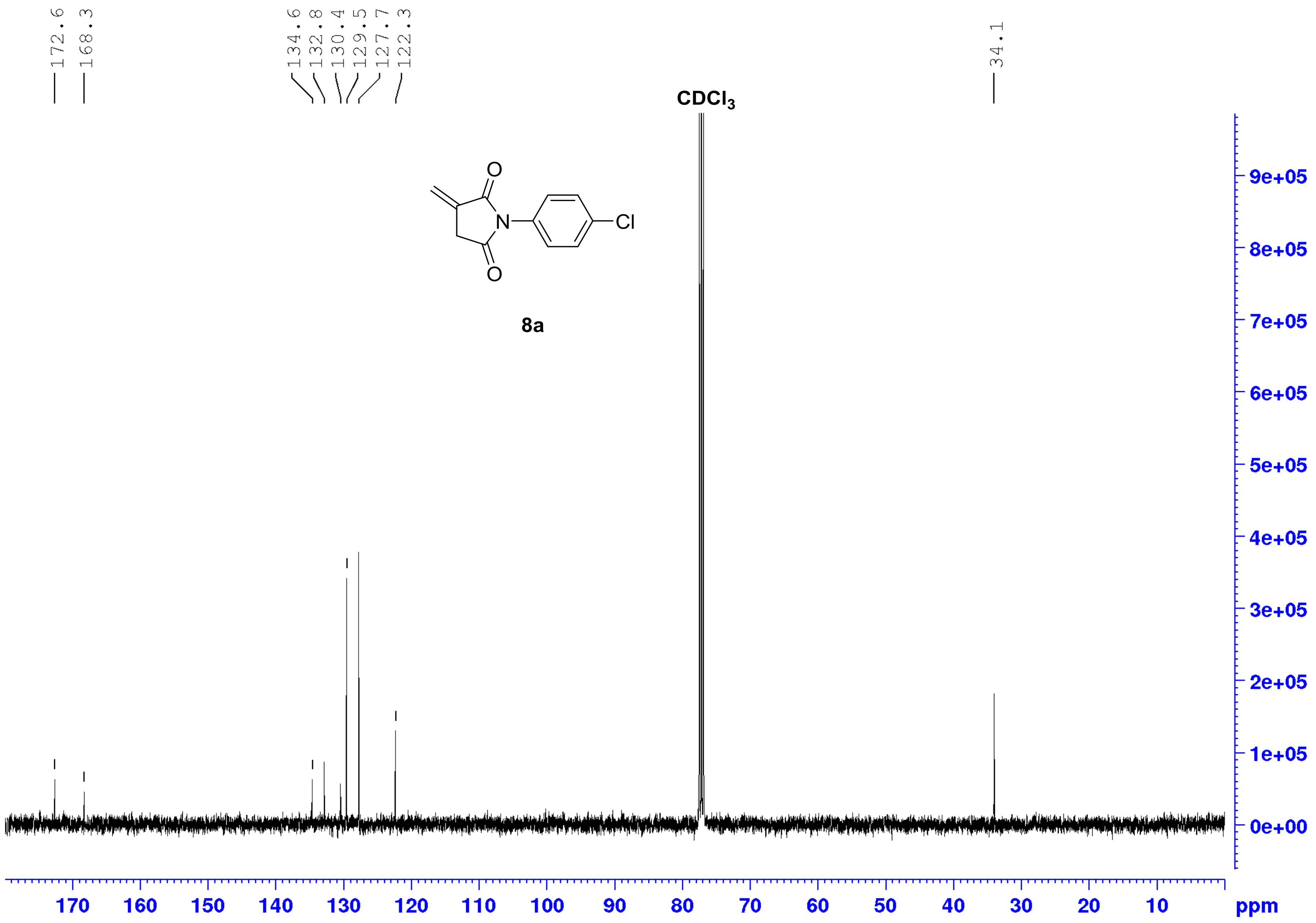

172.6
168.3
134.6
132.8
130.4
129.5
127.7
122.3
34.1
CDCl$_3$
O
N
Cl
O
8a
9e+05
8e+05
7e+05
6e+05
5e+05
4e+05
3e+05
2e+05
1e+05
0e+00
170
160
150
140
130
120
110
100
90
80
70
60
50
40
30
20
10
ppm

^{1}H- and ^{13}C-NMR spectra of compound **8b**

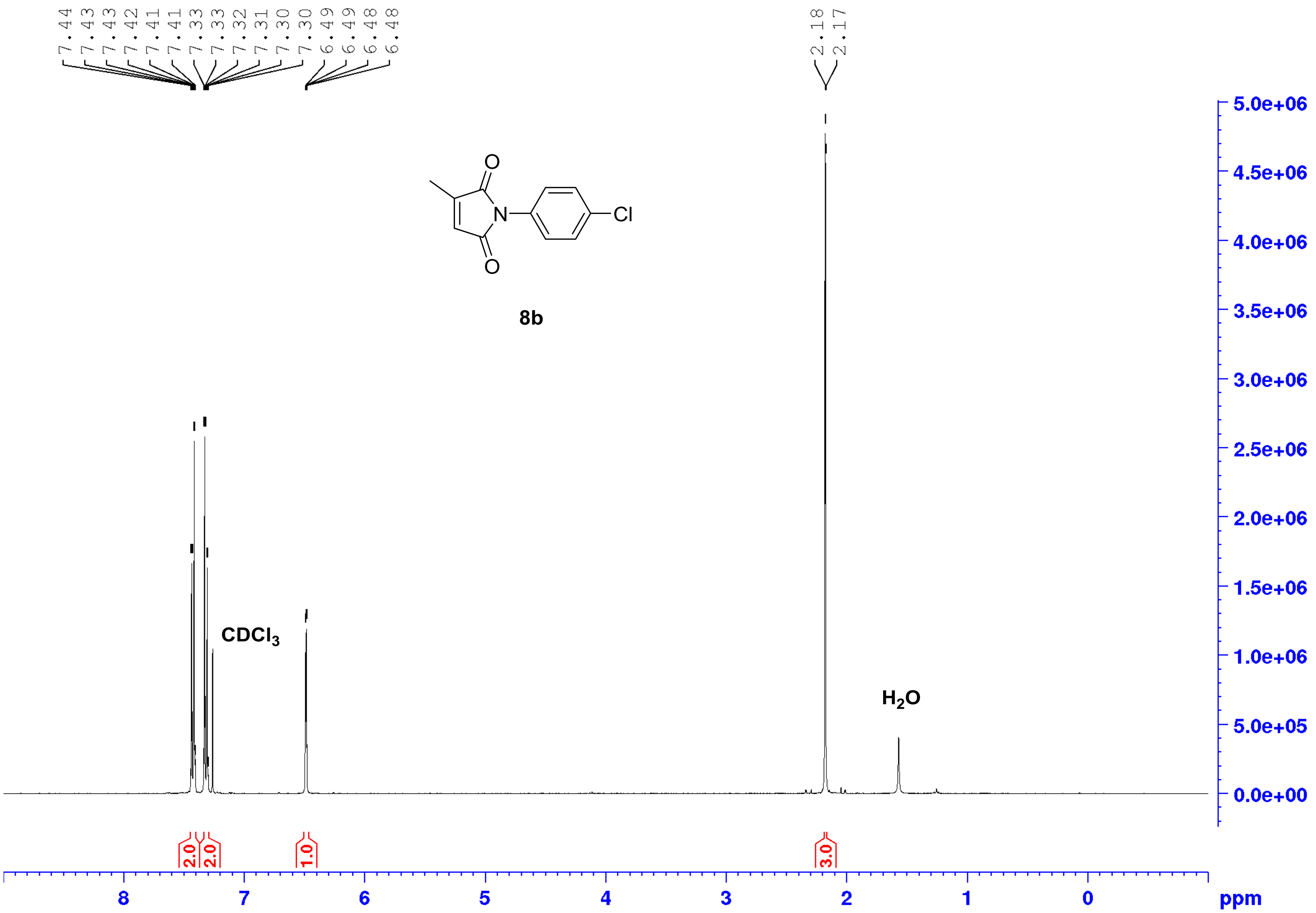

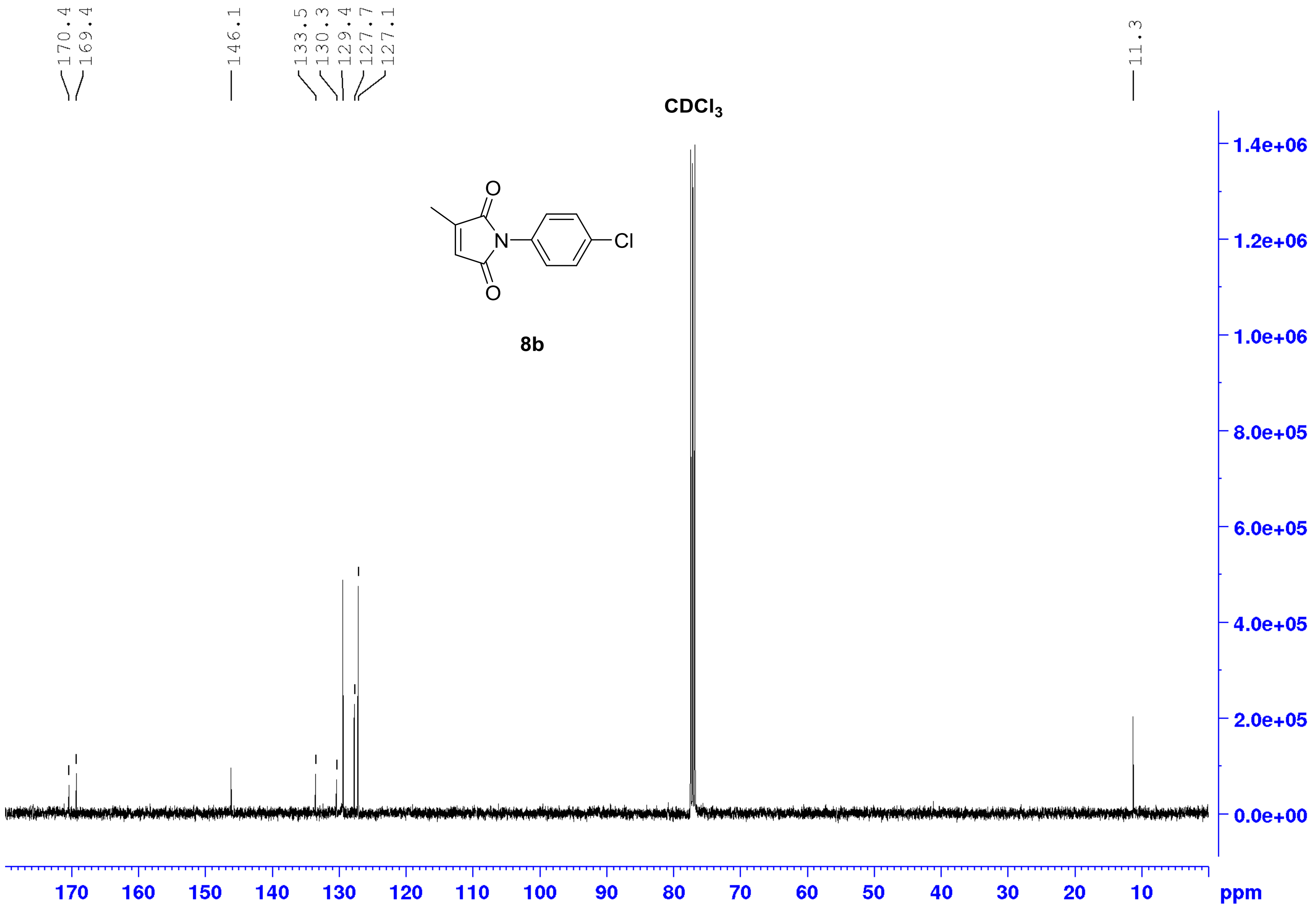
170.4
169.4
146.1
133.5
130.3
129.4
127.7
127.1
11.3
CDCl$_3$
O
N
Cl
O
8b
1.4e+06
1.2e+06
1.0e+06
8.0e+05
6.0e+05
4.0e+05
2.0e+05
0.0e+00
170 160 150 140 130 120 110 100 90 80 70 60 50 40 30 20 10
ppm

^{1}H- and ^{13}C-NMR spectra of compound **9a**

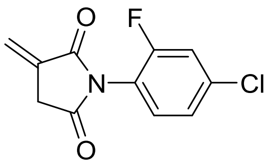

9a

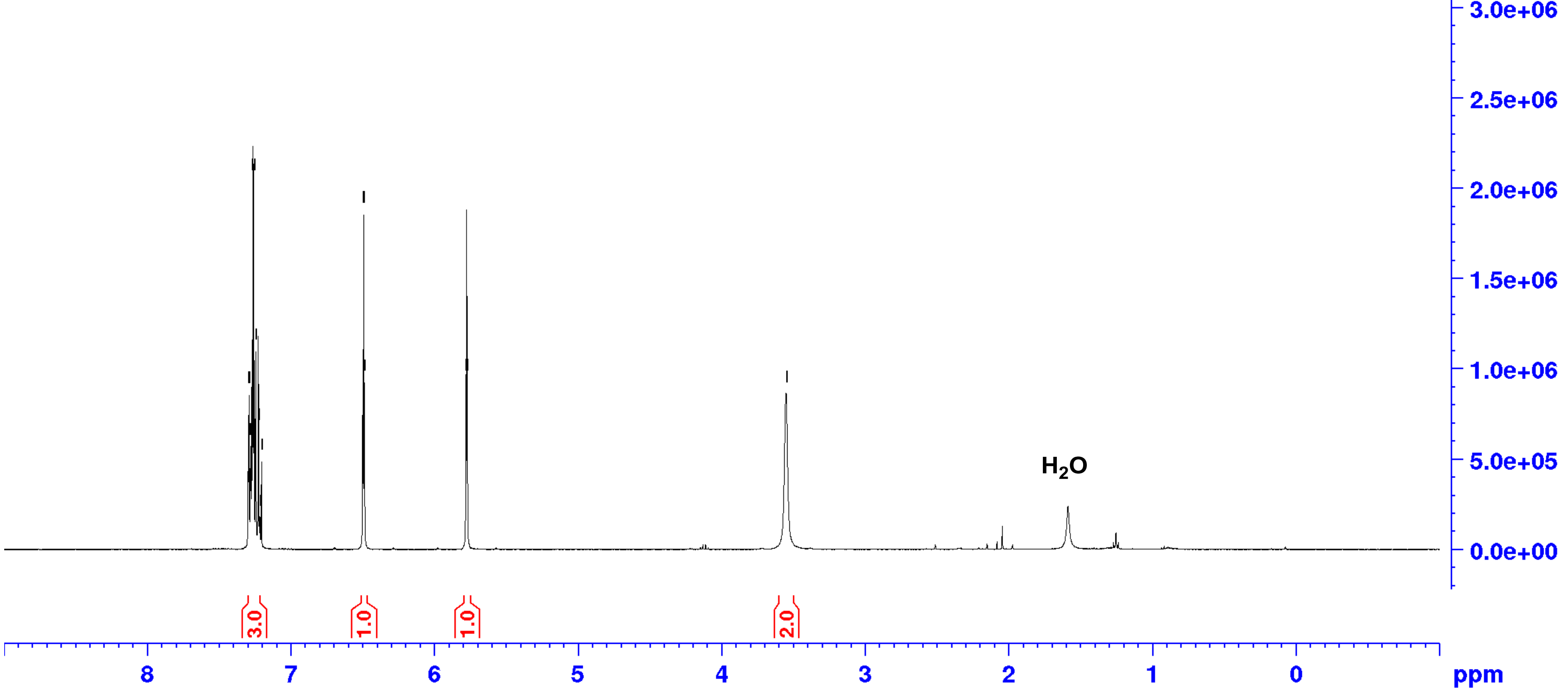

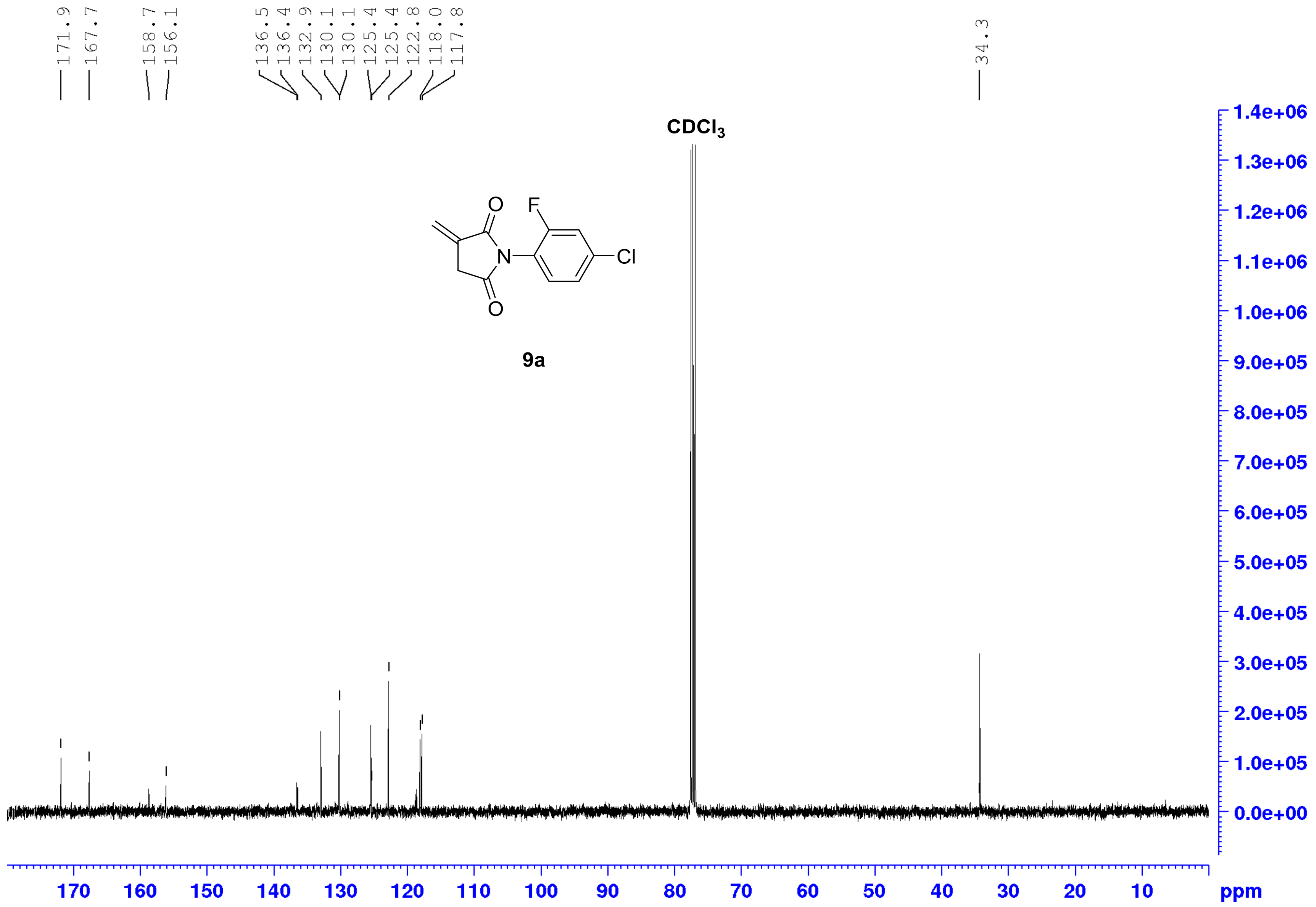

171.9
167.7
158.7
156.1
136.5
136.4
132.9
130.1
130.1
125.4
125.4
122.8
118.0
117.8
34.3
CDCl3
O
F
N
Cl
O
9a
1.4e+06
1.3e+06
1.2e+06
1.1e+06
1.0e+06
9.0e+05
8.0e+05
7.0e+05
6.0e+05
5.0e+05
4.0e+05
3.0e+05
2.0e+05
1.0e+05
0.0e+00
170 160 150 140 130 120 110 100 90 80 70 60 50 40 30 20 10
ppm

^{1}H- and ^{13}C-NMR spectra of compound **9b**

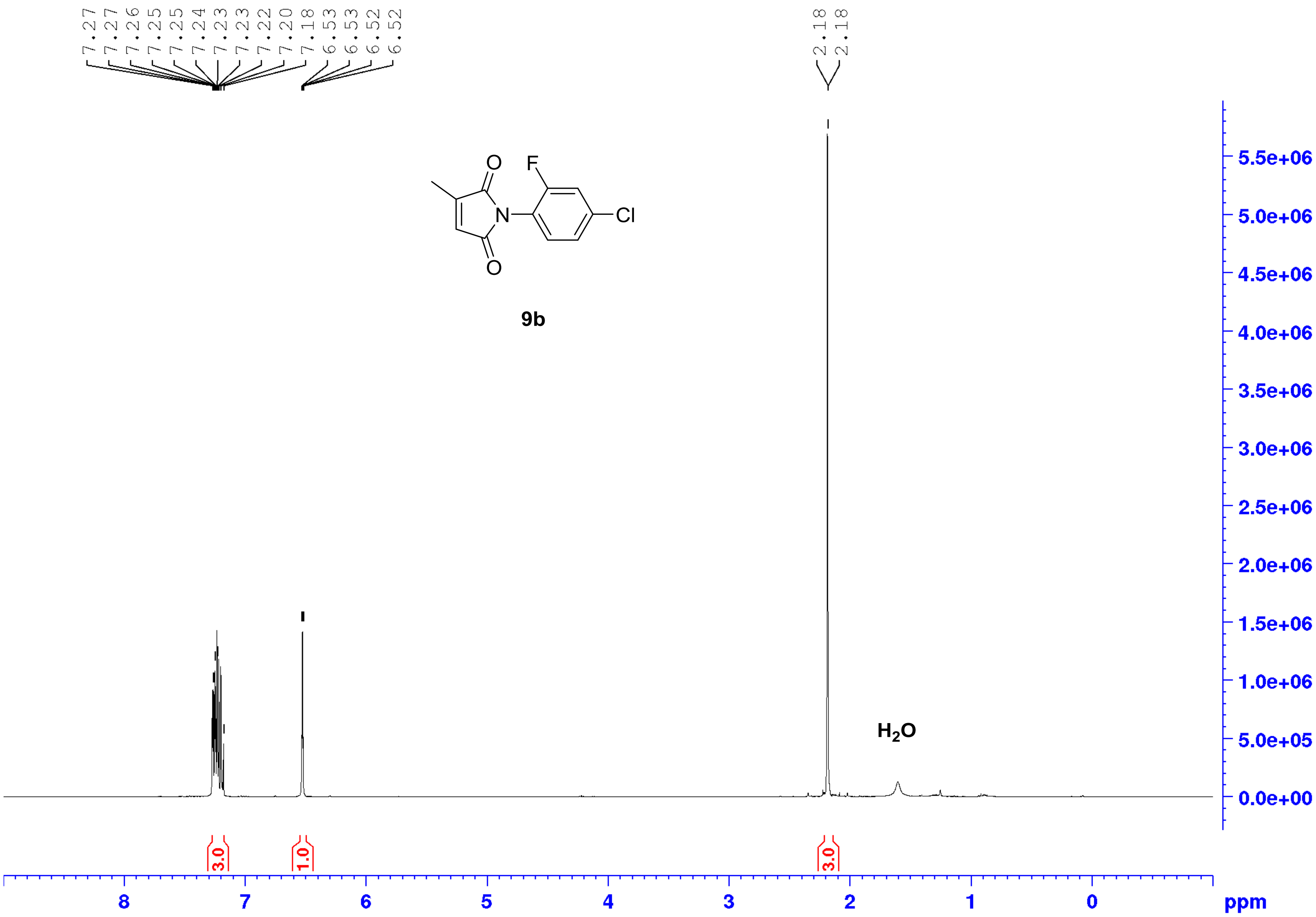

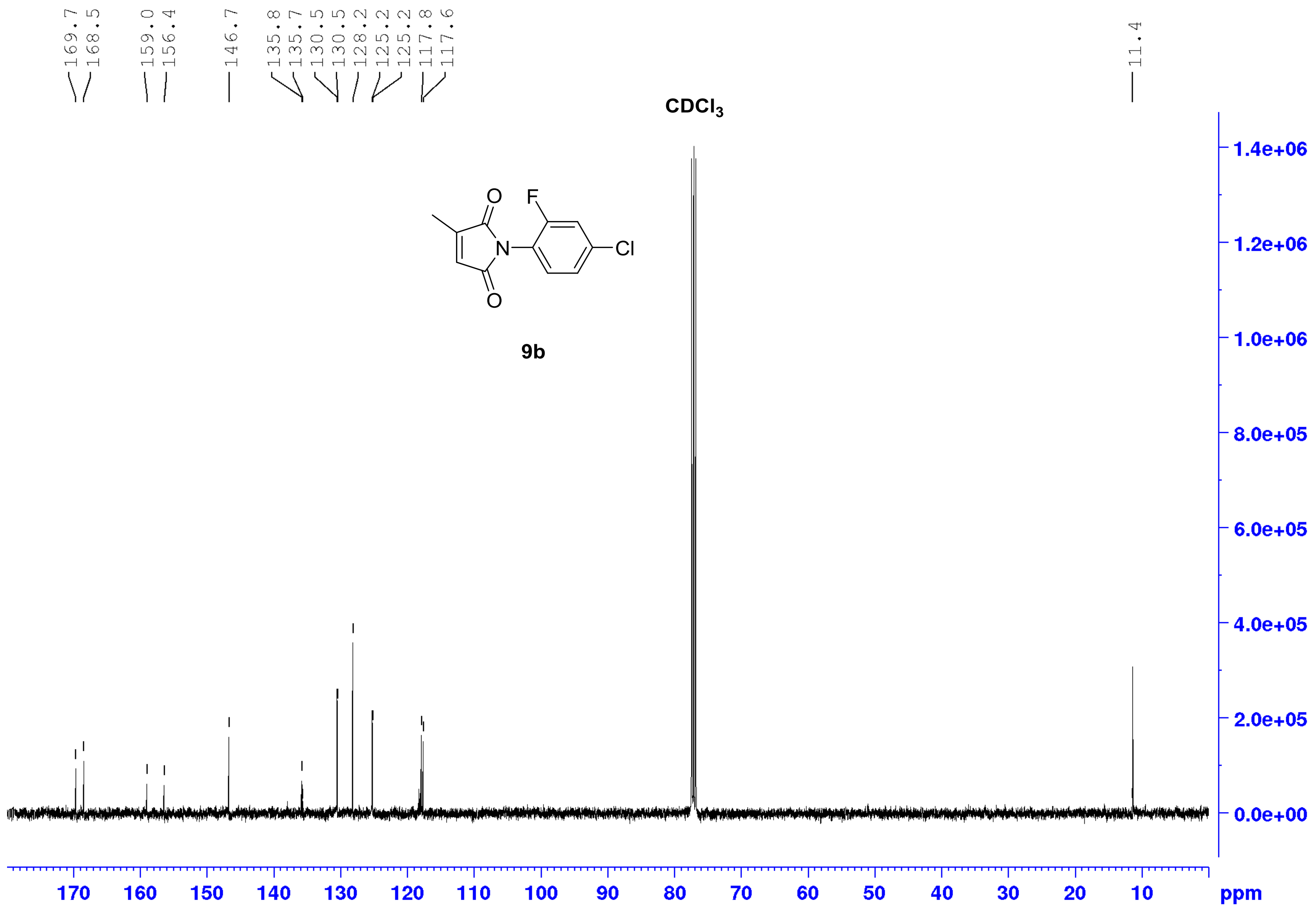
169.7
168.5
159.0
156.4
146.7
135.8
135.7
130.5
130.5
128.2
125.2
125.2
117.8
117.6
11.4
CDCl$_3$
O
F
N
Cl
O
9b
1.4e+06
1.2e+06
1.0e+06
8.0e+05
6.0e+05
4.0e+05
2.0e+05
0.0e+00
170 160 150 140 130 120 110 100 90 80 70 60 50 40 30 20 10
ppm

^{1}H- and ^{13}C-NMR spectra of compound **10a**

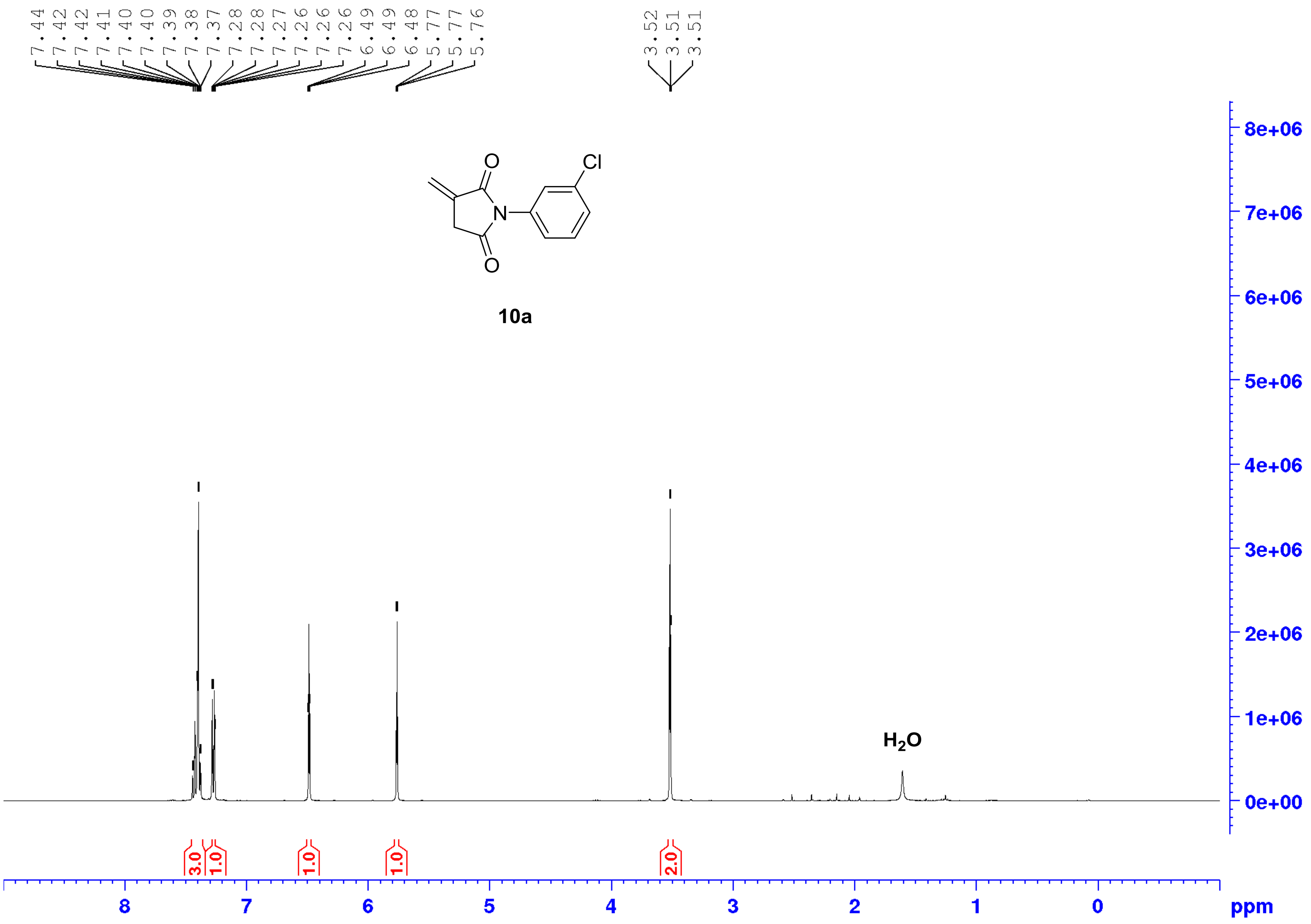

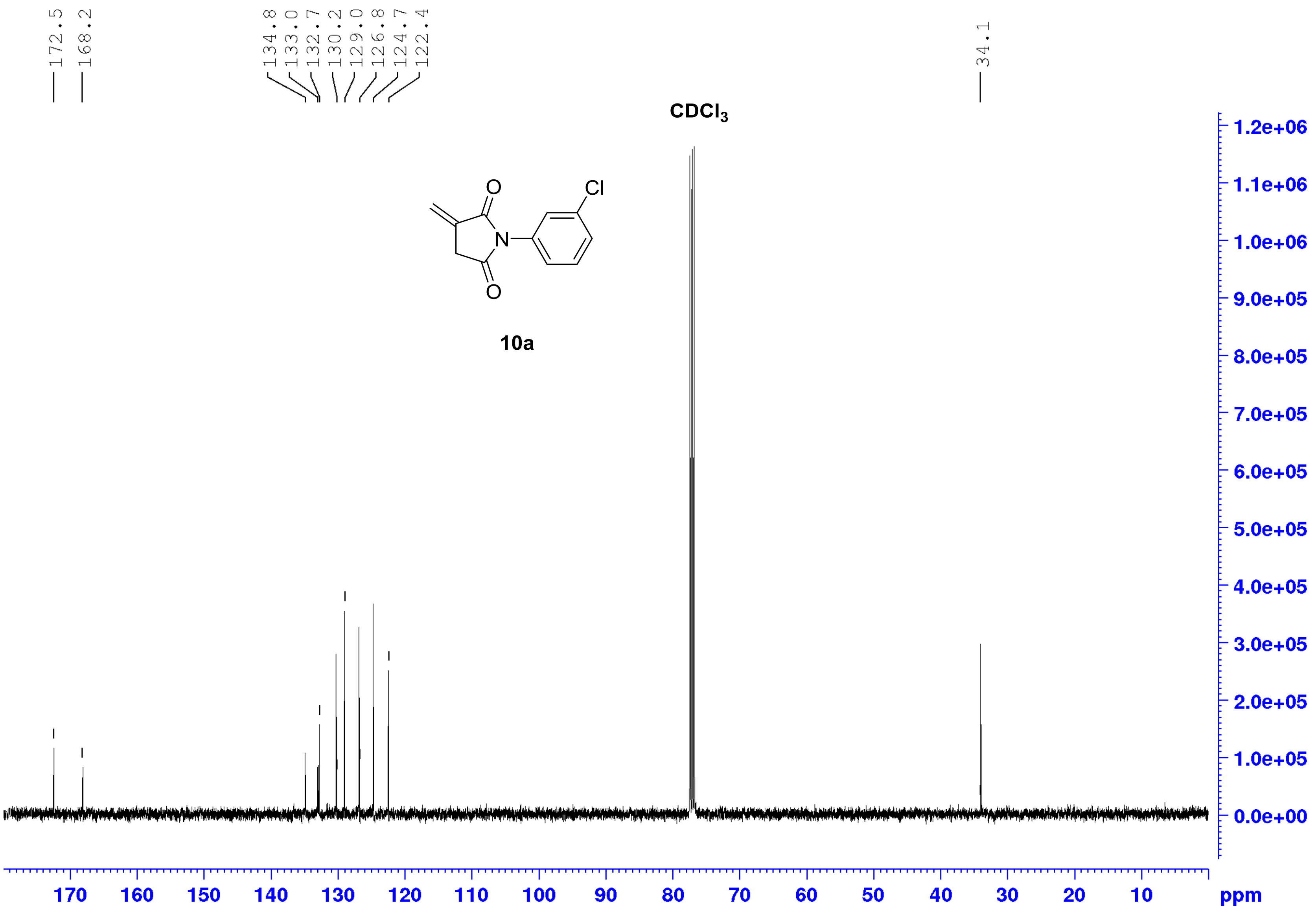

172.5
168.2
134.8
133.0
132.7
130.2
129.0
126.8
124.7
122.4
34.1
CDCl$_3$
Cl
O
N
O
10a
ppm

^{1}H- and ^{13}C-NMR spectra of compound **10b**

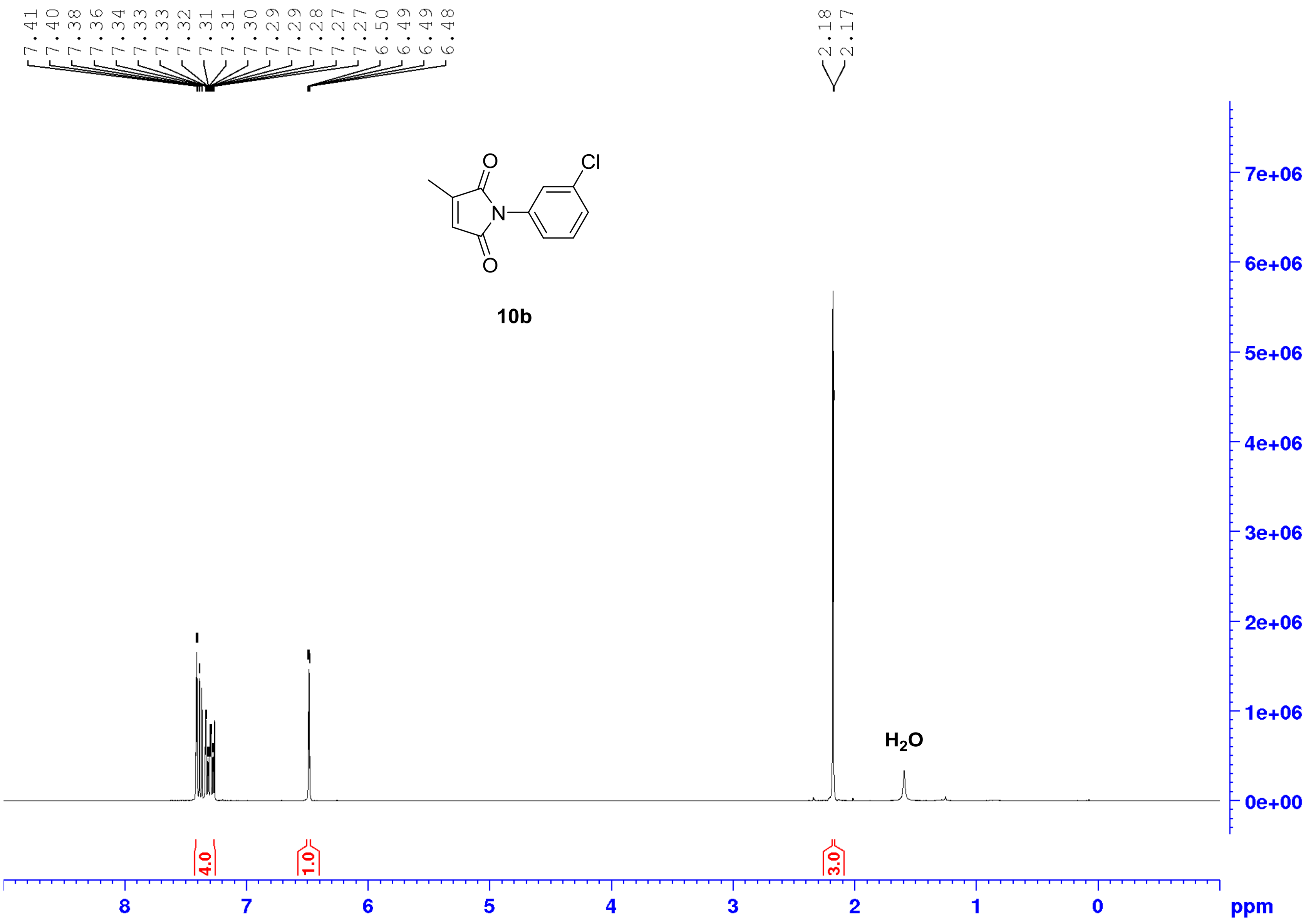

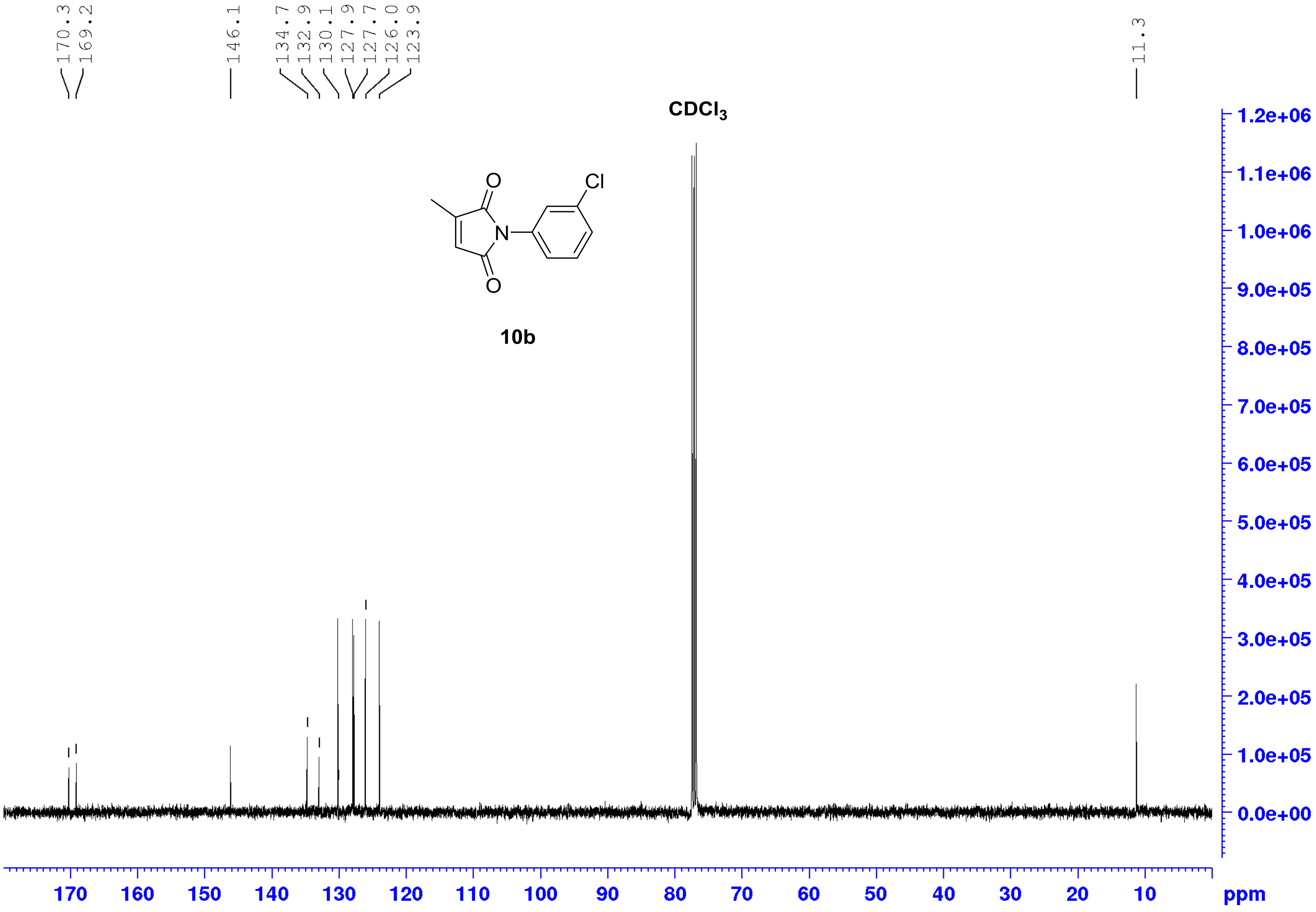
170.3
169.2
146.1
134.7
132.9
130.1
127.9
127.7
126.0
123.9
11.3
CDCl3
O
Cl
N
O
10b
1.2e+06
1.1e+06
1.0e+06
9.0e+05
8.0e+05
7.0e+05
6.0e+05
5.0e+05
4.0e+05
3.0e+05
2.0e+05
1.0e+05
0.0e+00
170
160
150
140
130
120
110
100
90
80
70
60
50
40
30
20
10
ppm

^1H- and ^{13}C-NMR spectra of compound **11a**

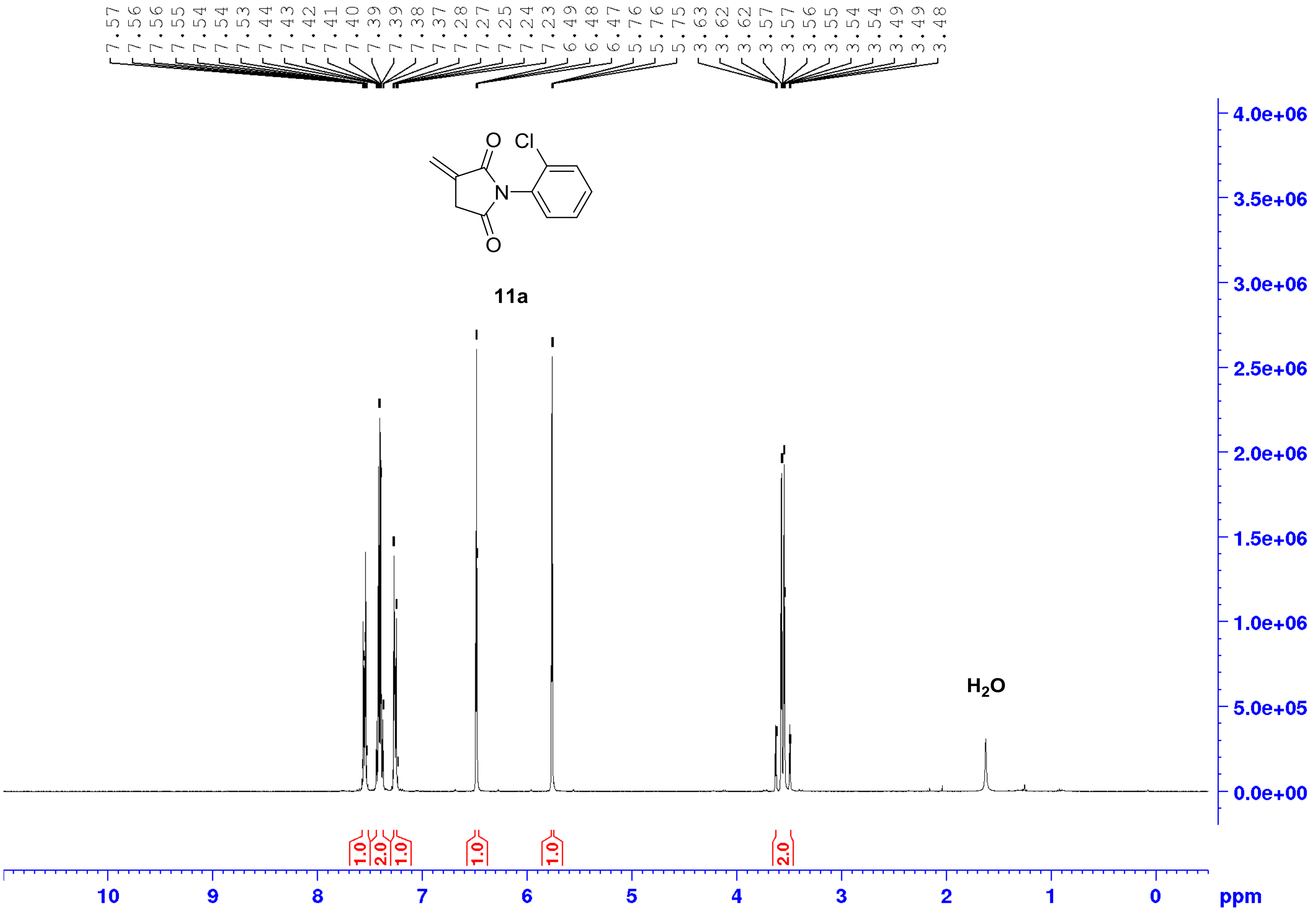

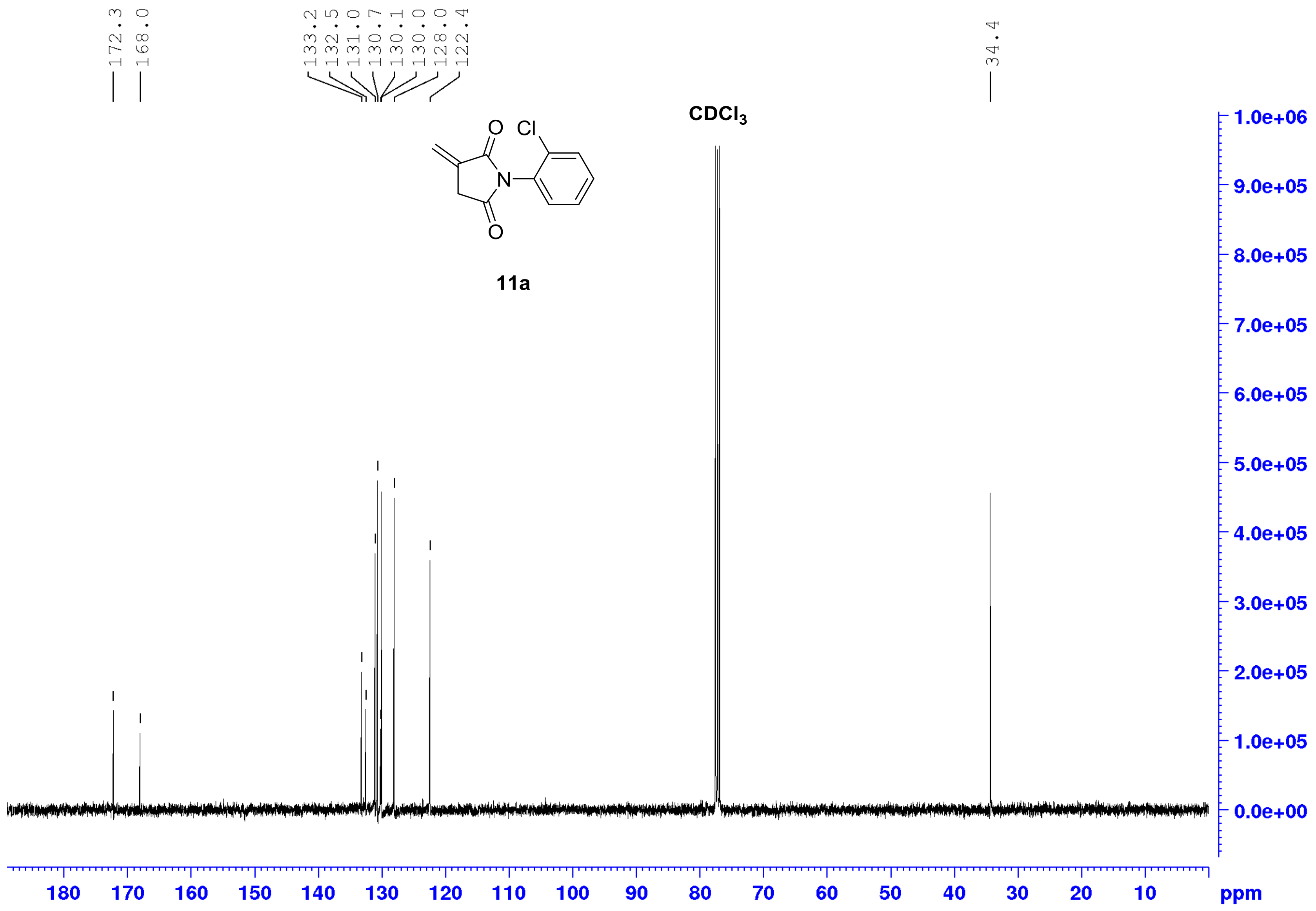
172.3
168.0
133.2
132.5
131.0
130.7
130.1
130.0
128.0
122.4
34.4
CDCl$_3$
O
Cl
N
O
11a
1.0e+06
9.0e+05
8.0e+05
7.0e+05
6.0e+05
5.0e+05
4.0e+05
3.0e+05
2.0e+05
1.0e+05
0.0e+00
180
170
160
150
140
130
120
110
100
90
80
70
60
50
40
30
20
10
ppm

^{1}H- and ^{13}C-NMR spectra of compound **11b**

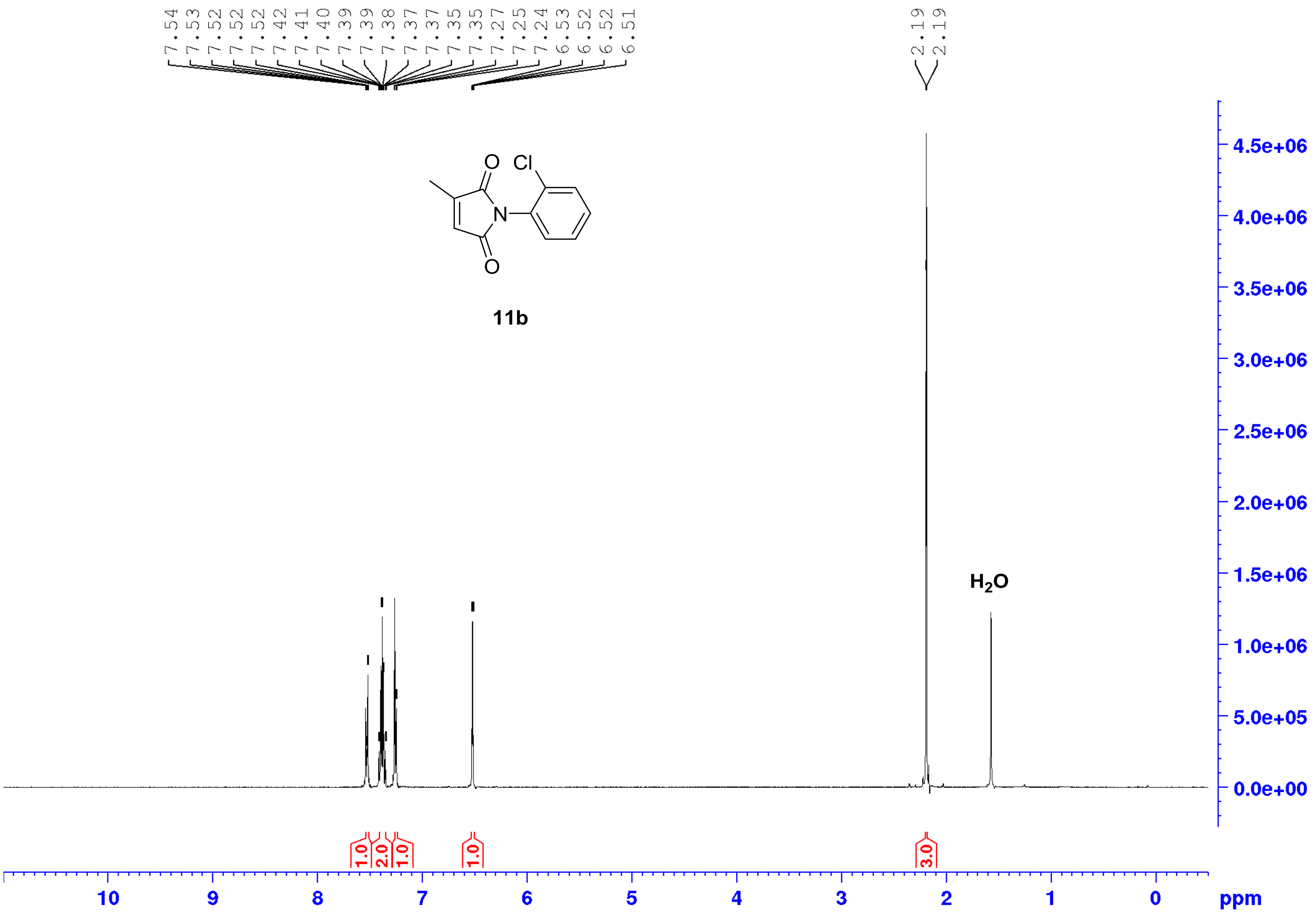

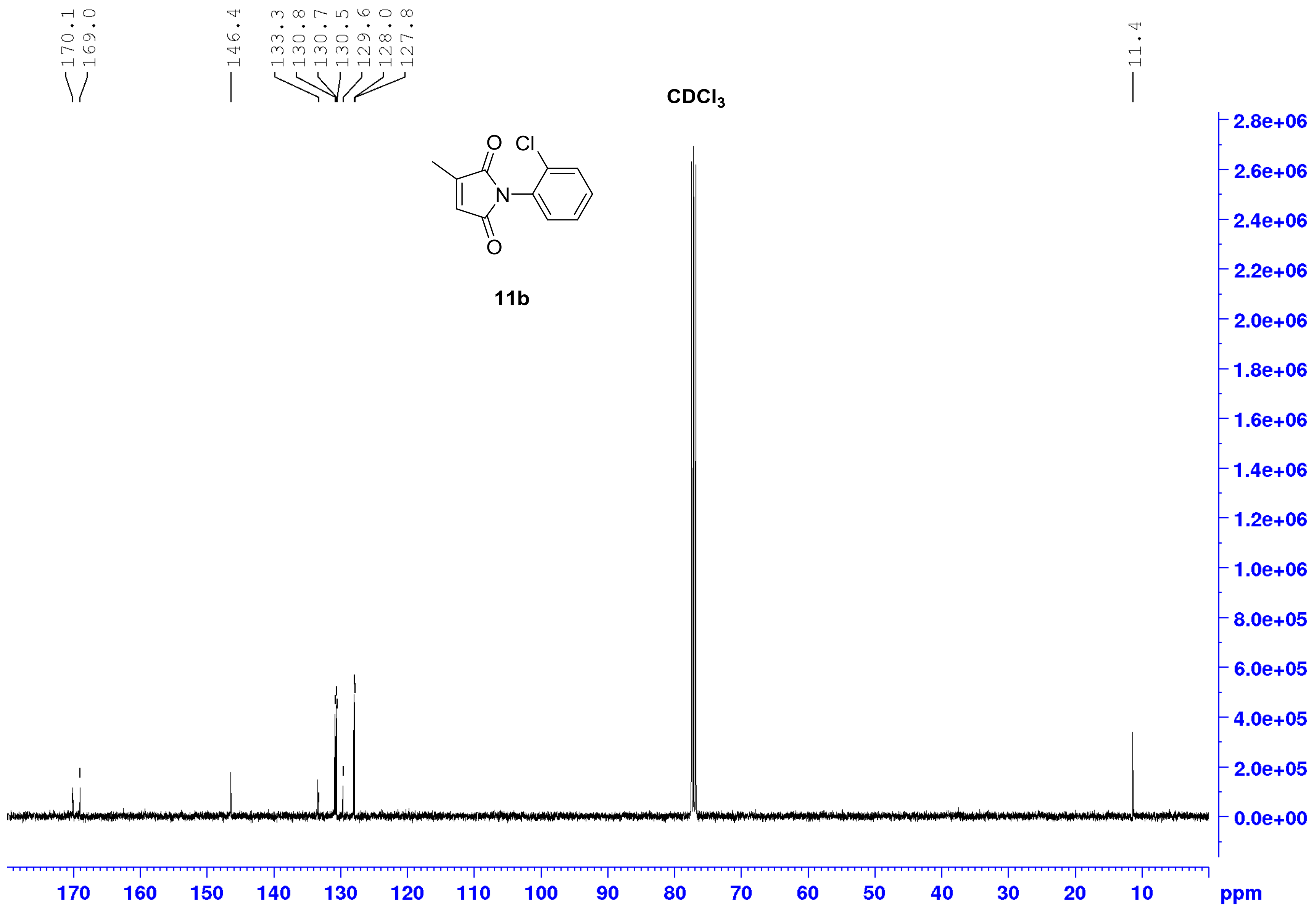
170.1
169.0
146.4
133.3
130.8
130.7
130.5
129.6
128.0
127.8
11.4
CDCl3
O
Cl
N
O
11b
2.8e+06
2.6e+06
2.4e+06
2.2e+06
2.0e+06
1.8e+06
1.6e+06
1.4e+06
1.2e+06
1.0e+06
8.0e+05
6.0e+05
4.0e+05
2.0e+05
0.0e+00
170 160 150 140 130 120 110 100 90 80 70 60 50 40 30 20 10
ppm

^{1}H- and ^{13}C-NMR spectra of compound **12a**

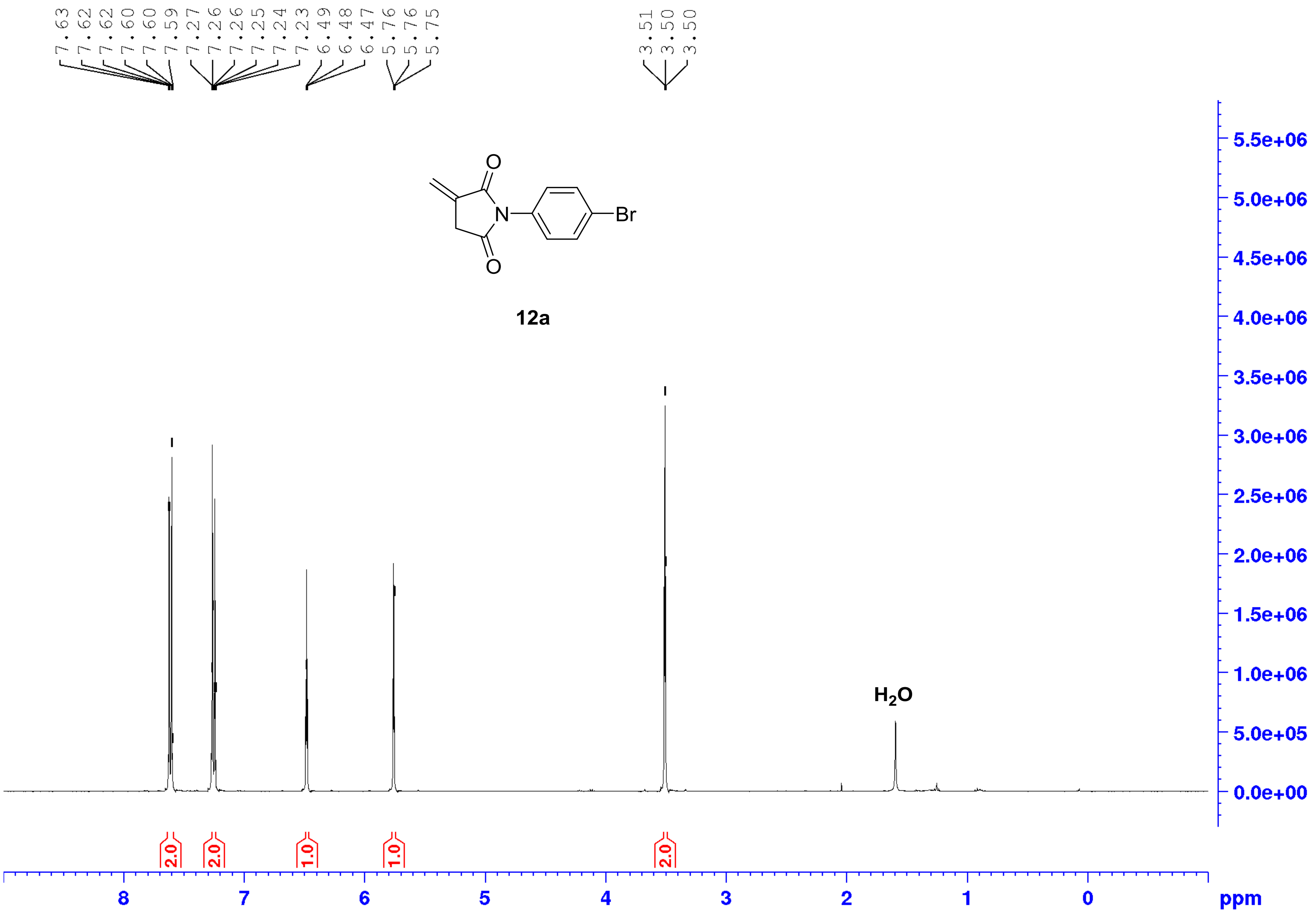

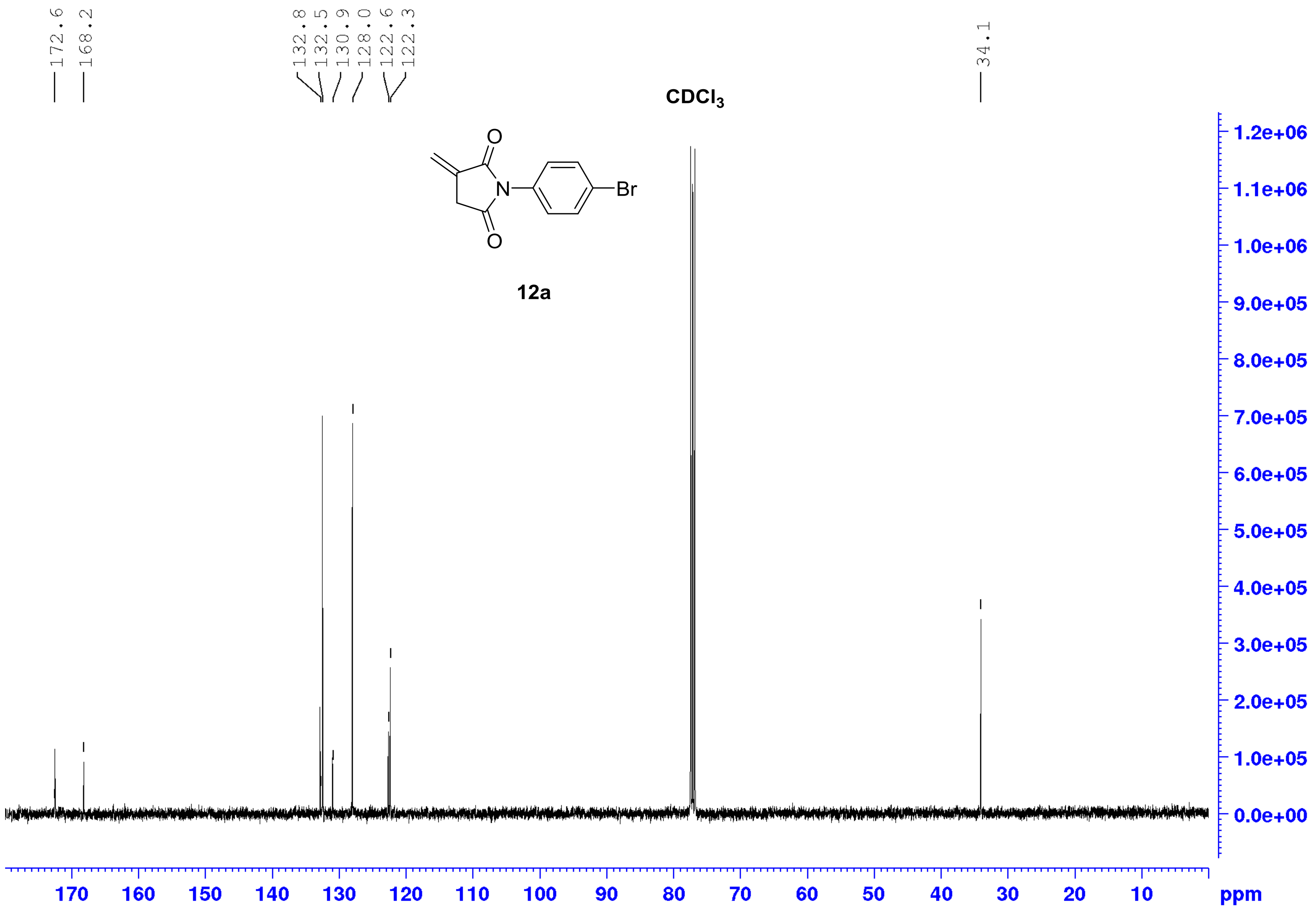

172.6
168.2
132.8
132.5
130.9
128.0
122.6
122.3
34.1
CDCl$_3$
O
N
Br
O
12a
1.2e+06
1.1e+06
1.0e+06
9.0e+05
8.0e+05
7.0e+05
6.0e+05
5.0e+05
4.0e+05
3.0e+05
2.0e+05
1.0e+05
0.0e+00
170 160 150 140 130 120 110 100 90 80 70 60 50 40 30 20 10
ppm

^{1}H- and ^{13}C-NMR spectra of compound **12b**

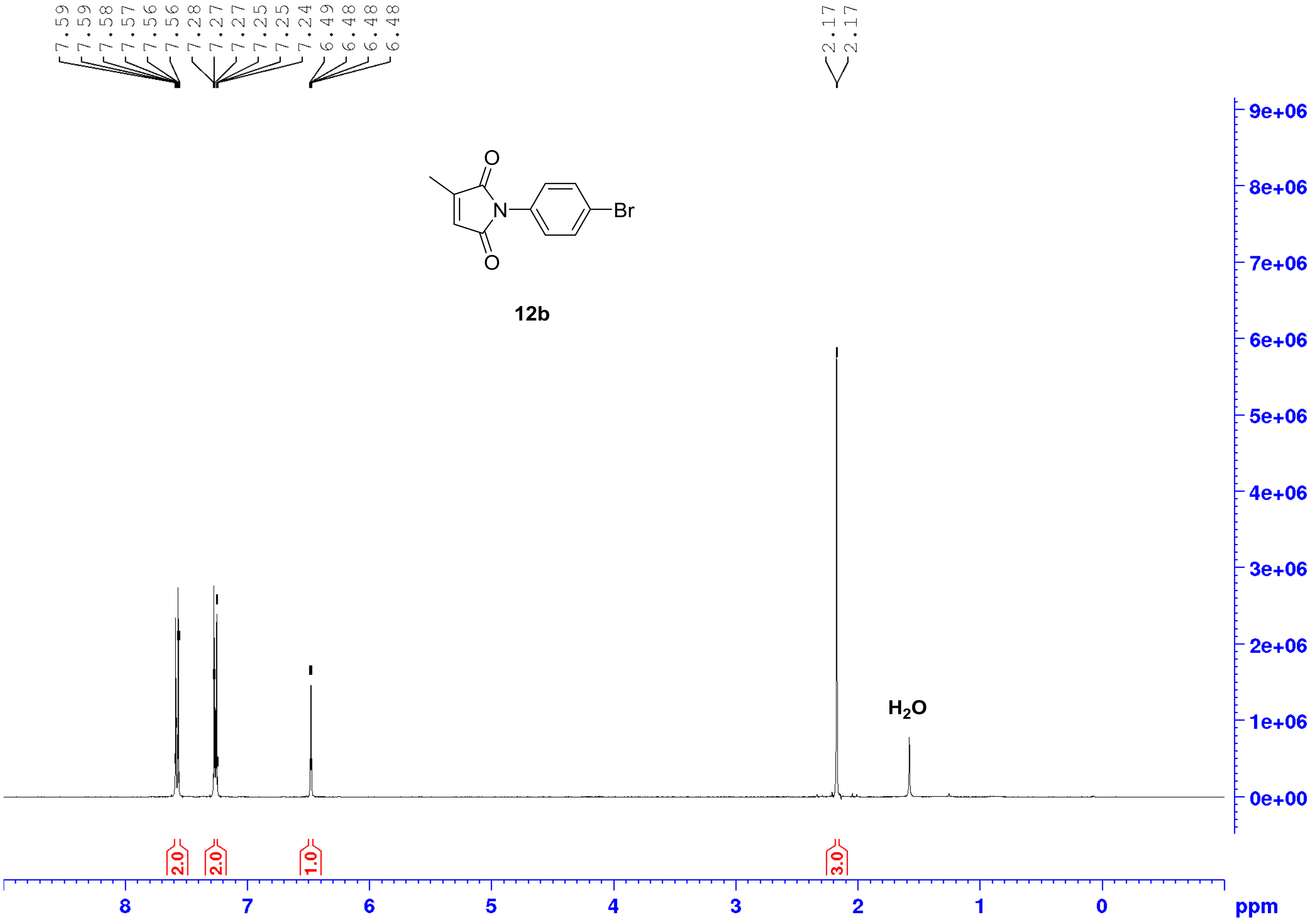

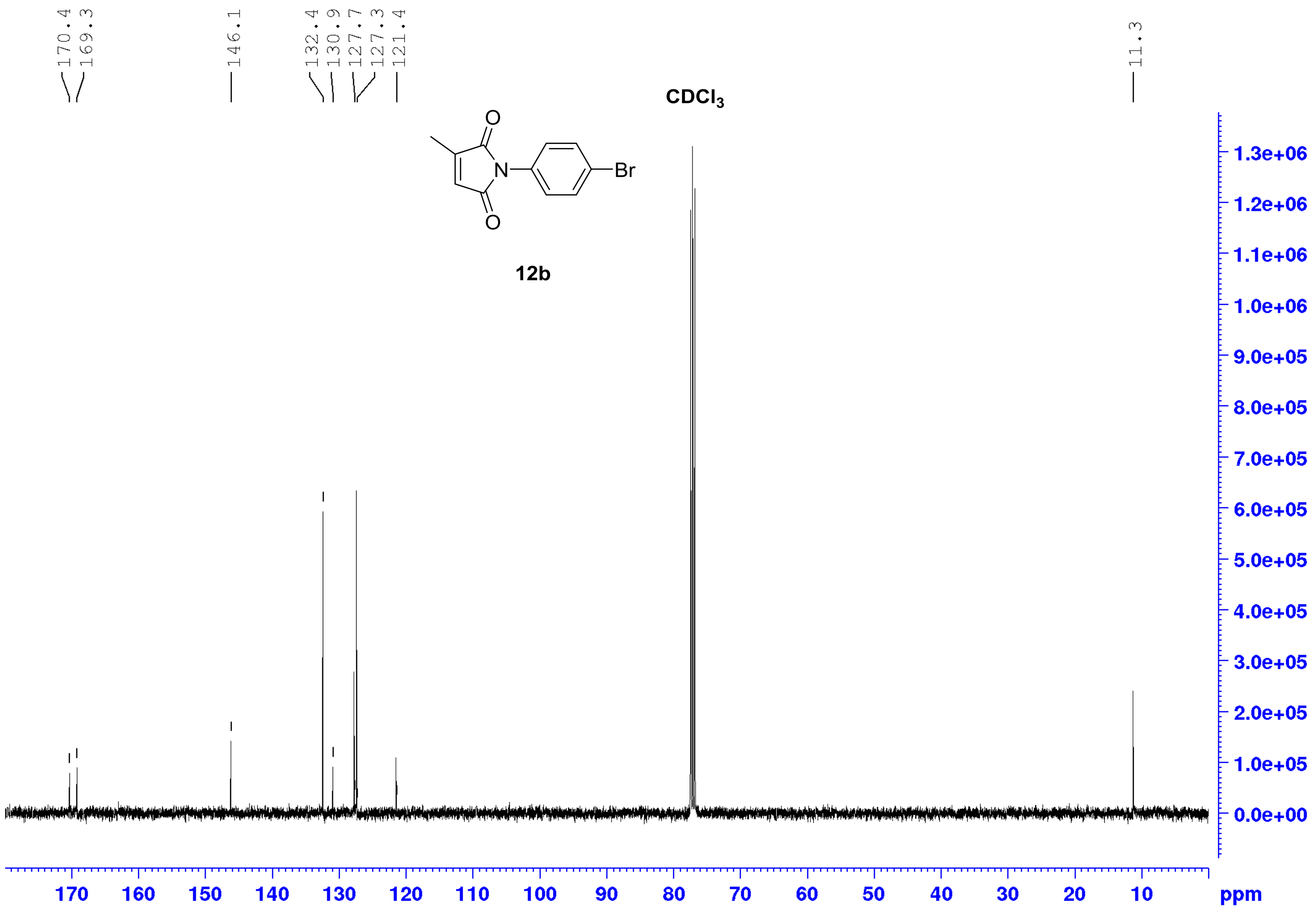

170.4
169.3
146.1
132.4
130.9
127.7
127.3
121.4
11.3
CDCl3
Br
N
O
O
12b
1.3e+06
1.2e+06
1.1e+06
1.0e+06
9.0e+05
8.0e+05
7.0e+05
6.0e+05
5.0e+05
4.0e+05
3.0e+05
2.0e+05
1.0e+05
0.0e+00
170
160
150
140
130
120
110
100
90
80
70
60
50
40
30
20
10
ppm

^{1}H- and ^{13}C-NMR spectra of compound **13a**

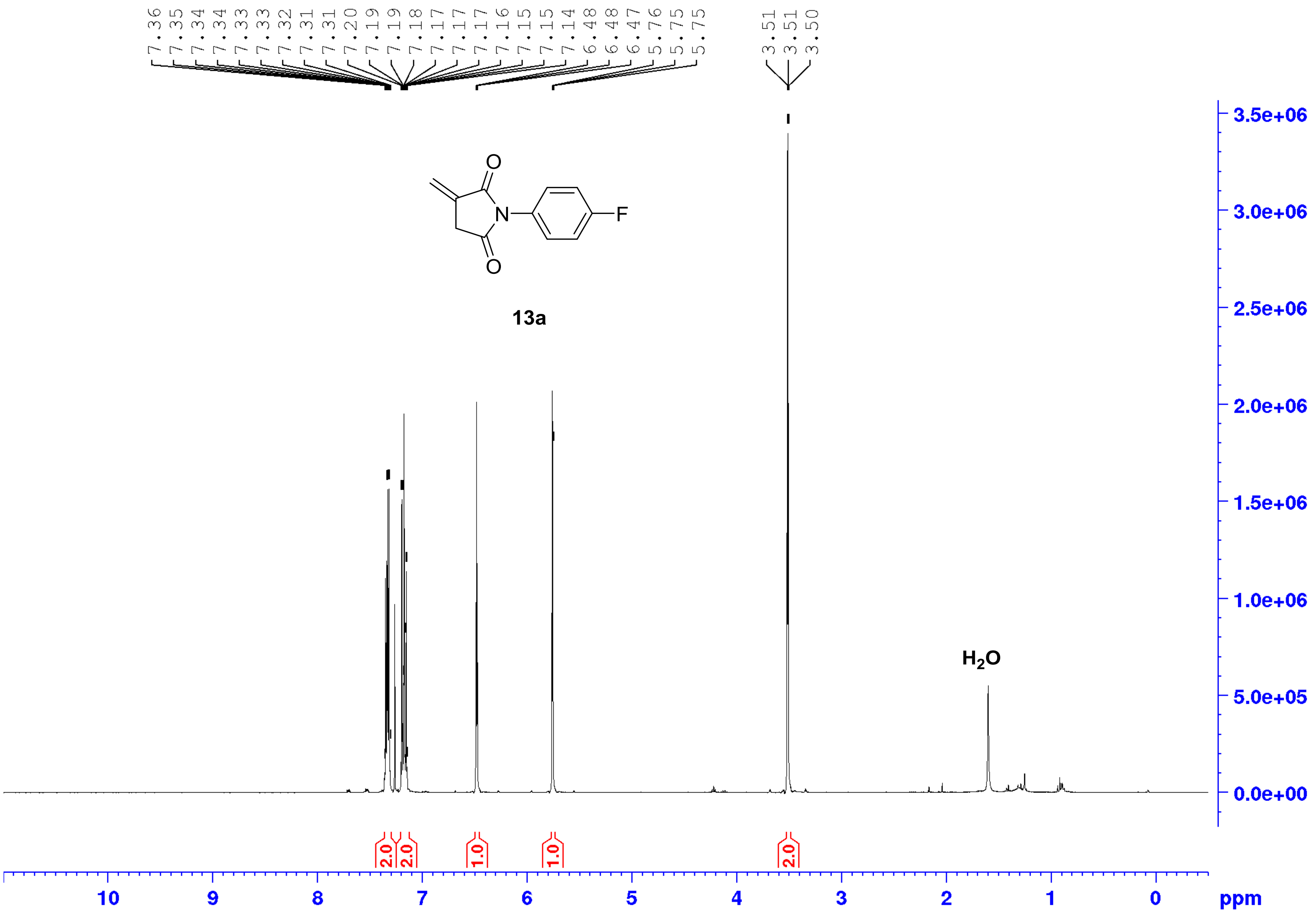

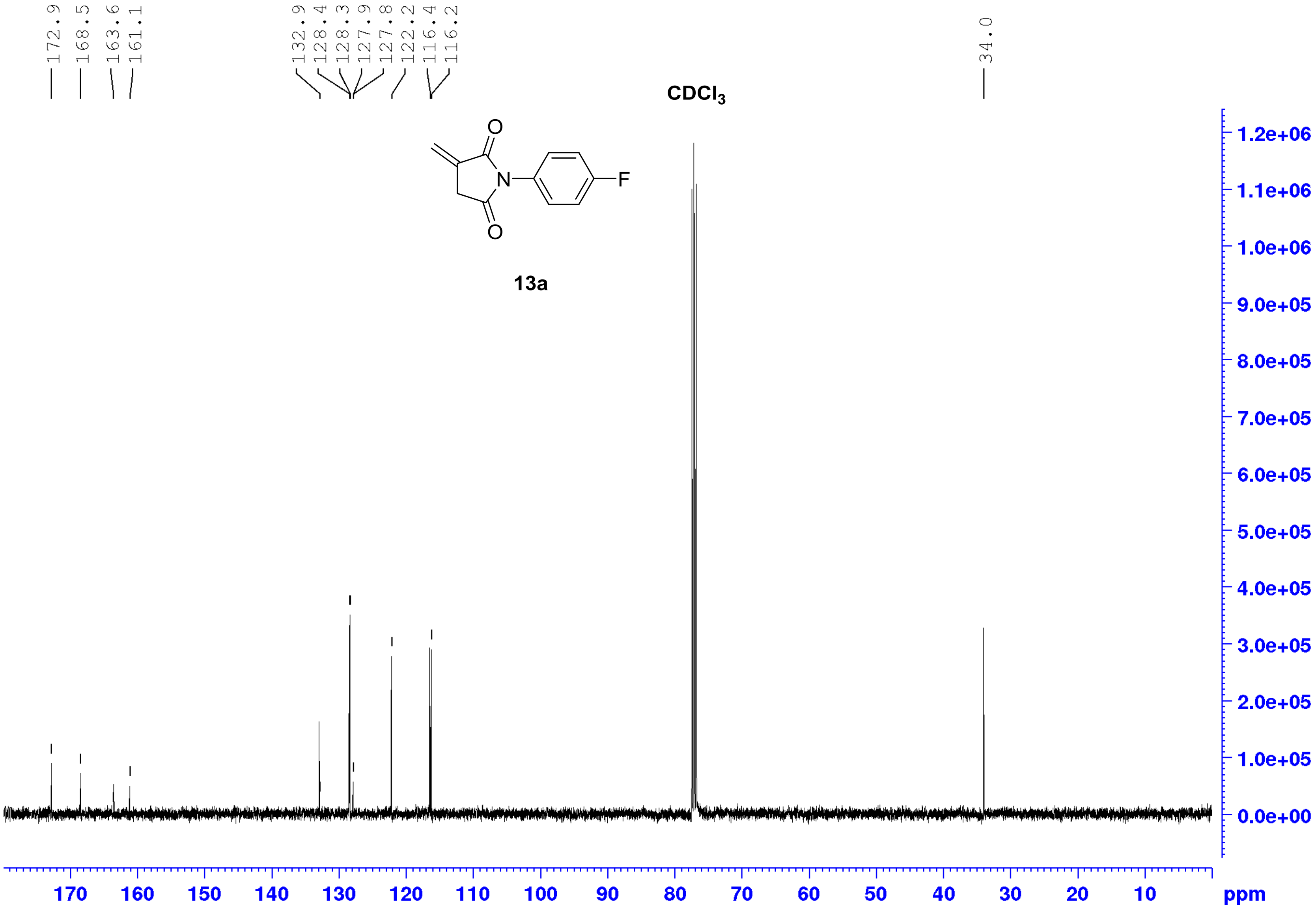
172.9
168.5
163.6
161.1
132.9
128.4
128.3
127.9
127.8
122.2
116.4
116.2
34.0
CDCl3
O
N
F
O
13a
1.2e+06
1.1e+06
1.0e+06
9.0e+05
8.0e+05
7.0e+05
6.0e+05
5.0e+05
4.0e+05
3.0e+05
2.0e+05
1.0e+05
0.0e+00
170 160 150 140 130 120 110 100 90 80 70 60 50 40 30 20 10
ppm

^{1}H- and ^{13}C-NMR spectra of compound **13b**

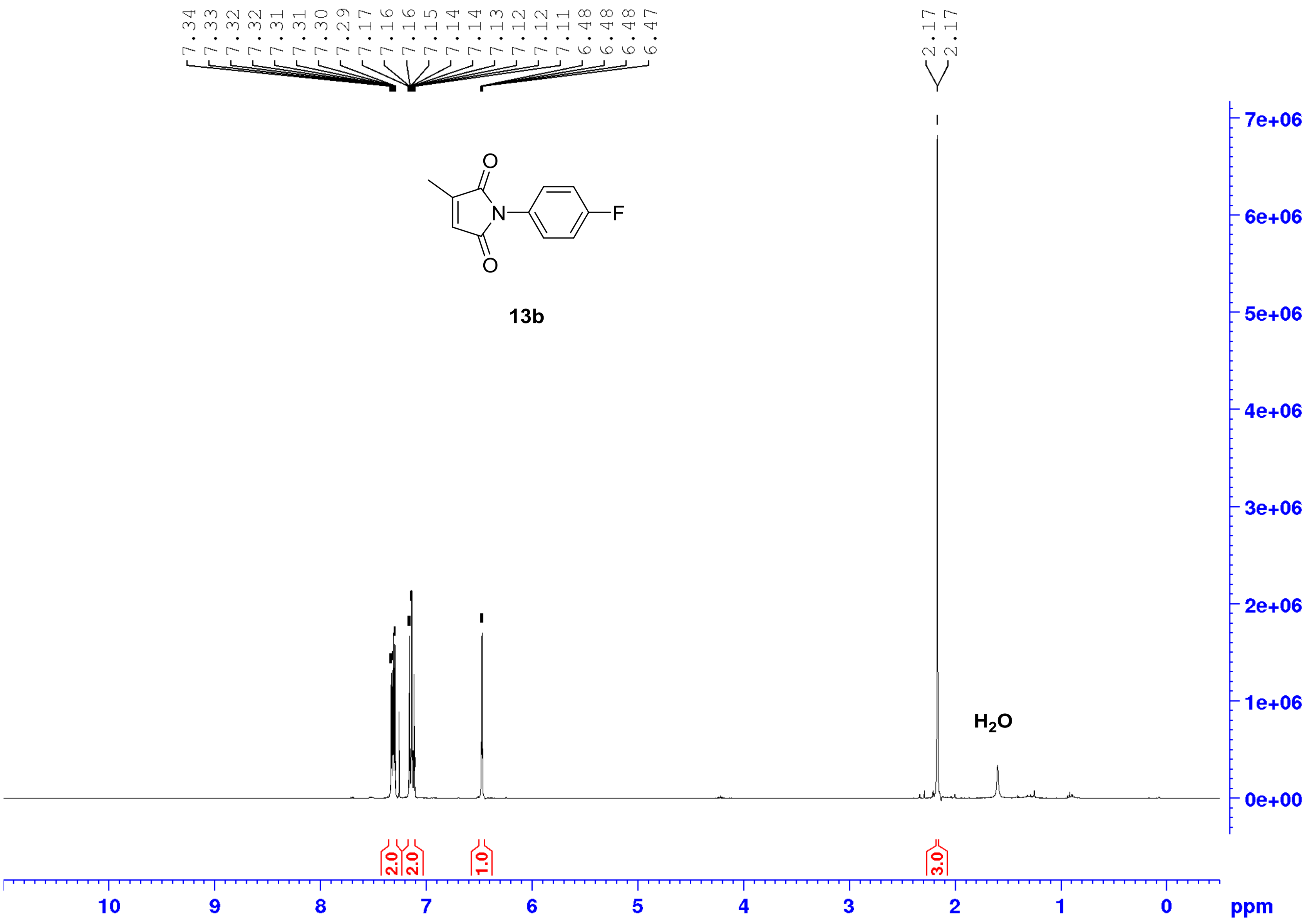

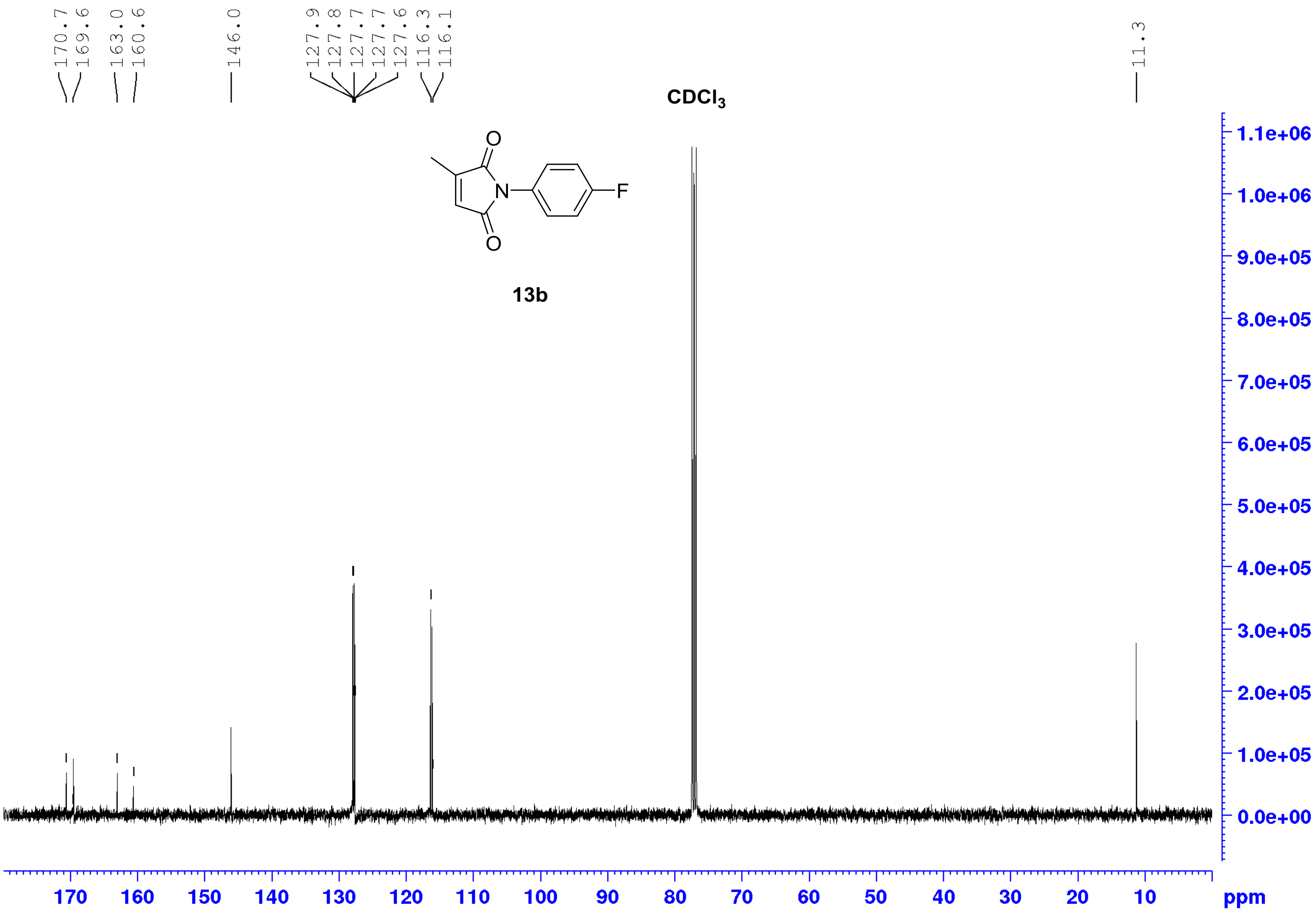
170.7
169.6
163.0
160.6
146.0
127.9
127.8
127.7
127.7
127.6
116.3
116.1
11.3
CDCl3
O
N
F
O
13b
1.1e+06
1.0e+06
9.0e+05
8.0e+05
7.0e+05
6.0e+05
5.0e+05
4.0e+05
3.0e+05
2.0e+05
1.0e+05
0.0e+00
170
160
150
140
130
120
110
100
90
80
70
60
50
40
30
20
10
ppm

^{1}H- and ^{13}C-NMR spectra of compound **14a**

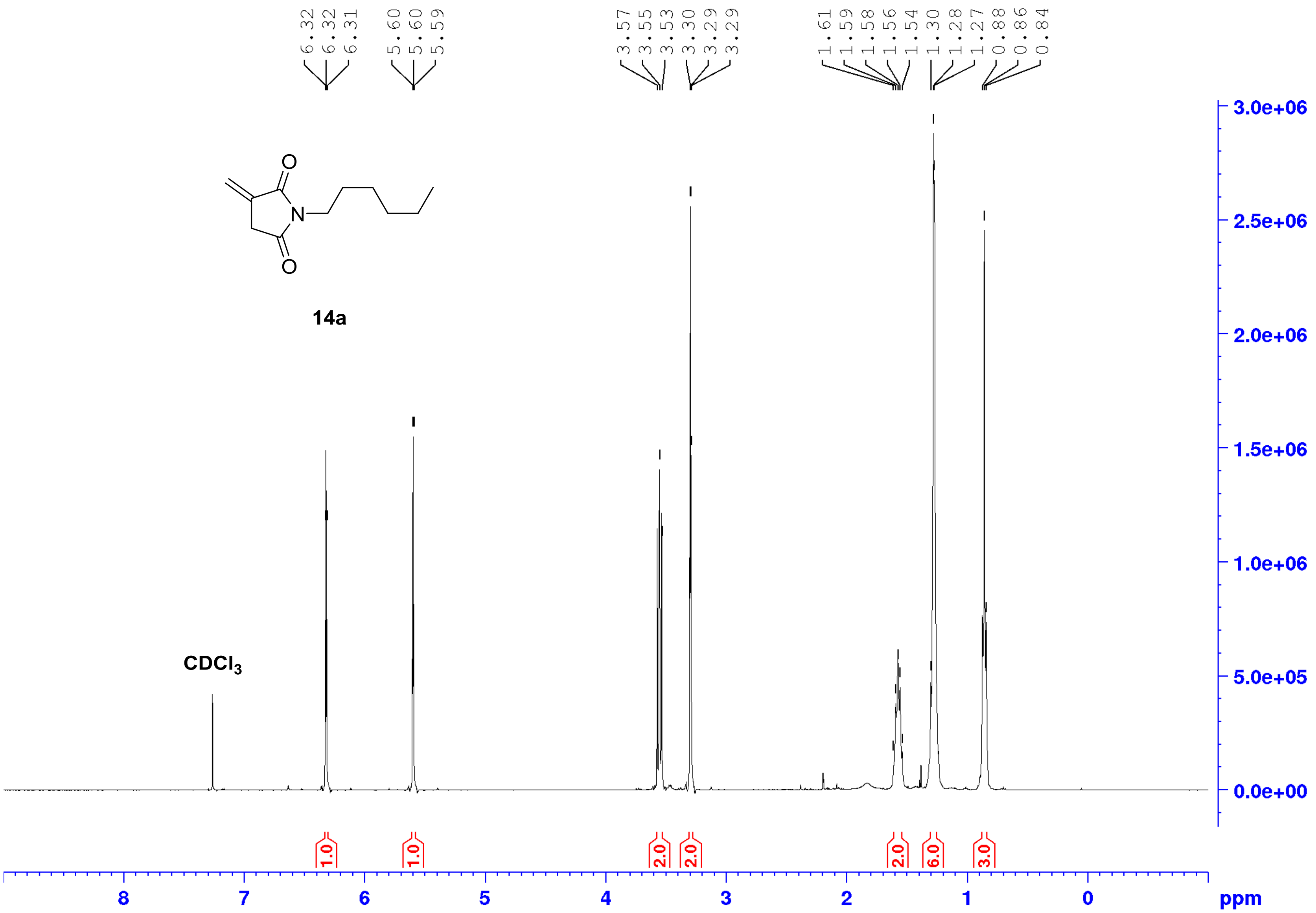

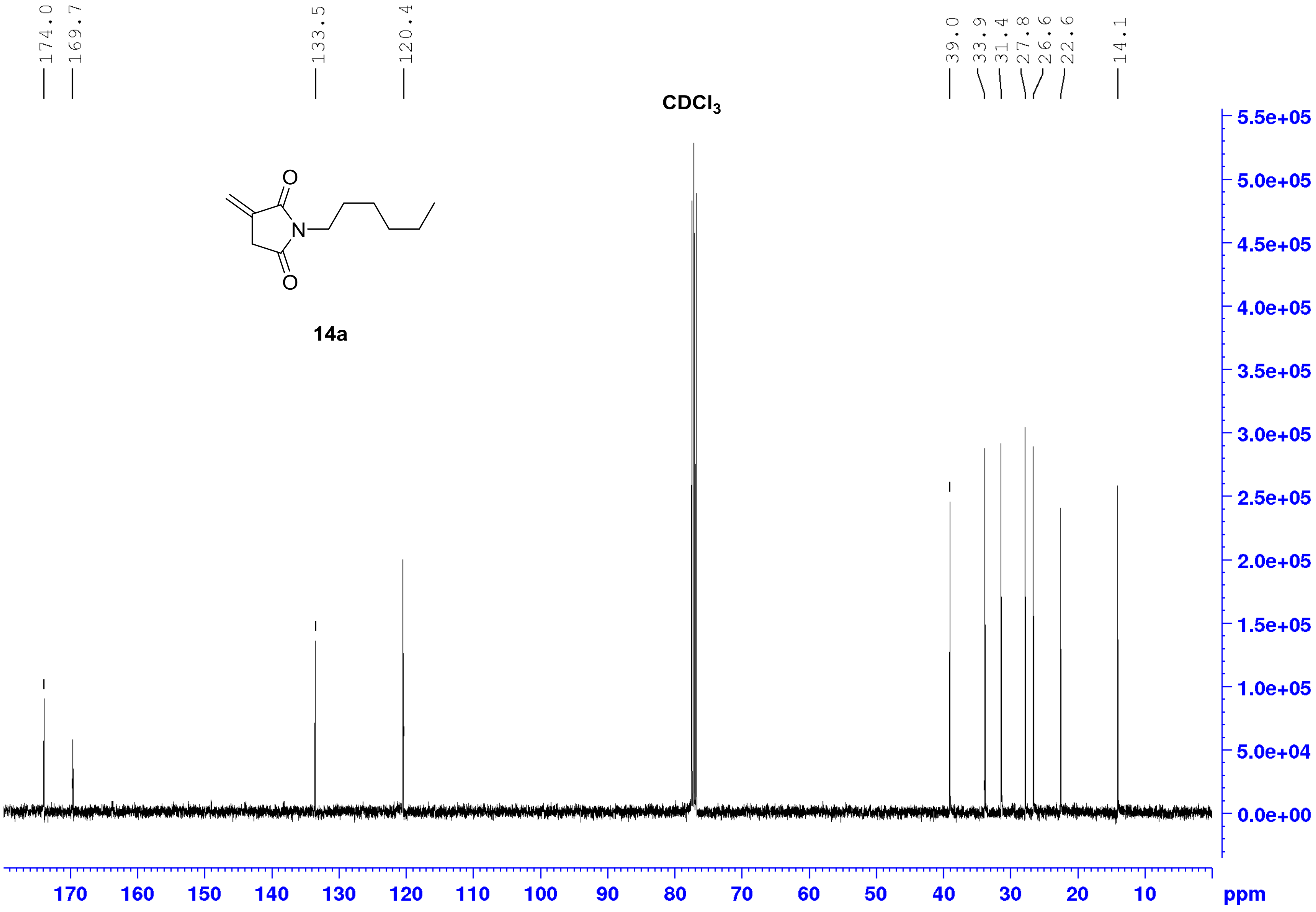

174.0
169.7
133.5
120.4
39.0
33.9
31.4
27.8
26.6
22.6
14.1
CDCl$_3$
O
N
O
14a
5.5e+05
5.0e+05
4.5e+05
4.0e+05
3.5e+05
3.0e+05
2.5e+05
2.0e+05
1.5e+05
1.0e+05
5.0e+04
0.0e+00
170 160 150 140 130 120 110 100 90 80 70 60 50 40 30 20 10
ppm

^{1}H- and ^{13}C-NMR spectra of compound **14b**

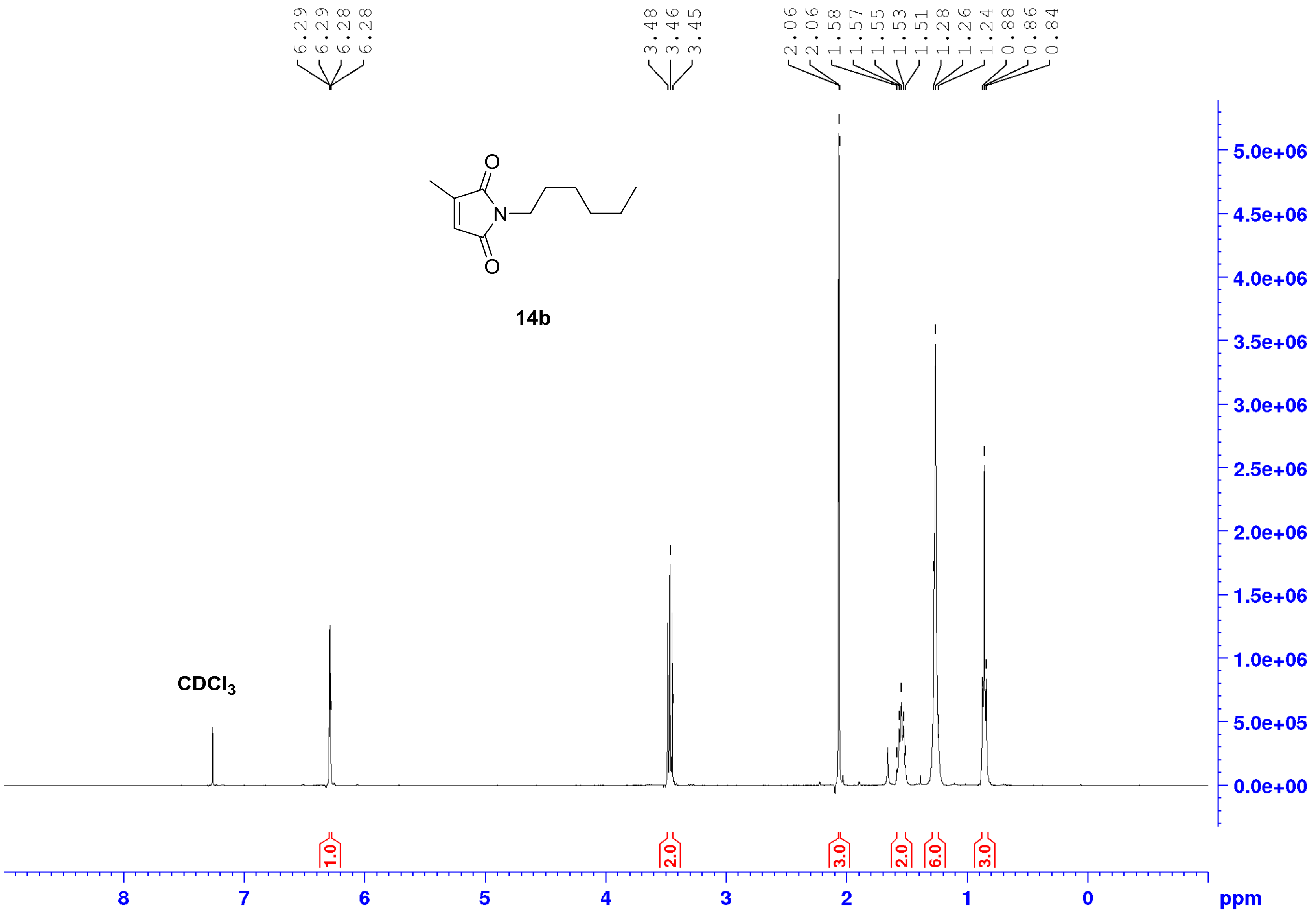

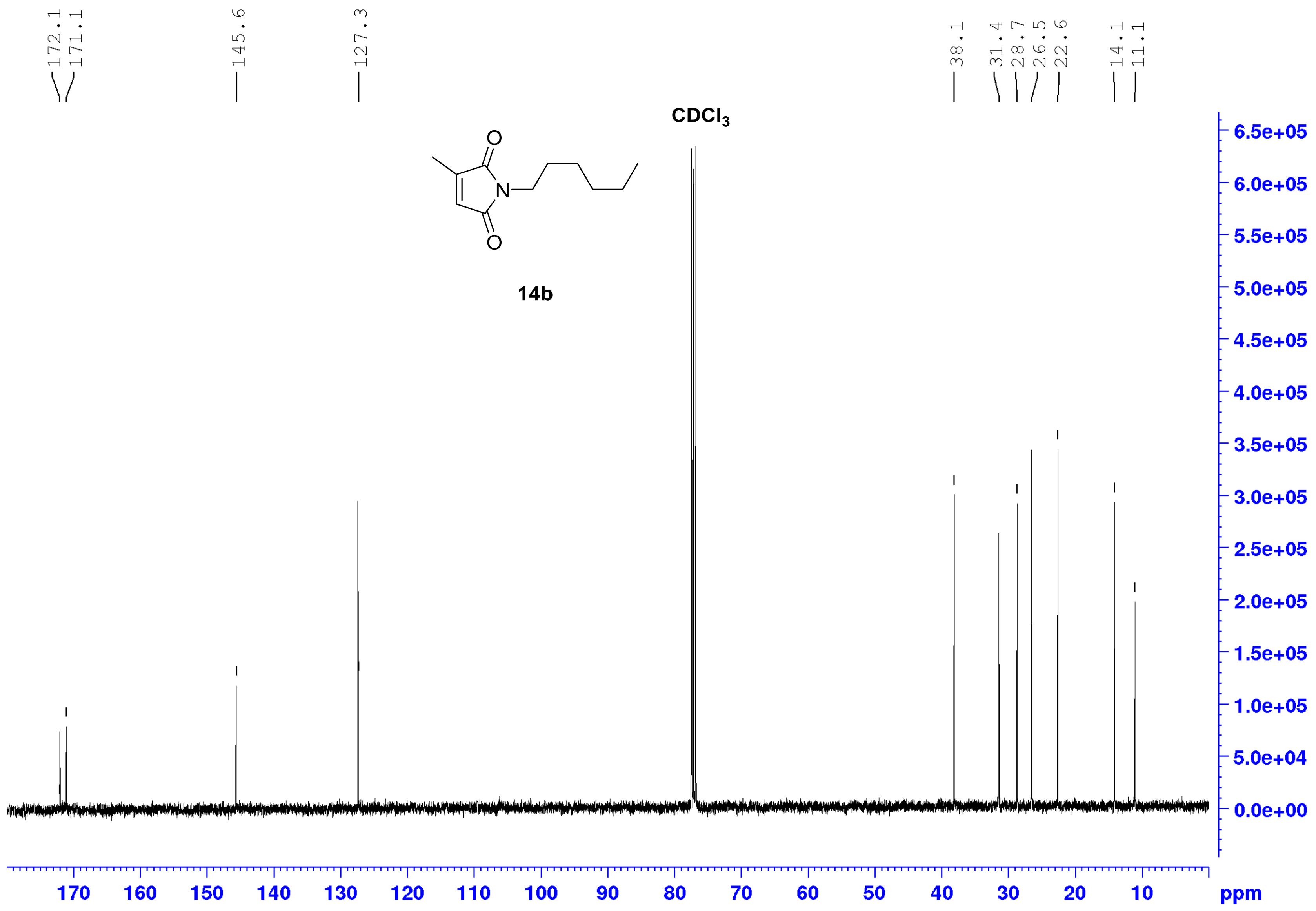
172.1
171.1
145.6
127.3
38.1
31.4
28.7
26.5
22.6
14.1
11.1
CDCl$_3$
O
N
O
14b
6.5e+05
6.0e+05
5.5e+05
5.0e+05
4.5e+05
4.0e+05
3.5e+05
3.0e+05
2.5e+05
2.0e+05
1.5e+05
1.0e+05
5.0e+04
0.0e+00
170
160
150
140
130
120
110
100
90
80
70
60
50
40
30
20
10
ppm

^{1}H- and ^{13}C-NMR spectra of compound **15a**

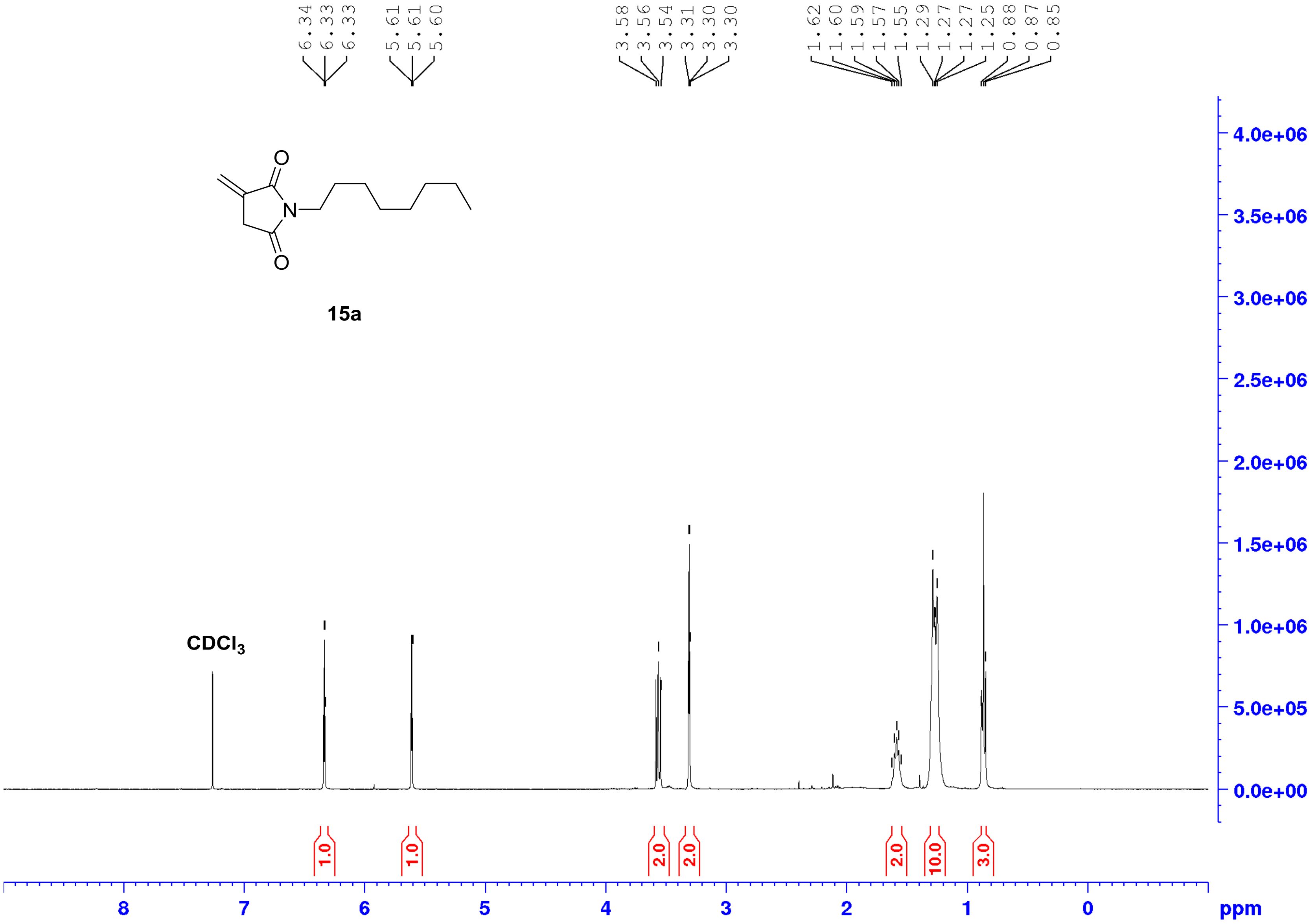

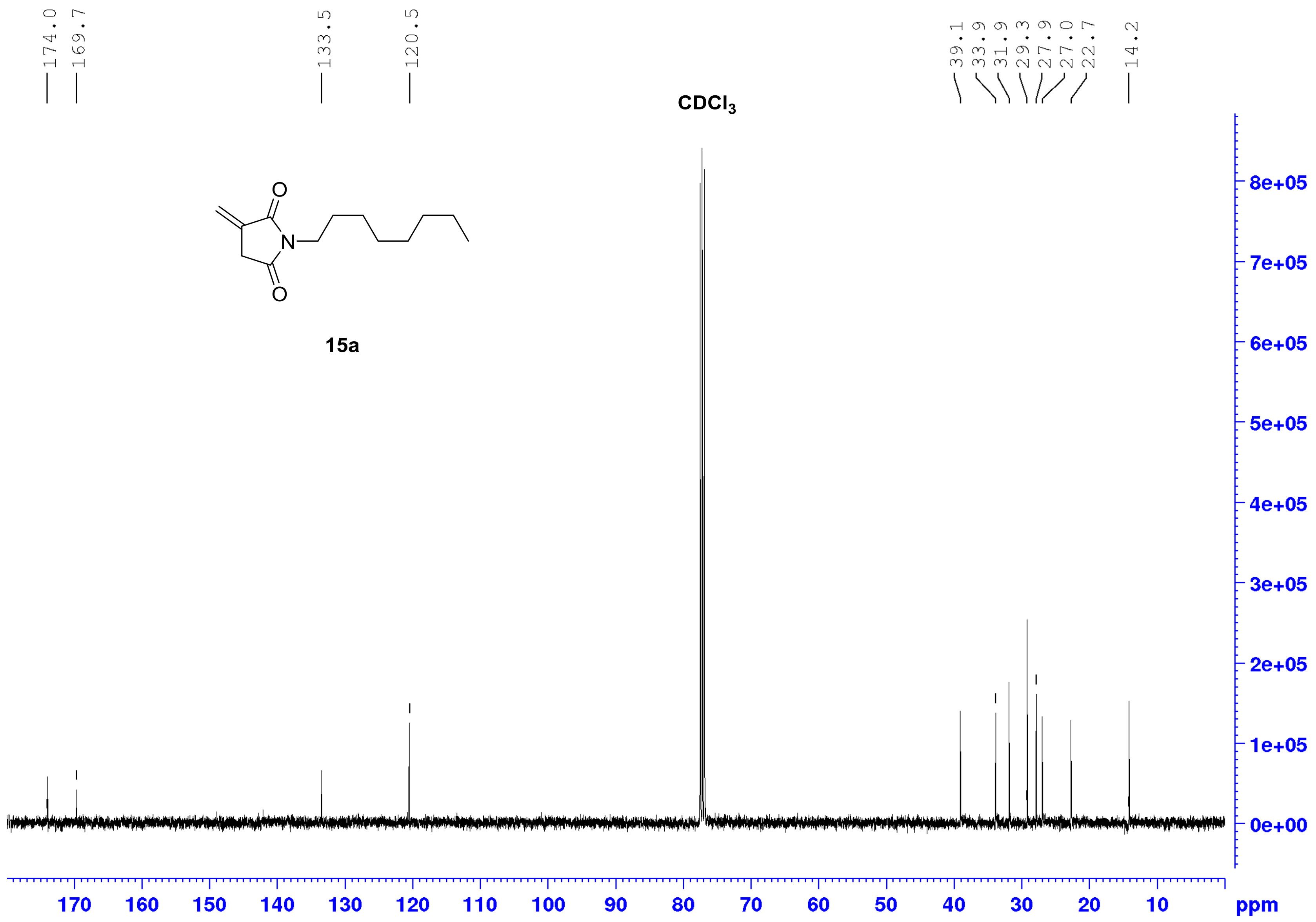

174.0
169.7
133.5
120.5
39.1
33.9
31.9
29.3
27.9
27.0
22.7
14.2
CDCl3
O
N
O
15a
8e+05
7e+05
6e+05
5e+05
4e+05
3e+05
2e+05
1e+05
0e+00
170
160
150
140
130
120
110
100
90
80
70
60
50
40
30
20
10
ppm

^{1}H- and ^{13}C-NMR spectra of compound **16a**

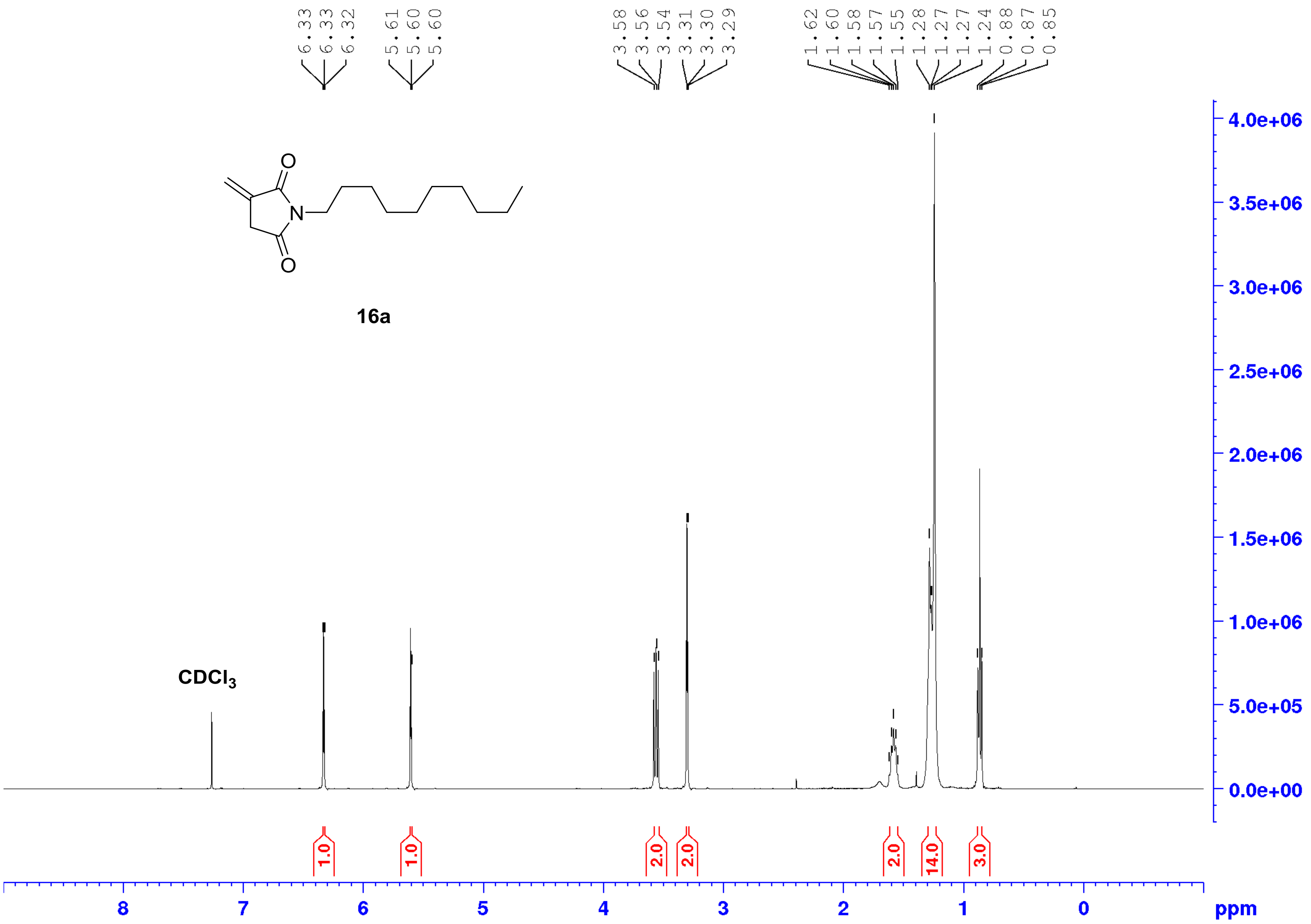

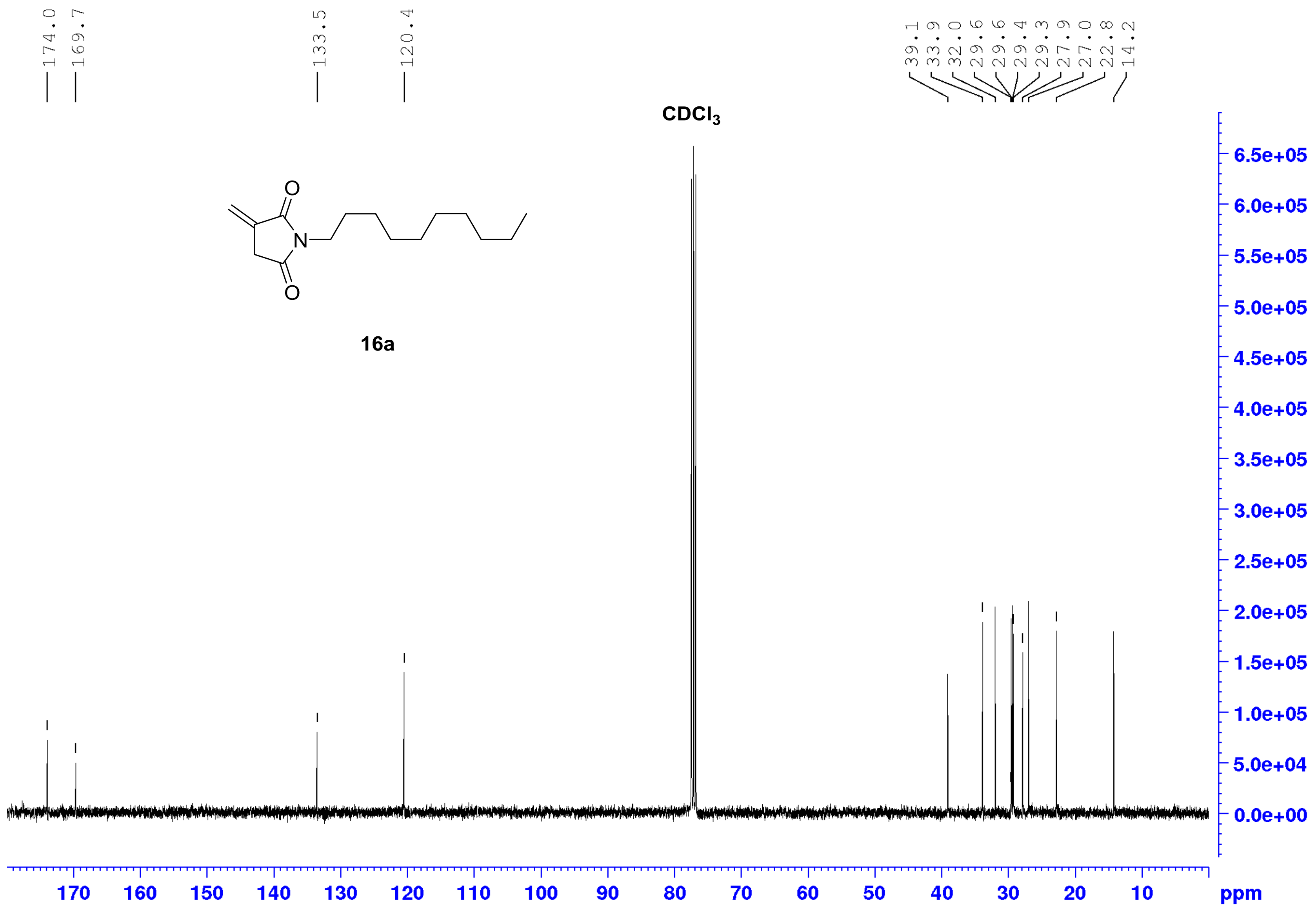

174.0
169.7
133.5
120.4
39.1
33.9
32.0
29.6
29.6
29.4
29.3
27.9
27.0
22.8
14.2
CDCl$_3$
O
N
O
16a
6.5e+05
6.0e+05
5.5e+05
5.0e+05
4.5e+05
4.0e+05
3.5e+05
3.0e+05
2.5e+05
2.0e+05
1.5e+05
1.0e+05
5.0e+04
0.0e+00
170
160
150
140
130
120
110
100
90
80
70
60
50
40
30
20
10
ppm

^{1}H- and ^{13}C-NMR spectra of compound **16b**

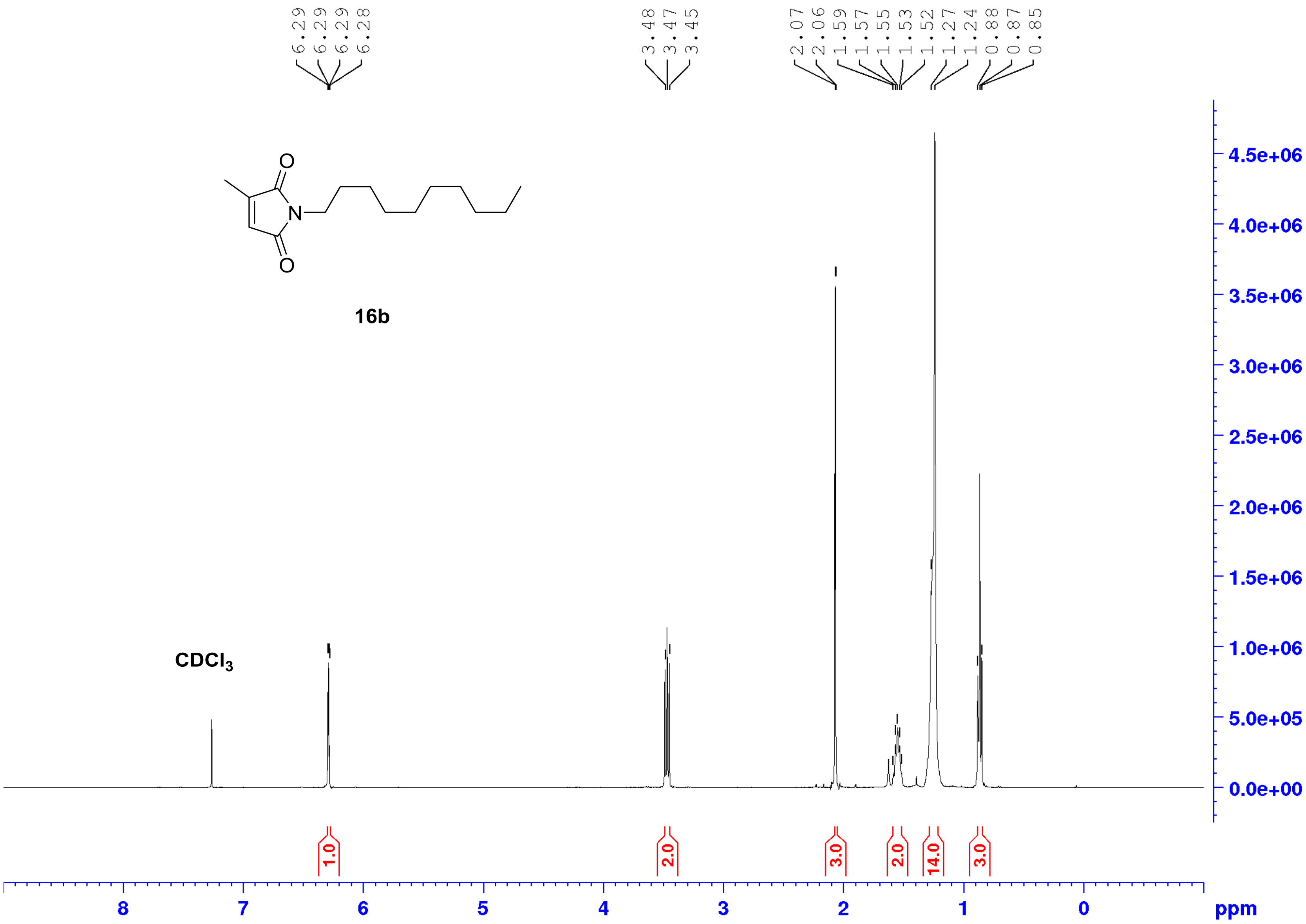

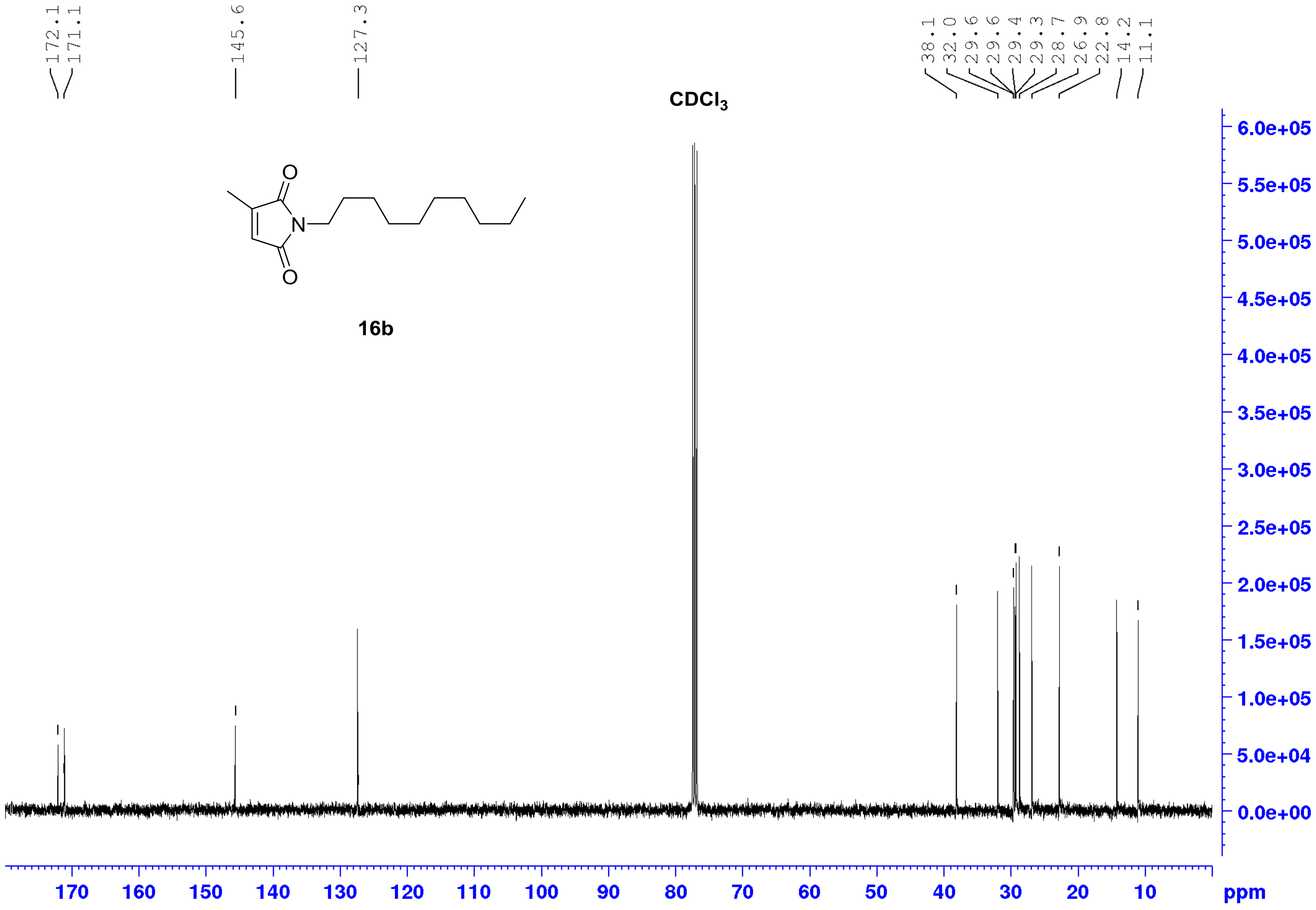
172.1
171.1
145.6
127.3
CDCl$_3$
38.1
32.0
29.6
29.6
29.4
29.3
28.7
26.9
22.8
14.2
11.1
16b
6.0e+05
5.5e+05
5.0e+05
4.5e+05
4.0e+05
3.5e+05
3.0e+05
2.5e+05
2.0e+05
1.5e+05
1.0e+05
5.0e+04
0.0e+00
170 160 150 140 130 120 110 100 90 80 70 60 50 40 30 20 10
ppm

^{1}H- and ^{13}C-NMR spectra of compound **17a**

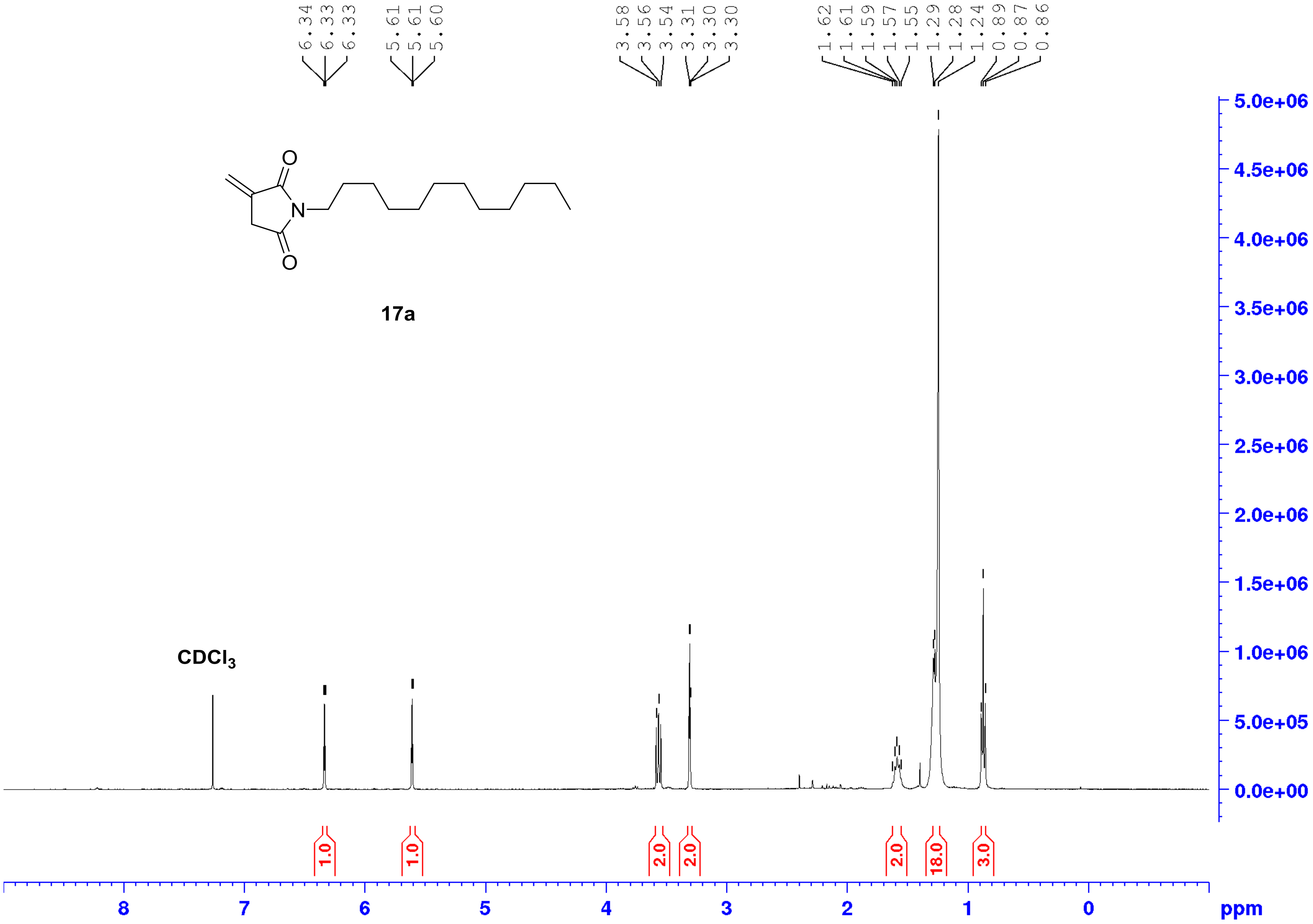

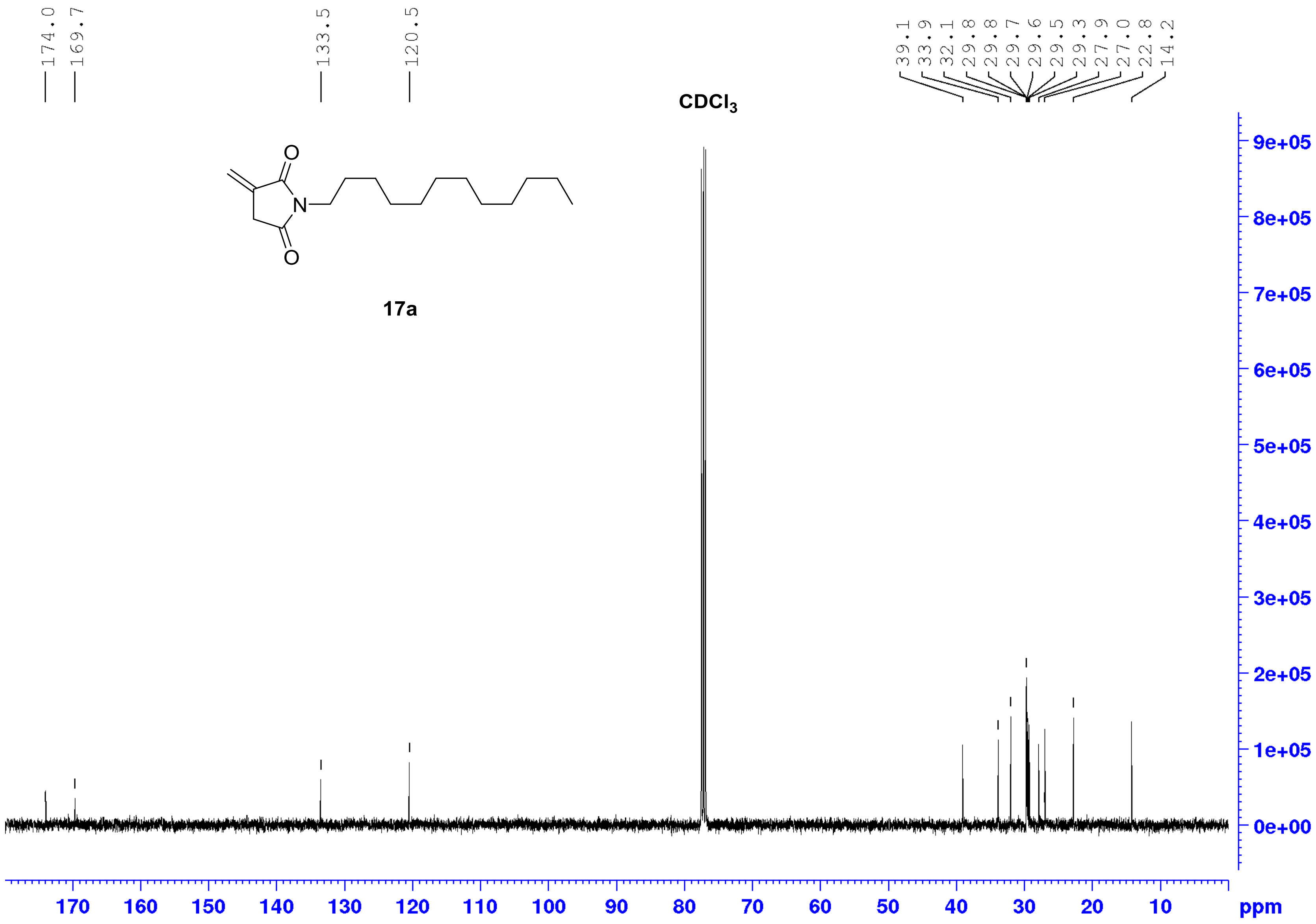
174.0
169.7
133.5
120.5
39.1
33.9
32.1
29.8
29.8
29.7
29.6
29.5
29.3
27.9
27.0
22.8
14.2
CDCl3
O
N
O
17a
9e+05
8e+05
7e+05
6e+05
5e+05
4e+05
3e+05
2e+05
1e+05
0e+00
170
160
150
140
130
120
110
100
90
80
70
60
50
40
30
20
10
ppm

^{1}H- and ^{13}C-NMR spectra of compound **17b**

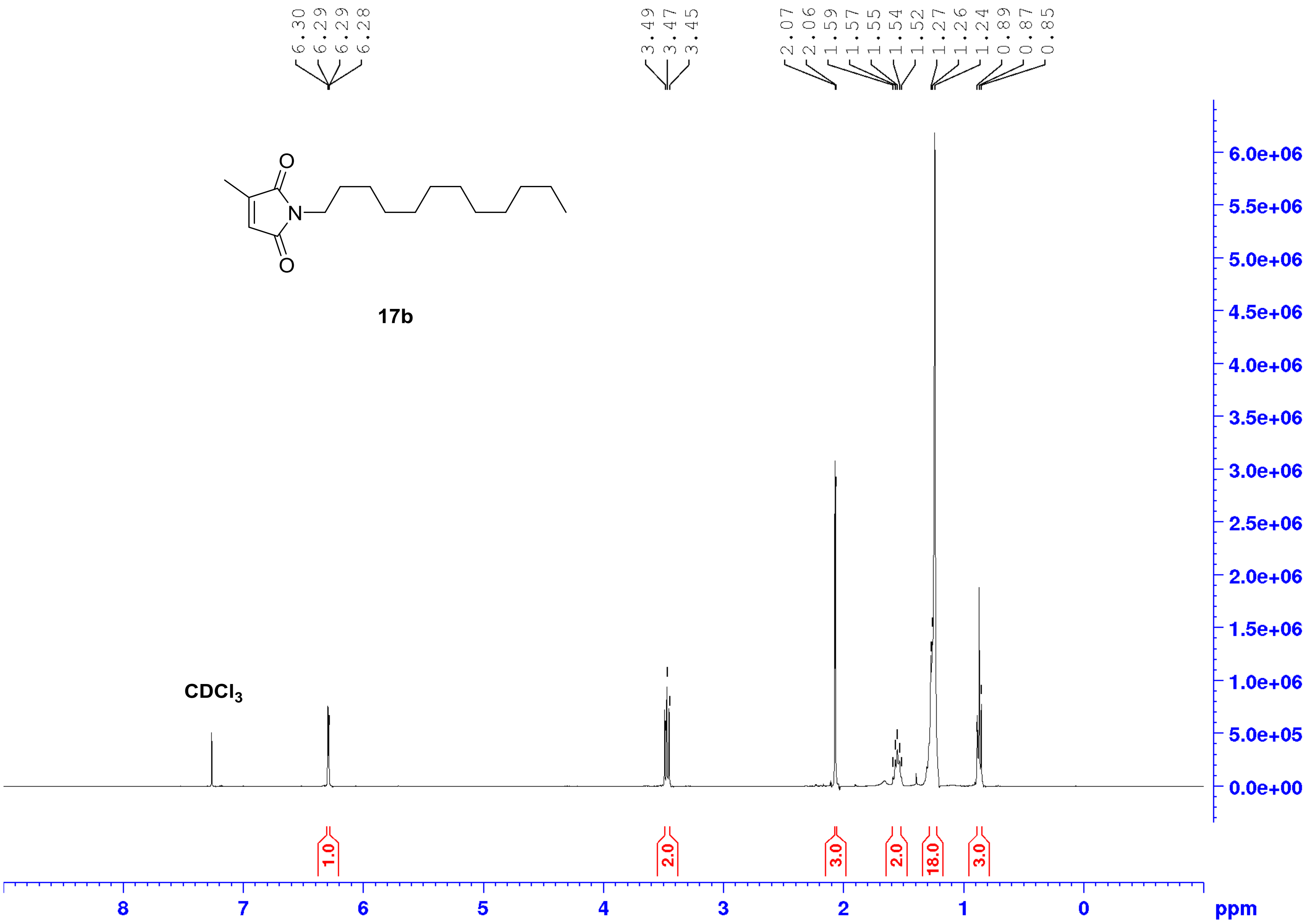

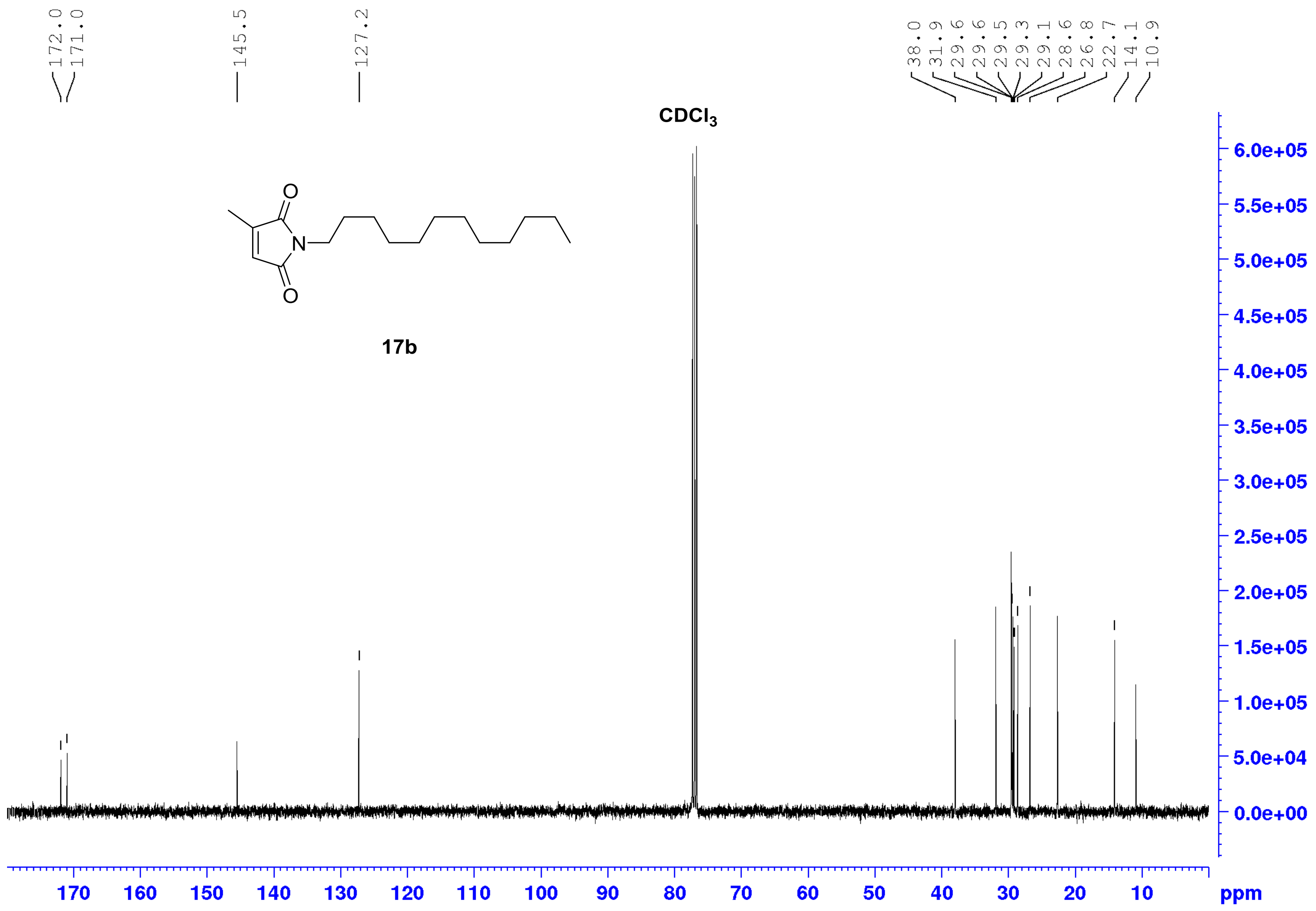
172.0
171.0
145.5
127.2
38.0
31.9
29.6
29.6
29.5
29.3
29.1
28.6
26.8
22.7
14.1
10.9
CDCl3
17b
6.0e+05
5.5e+05
5.0e+05
4.5e+05
4.0e+05
3.5e+05
3.0e+05
2.5e+05
2.0e+05
1.5e+05
1.0e+05
5.0e+04
0.0e+00
170 160 150 140 130 120 110 100 90 80 70 60 50 40 30 20 10
ppm

^{1}H- and ^{13}C-NMR spectra of compound **18a**

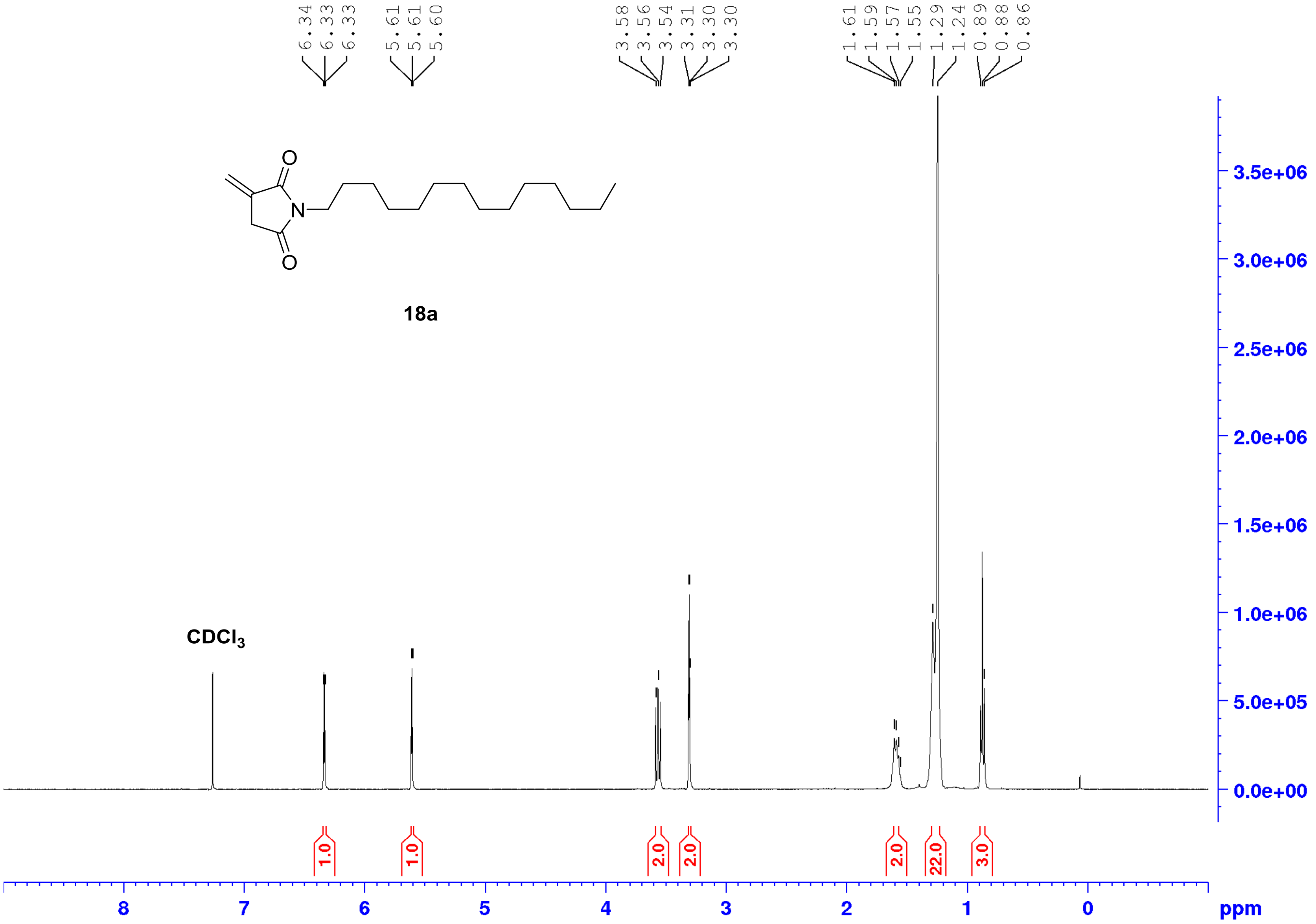

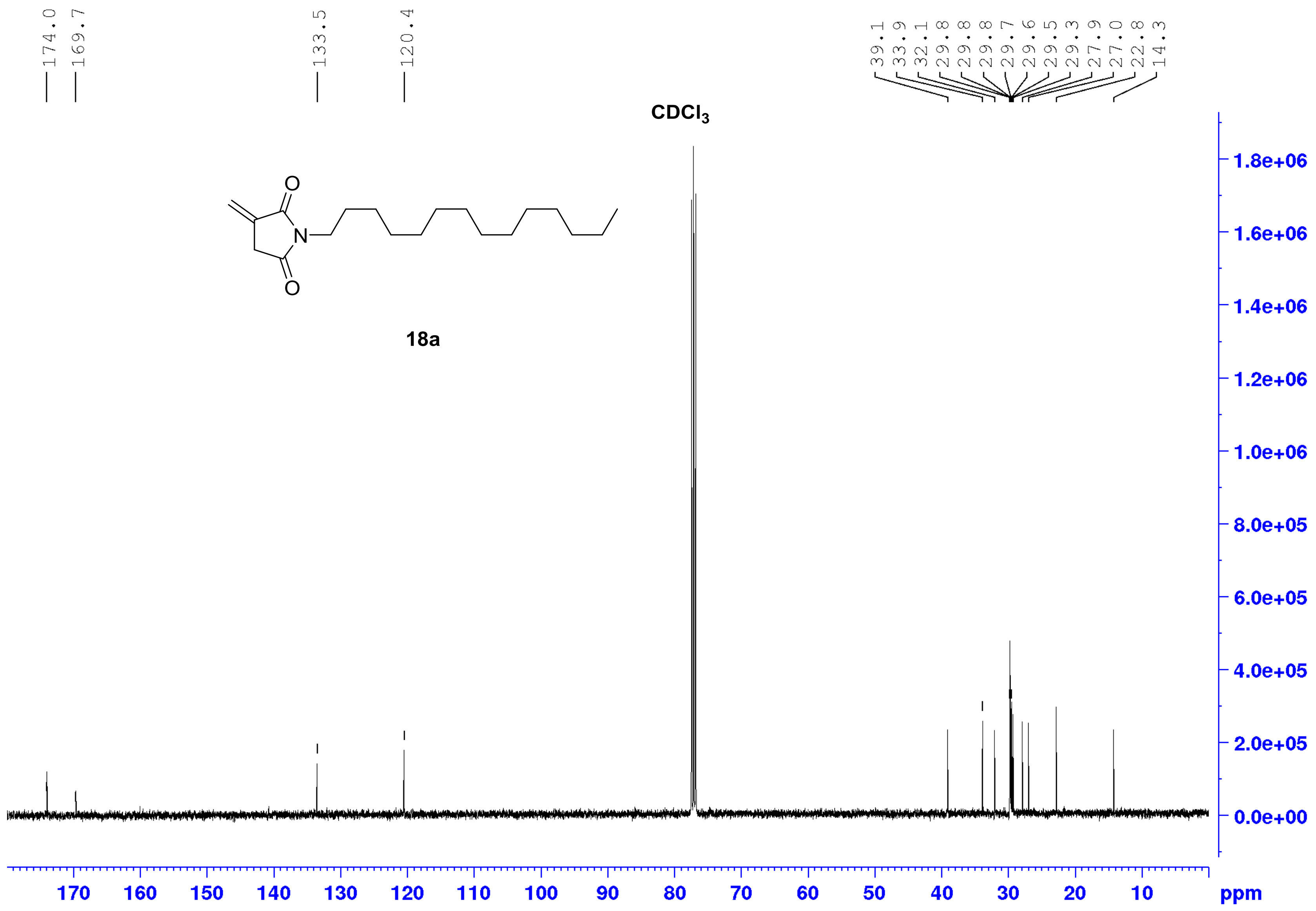
174.0
169.7
133.5
120.4
39.1
33.9
32.1
29.8
29.8
29.8
29.7
29.6
29.5
29.3
27.9
27.0
22.8
14.3
CDCl$_3$
O
N
O
18a
1.8e+06
1.6e+06
1.4e+06
1.2e+06
1.0e+06
8.0e+05
6.0e+05
4.0e+05
2.0e+05
0.0e+00
170
160
150
140
130
120
110
100
90
80
70
60
50
40
30
20
10
ppm

^{1}H- and ^{13}C-NMR spectra of compound **18b**

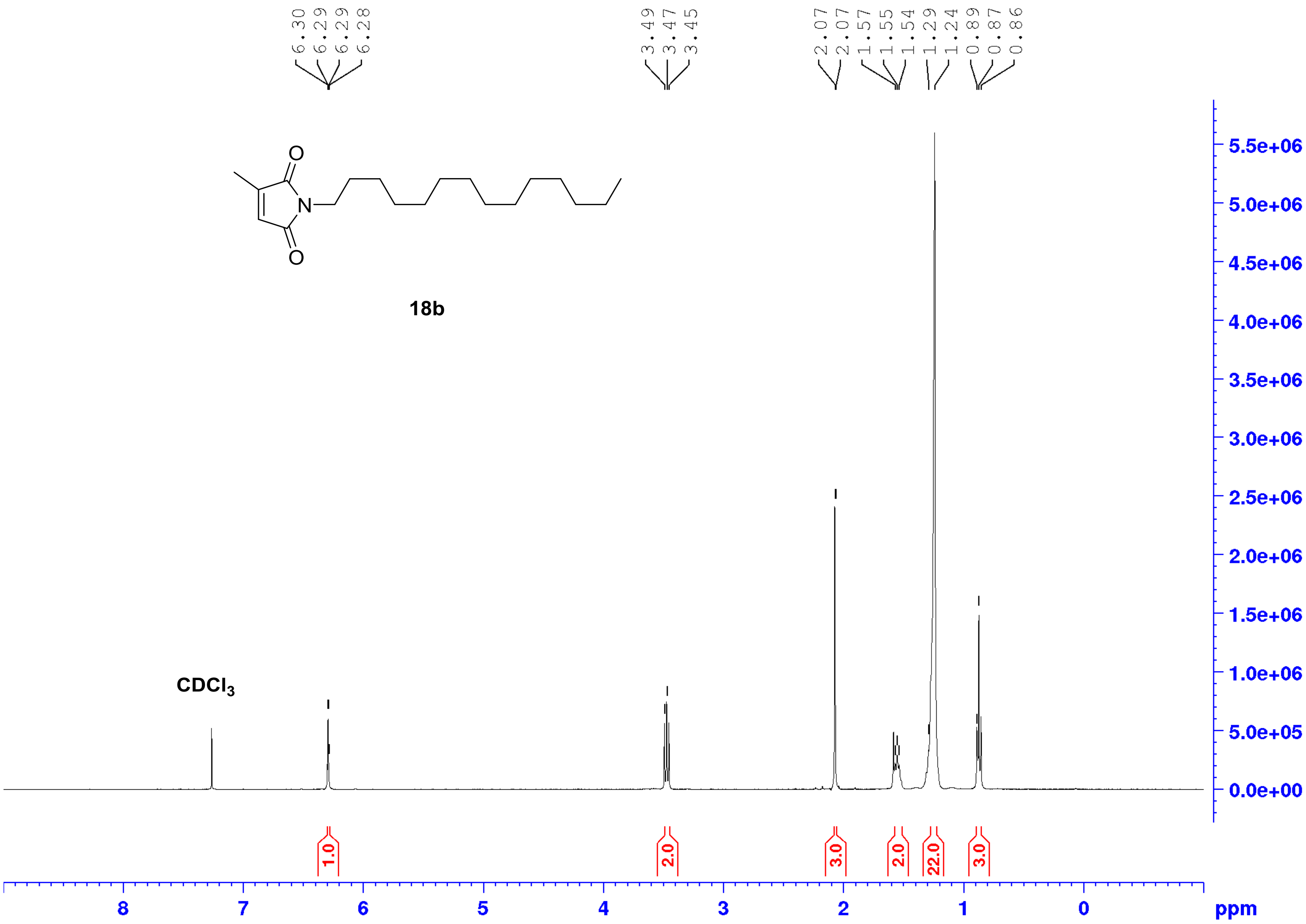

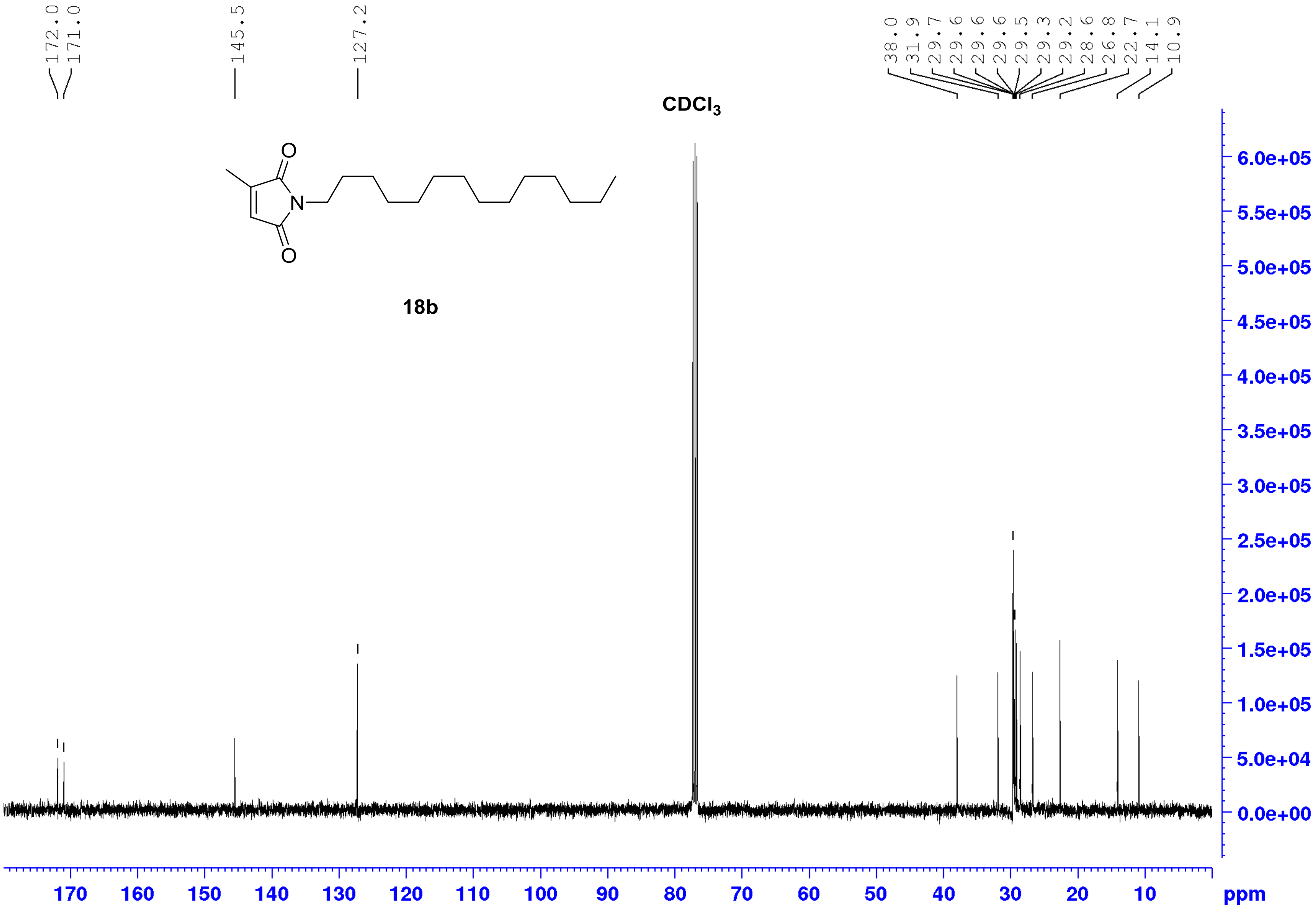

172.0
171.0
145.5
127.2
38.0
31.9
29.7
29.6
29.6
29.6
29.5
29.3
29.2
28.6
26.8
22.7
14.1
10.9
CDCl$_3$
O
N
O
18b
6.0e+05
5.5e+05
5.0e+05
4.5e+05
4.0e+05
3.5e+05
3.0e+05
2.5e+05
2.0e+05
1.5e+05
1.0e+05
5.0e+04
0.0e+00
170
160
150
140
130
120
110
100
90
80
70
60
50
40
30
20
10
ppm
